# Unintended health and societal consequences of international travel measures during the COVID-19 pandemic: a scoping review

**DOI:** 10.1093/jtm/taab123

**Published:** 2021-08-09

**Authors:** Carmen Klinger, Jacob Burns, Ani Movsisyan, Renke Biallas, Susan L Norris, Julia E Rabe, Jan M Stratil, Stephan Voss, Katharina Wabnitz, Eva A Rehfuess, Ben Verboom, the CEOsys Consortium

**Affiliations:** Chair of Public Health and Health Services Research, Institute for Medical Information Processing, Biometry, and Epidemiology - IBE, LMU Munich, Elisabeth-Winterhalter-Weg 6, 81377 Munich, Germany; Pettenkofer School of Public Health, Chair of Public Health and Health Services Research, LMU Munich, Elisabeth-Winterhalter-Weg 6, 81377 Munich, Germany; Chair of Public Health and Health Services Research, Institute for Medical Information Processing, Biometry, and Epidemiology - IBE, LMU Munich, Elisabeth-Winterhalter-Weg 6, 81377 Munich, Germany; Pettenkofer School of Public Health, Chair of Public Health and Health Services Research, LMU Munich, Elisabeth-Winterhalter-Weg 6, 81377 Munich, Germany; Chair of Public Health and Health Services Research, Institute for Medical Information Processing, Biometry, and Epidemiology - IBE, LMU Munich, Elisabeth-Winterhalter-Weg 6, 81377 Munich, Germany; Pettenkofer School of Public Health, Chair of Public Health and Health Services Research, LMU Munich, Elisabeth-Winterhalter-Weg 6, 81377 Munich, Germany; Chair of Public Health and Health Services Research, Institute for Medical Information Processing, Biometry, and Epidemiology - IBE, LMU Munich, Elisabeth-Winterhalter-Weg 6, 81377 Munich, Germany; Pettenkofer School of Public Health, Chair of Public Health and Health Services Research, LMU Munich, Elisabeth-Winterhalter-Weg 6, 81377 Munich, Germany; Chair of Public Health and Health Services Research, Institute for Medical Information Processing, Biometry, and Epidemiology - IBE, LMU Munich, Elisabeth-Winterhalter-Weg 6, 81377 Munich, Germany; Pettenkofer School of Public Health, Chair of Public Health and Health Services Research, LMU Munich, Elisabeth-Winterhalter-Weg 6, 81377 Munich, Germany; Department of Family Medicine, Oregon Health & Science University, 3181 SW Sam Jackson Park Rd, Portland, OR 97239, USA; Chair of Public Health and Health Services Research, Institute for Medical Information Processing, Biometry, and Epidemiology - IBE, LMU Munich, Elisabeth-Winterhalter-Weg 6, 81377 Munich, Germany; Pettenkofer School of Public Health, Chair of Public Health and Health Services Research, LMU Munich, Elisabeth-Winterhalter-Weg 6, 81377 Munich, Germany; Chair of Public Health and Health Services Research, Institute for Medical Information Processing, Biometry, and Epidemiology - IBE, LMU Munich, Elisabeth-Winterhalter-Weg 6, 81377 Munich, Germany; Pettenkofer School of Public Health, Chair of Public Health and Health Services Research, LMU Munich, Elisabeth-Winterhalter-Weg 6, 81377 Munich, Germany; Chair of Public Health and Health Services Research, Institute for Medical Information Processing, Biometry, and Epidemiology - IBE, LMU Munich, Elisabeth-Winterhalter-Weg 6, 81377 Munich, Germany; Pettenkofer School of Public Health, Chair of Public Health and Health Services Research, LMU Munich, Elisabeth-Winterhalter-Weg 6, 81377 Munich, Germany; Chair of Public Health and Health Services Research, Institute for Medical Information Processing, Biometry, and Epidemiology - IBE, LMU Munich, Elisabeth-Winterhalter-Weg 6, 81377 Munich, Germany; Pettenkofer School of Public Health, Chair of Public Health and Health Services Research, LMU Munich, Elisabeth-Winterhalter-Weg 6, 81377 Munich, Germany; Chair of Public Health and Health Services Research, Institute for Medical Information Processing, Biometry, and Epidemiology - IBE, LMU Munich, Elisabeth-Winterhalter-Weg 6, 81377 Munich, Germany; Pettenkofer School of Public Health, Chair of Public Health and Health Services Research, LMU Munich, Elisabeth-Winterhalter-Weg 6, 81377 Munich, Germany; Chair of Public Health and Health Services Research, Institute for Medical Information Processing, Biometry, and Epidemiology - IBE, LMU Munich, Elisabeth-Winterhalter-Weg 6, 81377 Munich, Germany; Pettenkofer School of Public Health, Chair of Public Health and Health Services Research, LMU Munich, Elisabeth-Winterhalter-Weg 6, 81377 Munich, Germany; Department of Social Policy and Intervention, University of Oxford, Barnett House, 32 Wellington Square Oxford OX1 2ER

**Keywords:** Coronavirus, travel restrictions, border closures, quarantine, screening, testing, adverse effects

## Abstract

**Background/Objective:**

International travel measures to contain the coronavirus disease of 2019 (COVID-19) pandemic represent a relatively intrusive form of non-pharmaceutical intervention. To inform decision-making on the (re)implementation, adaptation, relaxation or suspension of such measures, it is essential to not only assess their effectiveness but also their unintended effects.

**Methods:**

This scoping review maps existing empirical studies on the unintended consequences, both predicted and unforeseen, and beneficial or harmful, of international travel measures. We searched multiple health, non-health and COVID-19-specific databases. The evidence was charted in a map in relation to the study design, intervention and outcome categories identified and discussed narratively.

**Results:**

Twenty-three studies met our inclusion criteria—nine quasi-experimental, two observational, two mathematical modelling, six qualitative and four mixed-methods studies. Studies addressed different population groups across various countries worldwide. Seven studies provided information on unintended consequences of the closure of national borders, six looked at international travel restrictions and three investigated mandatory quarantine of international travellers. No studies looked at entry and/or exit screening at national borders exclusively, however six studies considered this intervention in combination with other international travel measures. In total, 11 studies assessed various combinations of the aforementioned interventions. The outcomes were mostly referred to by the authors as harmful. Fifteen studies identified a variety of economic consequences, six reported on aspects related to quality of life, well-being, and mental health and five on social consequences. One study each provided information on equity, equality, and the fair distribution of benefits and burdens, environmental consequences and health system consequences.

**Conclusion:**

This scoping review represents the first step towards a systematic assessment of the unintended benefits and harms of international travel measures during COVID-19. The key research gaps identified might be filled with targeted primary research, as well as the additional consideration of gray literature and non-empirical studies.

## Background

The novel coronavirus SARS-CoV-2 (severe acute respiratory syndrome coronavirus 2), which causes coronavirus disease of 2019 (COVID-19), has spread to every country of the world since its identification in Wuhan in December 2019 and the declaration by the World Health Organization (WHO) of a global pandemic in March 2020.^1^ Non-pharmaceutical interventions (NPIs) have constituted the primary response to the virus by national governments over the first year of the pandemic,^2^ with highly variable success across the globe.^3–5^ NPIs comprise measures that can be implemented at the individual and population level (e.g. physical distancing, face masks, school closures and hand hygiene) to contain the spread of a disease.^6^ Among these, measures affecting human travel across national borders, designed to contain the COVID-19 pandemic (hereinafter referred to as ‘international travel measures’) have been implemented in various combinations since the early stages of the pandemic. These range from the relatively non-intrusive, such as entry and exit screening at national borders, to the more severe, such as travel bans and the complete closure of national borders.^7^ A Cochrane rapid review assessed the effectiveness of international travel measures in the context of the COVID-19 pandemic,^8^ finding an expansive and heterogeneous evidence base suggesting (albeit with low to very low certainty) that some of these measures have a positive impact on infectious disease-related outcomes. However, none of the included studies reported on adverse effects or unintended consequences, a gap that is likely, in part, an artefact of the review’s reliance on biomedical databases, its exclusion of qualitative evidence and the failure or inability of most studies of disease transmission (notably modelling studies) to examine broader societal outcomes.

All social interventions and policy measures can be expected to generate unanticipated consequences.^9^ COVID-19 NPIs are no different. However, the ‘unintended consequences’ of these measures can be understood to include both predictable and unpredictable outcomes, as described by Turcotte-Tremblay *et al*.,^10^ whose definition of unintended consequences encompasses all types of desirable (i.e. beneficial), neutral and undesirable (i.e. adverse/harmful) effects that may be anticipated or unanticipated.

The implementation of international travel measures directly impinges on the right to freedom of movement and therefore places limitations on the enjoyment of many other human rights,^11^ and may result in a range of other unintended and potentially harmful consequences.^10^^,^^12^ The targeted closure of national borders—for instance, to travellers from specific countries—may stoke stigma and xenophobia, leading to discrimination and harassment of people from (or thought to be from) the targeted countries.^13^^,^^14^ The potential negative impact of quarantine on mental health is well established, with the most commonly reported psychological effects including anger, anxiety, boredom, confusion, loneliness and symptoms of post-traumatic stress.^13–15^ Quarantine can also lead to further social consequences, such as food insecurity, reduced healthcare access, financial insecurities, interruption of education and domestic violence.^14^ Virtually all travel measures, either by design or as a side-effect, reduce cross-border travel volumes, potentially generating negative economic consequences^16^ and beneficial environmental effects, including reductions in greenhouse gas emissions and other pollutants related to long-distance travel.^17^^,^^18^ These unintended effects—just like harms associated with the broader family of COVID-19 mitigation and control policies—are likely to be unequally distributed across population groups and may disproportionately impact the most vulnerable in society, potentially exacerbating existing inequities and/or creating new ones.^19^

With many countries now well into their third wave of SARS-CoV-2 infections,^20^ with universal vaccination still in the distant future for most of the world,^21^^,^^22^ and the emergence of more efficiently transmissible viral variants,^23^ it remains crucial to understand the broader health and systemic consequences of these measures to inform decisions on their further (re)implementation, adaptation, relaxation or suspension.^24^

The objective of this scoping review was to comprehensively identify and map the empirical evidence on unintended consequences—both beneficial and harmful—related to measures affecting human travel across national borders, designed to control and/or mitigate the COVID-19 pandemic. The aim was to develop a preliminary understanding of the range of these consequences, as well as the study designs and methodological approaches used to assess them, and to identify gaps in the evidence base.

## Methods

Scoping reviews represent a relatively new approach to evidence synthesis and are often conducted as a precursor to a systematic review. They are mainly used to determine the types and volume of the evidence available in a given field but can also help to clarify key concepts in the literature, investigate how research is conducted on a particular topic, and identify existing knowledge gaps.^25^^,^^26^ The protocol for this review was registered with the Open Science Framework (osf.io/7gyxe) and is available in Appendix 1, Supplementary data at *JTM* online. We followed established methodological guidance on the conduct of scoping reviews,^25^ and we report this review in compliance with the preferred reporting items for systematic reviews and meta-analyses (PRISMA) extension for scoping reviews (Appendix 2, Supplementary data are available at *JTM* online).^27^

### Logic model

**Figure 1:**
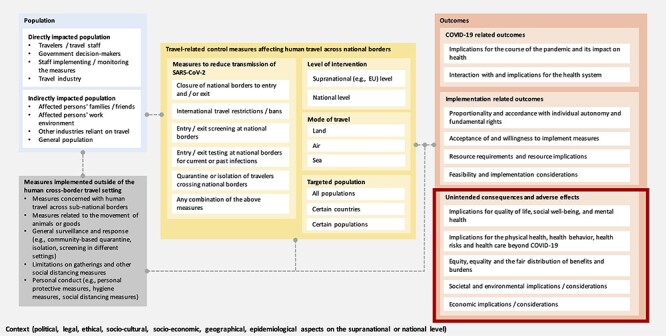
A priori system-based logic model of international travel measures following the PICO (population, intervention, comparison and outcome) scheme.

In an *a priori* system-based logic model (Figure 1) we describe how different travel measures, directly or indirectly affecting various populations, could influence a variety of outcome categories. The present review focuses on a subset of outcomes only, namely the unintended health and societal consequences and adverse effects of these measures. This understanding was informed by (i) three methodological publications on the use of logic models,^28–30^ (ii) two frameworks to facilitate evidence-based decision-making during the COVID-19 pandemic,^12^^,^^31^ (iii) a Cochrane rapid review assessing the effectiveness of travel-related control measures in the context of COVID-19 ^32^ and (iv) discussions within the research team. During the review process, some adaptations were made to the logic model, notably we split ‘social and environmental implications’ into two separate outcome categories, and identified an additional distinct outcome category, namely ‘interaction with and implications for the health system’*.*

### Criteria for considering studies for this review

The inclusion and exclusion criteria for this review are summarized in Table 1 with details provided in Appendix 1, Supplementary data are available at *JTM* online. In brief, to be eligible for inclusion, a study had to (i) be either published in a peer-reviewed academic journal or available on a pre-print server; (ii) report some form of data, either in a quantitative, qualitative, or mixed-methods study; (iii) focus on SARS-CoV-2/COVID-19; (iv) report on at least one international travel measure and (v) provide at least one finding related to an unintended consequence of one or more of these measures. Studies were not excluded on the basis of language.

**Table 1:** Inclusion and exclusion criteria for scoping review

	Inclusion criteria	Exclusion criteria
Publication type	Articles in peer-reviewed academic journals or pre-print servers	All other publication venues, including books, book chapters and gray literature
Study design	Any study design that reports primary empirical data, including: • Quantitative studies (including mathematical modelling studies) • Qualitative studies (if qualitative methods are used both for data collection and analysis^65^) • Mixed-methods studies (i.e. that combine qualitative and quantitative components)	Non-empirical works, including editorials, commentaries, essays, blogs, reports, etc. and literature reviews (systematic or otherwise)
Health condition	Studies of international travel-related control measures for the prevention or control of SARS-CoV-2/COVID-19	All other viruses, diseases and conditions (unless studied alongside SARS-CoV-2/COVID-19)
Intervention types	Travel-related measures, or combinations thereof, in the following categories: • Closure of national borders • International travel restrictions (including targeted entry or exit restrictions, refusal of travel permits, and any other effort to reduce cross-border travel by air, land or sea) • Entry or exit screening (e.g. questionnaires, physical examination, temperature screening, testing, passive observation) • Quarantine for cross-border travellers	• Combinations of travel measures with other interventions (unless effects of travel measures can be isolated) • Measures related to domestic (i.e. not cross-border) travel • Measures limiting movement of animals or goods
Outcomes	Reports on at least one unintended consequence of travel-related control measures, defined as: an effect, either positive or negative, which is neither an intended outcome, or part of how the intervention is expected to achieve its desired outcomes.	• Intended intervention outcomes • Intended intermediate outcomes (i.e. part of hypothesized causal chain)
Language	All languages	N/A

### Identification of relevant studies

We searched the following general and COVID-19-specific databases on 12 December 2020, with the aim of including at least one database covering each of the fields or disciplines of medicine, psychology, sociology, economy and environmental sciences: Ovid MEDLINE, Ovid Embase, Business Source Complete, GreenFILE, APA PsycINFO, Web of Science—SSCI, Web of Science—SCI-EXPANDED, Cochrane COVID-19 Study Register (https://covid-19.cochrane.org/) and WHO Global literature on coronavirus disease database (https://search.bvsalud.org/global-literature-on-novel-coronavirus-2019-ncov/).

The initial search strategy was developed for Ovid MEDLINE and adapted for the other databases (Appendix 3, Supplementary data are available at *JTM* online). We conducted backward citation searches of 96 relevant reviews, commentaries and discussion papers (Appendix 4, Supplementary data are available at *JTM* online), backward and forward citation searches of all included studies (aided by Web of Science, Microsoft Academic and Google Scholar) and contacted experts and study authors regarding missing information.

### Data collection and analysis

#### Selection of studies

After de-duplication, titles and abstracts were screened in duplicate (BV, CK, KW, JB, JER, RB and SV) with a discussion of all unclear cases between the two reviewers or, if necessary, within the broader review team. For all studies deemed potentially relevant or unclear at the title/abstract screening stage, two reviewers screened the full text in duplicate (AM, BV, CK, KW, JB, JER, RB, SLN and SV). Discrepancies were discussed by the two reviewers, and any unclear cases were discussed with a third reviewer and/or the larger review team. At this stage, a final decision regarding inclusion was made, and reasons for exclusion were documented.

We used EndNote to manage the collection and de-duplication of records. For title and abstract screening, we used the web-based application Rayyan (https://rayyan.qcri.org/).

#### Extraction and charting of data

One reviewer (CK) extracted and charted study characteristics and data on setting and context, population, interventions and outcomes from all included studies using a bespoke data extraction form in Microsoft Excel, with findings extracted as reported within the study papers themselves (e.g. effect estimates with confidence intervals and qualitative findings). The extraction form (Appendix 5, Supplementary data are available at *JTM* online) was pilot-tested by the review team on four studies before being discussed and revised as needed. One reviewer (JMS) reviewed all extracted data.

#### Collation, summary and reporting of results

One reviewer (CK) collated, summarized and reported the extracted data narratively, graphically and in tabular form based on the following predefined and inductively adapted categories of unintended consequences: (i) ‘quality of life, well-being and mental health’, (ii) ‘physical health, health behaviour, health risks and healthcare beyond COVID-19’, (iii) ‘equity, equality and the fair distribution of benefits and burdens’, (iv) ‘social consequences’, (v) ‘environmental consequences’, (vi) ‘economic consequences’ and (vii) ‘consequences for the health system’ (see Figure 1).

First, we developed a general summary of the study designs, settings and contexts, populations, interventions and outcomes in each of the included studies. The findings were then presented in the form of a graphical evidence map, with the cells of the map—each representing a specific intervention-outcome category combination—populated by information describing the number and types of studies, if any, that examined that intervention-outcome pair. This was followed by a narrative summary of the findings as reported by the study authors, including effect estimates where available.

## Results

### Search results

**Figure 2:**
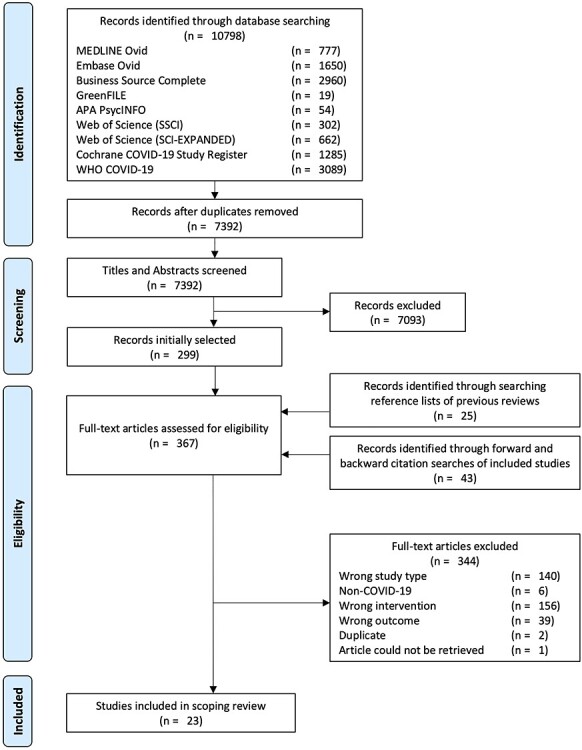
PRISMA flow chart.

The study selection process is summarized in the flow diagram in Figure 2.^27^ Our database searches yielded 7392 unique records after de-duplication. Following the screening of titles and abstracts, 299 articles were retained for full text review. Twenty-five additional records identified through searching the reference lists of previous reviews and 43 records identified through forward and backward citation searches of included studies brought the total number of full texts reviewed to 367. Of these, 23 studies met our inclusion criteria (19 journal articles and 4 pre-prints). Excluded studies and reasons for their exclusion are listed in Appendix 6, Supplementary data are available at *JTM* online.

### Description of included studies

This section provides a brief overview of the included studies in terms of their study designs, settings and contexts, populations, interventions and outcomes. The characteristics of the individual studies are also summarized in Table 2.

**Table 2:** Characteristics of included studies; in case a study reported on several populations, interventions and/or outcomes, the scenarios that belong together were marked alphabetically (A,B,C,…)

Study ID	Study design and methods	Setting and context	Population	Travel-related control measure(s)	Outcome(s)	Notes
Abideen (2020)^42^	Quantitative (observational) study Description: Regression analysis of cross-sectional survey data on the survival of small and medium enterprises (SMEs)	Countries implementing the measure(s): not reported Countries restricted by the measure(s): not reported Mode of travel: not specified	Targeted population: not reported Studied population: 261 owners of SMEs in Abeaokuta, Ogun State (Nigeria)	International travel restrictions Description: International travel restrictions and international movement restrictions (no further definition provided) Date of implementation: not reported	Economic Outcome: Survival of SMEs Length of follow-up: not reported	Study period: not reported CoI: not reported Funding: not reported
B.C. (2020)^52^	Mixed methods study (just quantitative component considered) Description: Mixed method study, based on the descriptive analysis of *n* = 441 cross-sectional survey data and thematic analysis of key-informant interviews	Countries implementing the measure(s): Nepal Countries restricted by the measure(s): India Mode of travel: Land	Targeted population: Returnee migrants from India entering the Karnali province of Nepal Studied population: 441 returnee migrants in various quarantine centres of Surkhet district (63.9% aged 18–36 years; 90% labour workers; 2% higher education (>12 grades); 93% less than 200 USD income per month)	Quarantine or (self)isolation Description: Mandatory quarantine of at least 14 days after entering the Karnali province in institutional quarantine centres of western Nepal Date of implementation: not reported	Quality of life, well-being and mental health Outcome 1: Anxiety Outcome 2: Depression Length of follow-up: not reported	Study period: 12 April – 15 May 2020 CoI: ‘All authors declare that they have no competing interests.’ Funding: ‘This study was funded by Ministry of Social Development (MoSD), Karnali Province of Nepal’
Bombelli (2020)^33^	Quantitative (quasi-experimental) study Description: Interrupted time series analysis based on global aviation data	Countries implementing the measure(s): USA (A); not reported (B) Countries restricted by the measure(s): China (A); not reported (B) Mode of travel: not specified	Targeted population: Foreign nationals that have been to China within the prior two weeks (A); not reported (B) Studied population: Six cargo operators: FedEx, UPS, DHL, Cargolux, Cathay Pacific Cargo, KLMP	International travel restrictions Description: US ban against foreign nationals if they have been to China within the prior two weeks (A) Date of implementation: 31 January 2020 Closure of national borders Description: Closure of European borders (B) Date of implementation: not reported	Economic Outcome (A & B): Air cargo capacity (AFT = available freight tonnes) Length of follow-up: 31 January–18 June 2020 (A); not reported (B)	Study period: 20 November 2019–18 June 2020 CoI: not reported Funding: not reported
Cetin (2020)^34^	Quantitative (quasi-experimental) study Description: Interrupted time series analysis of longitudinal stock market data of the Turkish stock exchange	Countries implementing the measure(s): not reported (unclear, whether the measure refers to incoming or outgoing travel restrictions) Countries restricted by the measure(s): not reported (unclear, whether the measure refers to incoming or outgoing travel restrictions) Mode of travel: not specified	Targeted population: not reported Studied population: Turkey’s stock market	Combination Description: The following restrictions on international travel (citizens exempted) were included in the analysis: • Screening upon arrival (not further specified) • Quarantine of arrivals from some or all regions (not further specified) • Entry restrictions in terms of banning arrivals from some regions (not further specified) • Total border closure or ban on all regions Date of implementation: not reported	Economic Outcome 1: Stock market performance (measured by the closing price, opening price, lowest price and highest price of Turkey’s most important stock market index (BIST-100)) Outcome 2: Economic/Business activity (measures by Turkey’s Purchasing Managers’ Index (PMI)) Length of follow-up: not reported	Study period: 23 March–24 April 2020 CoI: not reported Funding: not reported
Günay (2020)^53^	Quantitative (modelling) study Description: Simple mathematical model exploring different scenarios for the change in foreign visitors as a consequence of the travel restrictions based on assumptions about the travel behaviour following the closure	Countries implementing the measure(s): Turkey Countries restricted by the measure(s): not reported Mode of travel: not specified	Targeted population: Foreign visitors traveling to Turkey Studied population: Foreign visitors traveling to Turkey	Closure of national borders Description: Two scenarios • Decline in demand at the same level compared with the same month of the previous year after the opening of the borders (A) • Decline in demand will recover with equal proportion month by month after the opening of the borders (B) Date of implementation: Between 3 February 2020 (China) and 28 March 2020 (all commercial passenger flights)	Economic Outcome 1 (A & B): Estimated number of visitors/annual change rate Outcome 2 (A & B): Tourism revenue Length of follow-up: Consideration of different scenarios • Border closure for one month • Border closure for 45 days • Border closure for two months • Border closure for three months • Border closure for four months	Study period: January–December 2020 CoI: not reported Funding: not reported
Hu_MR (2020)^41^	Quantitative (quasi-experimental) study Description: Difference-in-Difference study based on longitudinal data of 98 AirBnB markets in 30 countries using government policies on lockdown and travel bans as intervention	Countries implementing the measure(s): 30 countries worldwide Countries restricted by the measure(s): not reported Mode of travel: not specified	Targeted population: International tourists (non-residents) traveling to one of the 98 AirBnB markets in 30 countries Studied population: 98 AirBnB markets	International travel restrictions Description: In-bound travel ban restricting direct access from the countries that represent the home countries of the top 10 international tourist groups Date of implementation: Various—between 9 March 2020 (Italy) and 3 April 2020 (Japan)	Economic Outcome: Impact on the tourism market (using booking activities as a surrogate parameter (number of reviews and cancellation rates as proxies)) Length of follow-up: not reported	Study period: 1 January 2019–31 March 2020 CoI: not reported Funding: not reported
Hu_Y (2020)^46^	Qualitative study Description: Thematic analysis (not further described) of key-informant interviews	Countries implementing the measure(s): China Countries restricted by the measure(s): UK Mode of travel: Air	Targeted population: Travelers to China Studied population: 16 Chinese (undergraduate, non-graduating and final year) international students from mainland China, Hong Kong and Macau, who remained in the UK at the end of March 2020 after the country’s national lockdown and 8 parents in China	Combination Description: China’s tightened border control, quarantine measures and flight constraints Date of implementation (amongst others): • 25 March 2020: Hong Kong border closure • 26 March 2020: ‘five-one’ flight reduction policy (Chinese airlines are only allowed to maintain one international flight per week)	Quality of life, well-being and mental health Outcome 1: Anxiety and stress Length of follow-up: not reported Equity/Equality Outcome 2: Social inequality Length of follow-up: not reported Social Outcome 3: Stigmatization Length of follow-up: not reported	Study period: 7 April–7 May 2020 CoI: ‘The authors declare no conflict of interest regarding this article.’ Funding: ‘This work was supported by the National Social Science Fund of China [grant number: 18CSH011].’
Kaczmarek (2021)^35^	Quantitative (quasi-experimental) study Description: Interrupted time series analysis of longitudinal stock market data of 1201 tourism firms	Countries implementing the measure(s): 52 countries (not specified) Countries restricted by the measure(s): not reported Mode of travel: not specified	Targeted population: not reported Studied population: 1201 stock market-listed tourism companies across 52 countries	Combination Description: The following restrictions on international travel (citizens exempted) were included in the analysis • Screening upon arrival (not further specified) • Quarantine of arrivals from high-risk regions (not further specified) • Total travel restrictions to high-risk regions (‘traveling to high-risk regions is banned’; not further specified) Date of implementation: not reported	Economic Outcome: Corporate immunity of travel and leisure companies to the COVID-19 pandemic (measured by the weekly stock returns) Length of follow-up: not reported	Study period: 6 January–23 March 2020 CoI: ‘none.’ Funding: not reported
Keane (2020)^44^	Quantitative (modelling) study Description: Hybrid model based on longitudinal data sets from 54 countries to estimate the impact of policy change and the spread of COVID-19 on consumer panic (panic buying)	Countries implementing the measure(s): 54 countries were included in the analysis; whereas some enacted international travel restrictions against China, Iran, Italy, or South Korea; others did not Countries restricted by the measure(s): China, Iran, Italy, South Korea, and others (not specified) Mode of travel: Not specified	Targeted population: General population Studied population: General population of 54 countries worldwide	Combination Description: Presence of one or more of the following measures: • Restricting the access of travellers from China, South Korea, Iran or Italy/of non-citizens from anywhere entering the country/of citizens from anywhere entering the country • 14-day quarantine for travellers from China, South Korea, Iran or Italy/for non-citizens from anywhere entering the country/for citizens from anywhere entering the country Date of implementation: Various—between the end of January and the beginning of April 2020, with a peak frequency in mid-March 2020	Economic Outcome: Google searches for key terms as a surrogate parameter for consumer panic Length of follow-up: not reported	Study period: 1 January–30 April 2020 CoI: not reported Funding: not reported
Martuscelli (2020)^47^	Qualitative study Description: Qualitative, phenomenological study	Countries implementing the measure(s): Brazil Countries restricted by the measure(s): not reported Mode of travel: Land, air and water	Targeted population: Non-nationals (with some exemptions) Studied population: 29 refugees living in the states of São Paulo (93%) and Rio de Janeiro (7%); community leaders, activists and their contacts; 86% male; aged 20–48 years	Closure of national borders Description: Closure of borders to non-nationals in Brazil for 30 days (Portaria No 47)—renewed in April for another 30 days and revoked on May 22 through Portaria No 255—prohibiting the entrance of non-nationals to Brazil for another 30 days Date of implementation: 26 March 2020	Quality of life, well-being and mental health Outcome: Anxiety Length of follow-up: 26 March 2020–6 April 2020	Study period: 27 March–6 April 2020 CoI: not reported Funding: not reported
Marzantowicz (2020)^54^	Mixed methods study Description: Descriptive analysis of quantitative survey data and thematic analysis (not further described) of key-informant interviews	Countries implementing the measure(s): Poland (A); not reported (B); not reported (C) Countries restricted by the measure(s): not reported (A); Poland (B); not reported (C) Mode of travel: not specified	Targeted population: not reported Studied population: 11 managers responsible for supply chain operations (from both—supply and demand side) within the enterprises from the production, trade and services sectors in Poland	Closure of national borders Description: Closure of the Polish border (A); Closure of suppliers’ borders (B) Date of implementation: not reported Combination Description: Border closures and mandatory quarantine (C) Date of implementation: not reported	Economic Outcome (A & B & C): Supply chain disruption Length of follow-up: not reported	Study period: March 2020 CoI: not reported Funding: ‘This research received no specific grant from any funding agency in the public, commercial, or not-for-profit sectors.’
Medeiros (2020)^55^	Mixed methods study (just qualitative component considered) Description: Study using qualitative data from various sources to investigate the effects of border closures on the European integration project, and quantitative survey data to expose existing cross-border barriers in Europe	Countries implementing the measure(s): Several European countries (not listed in detail) Countries restricted by the measure(s): Most European countries (not listed in detail) Mode of travel: not specified	Targeted population: Individuals living in regions with strong cross-border mobility and/or cross-border workers/travellers within the European Union Studied population: • Commuters crossing the border for work related purposes on a regular basis and their families (A;C) • Regions with strong cross-border connections of healthcare facilities and health systems (B) • Cross-border commuters, working migrants, and travellers within the European Union (D) • European cross-border areas (E) • Working migrants within the European Union (F)	Closure of national borders Description: Covidfencing in the European Union, i.e. systematic closing of national borders to the circulation of people (A–E) Date of implementation: Multiple Combination Description: Closure of national borders within the European Union and mandatory quarantine (F) Date of implementation: Multiple	Social Outcome 1 (A): Individual and family-level economic security; unemployment Outcome 2 (C): Stigmatization Outcome 3 (D): Cross-border transportation/accessibility Outcome 4 (F): Shifts in economic migration to circumvent quarantine requirements Length of follow-up: not reported Health system Outcome 5 (B): Strains on health systems Length of follow-up: not reported Economic Outcome 6 (E): Health of businesses; commercial economic activity Length of follow-up: not reported	Study period: not reported CoI: ‘No potential conflict of interest was reported by the author(s)’. Funding: not reported
Narayan (2020)^36^	Quantitative (quasi-experimental) study Description: Interrupted time series study, using longitudinal data of the stock markets in G7 countries	Countries implementing the measure(s): G7 countries (Canada, France, Germany, Italy, Japan, UK, USA) Countries restricted by the measure(s): not reported Mode of travel: not specified	Targeted population: not reported Studied population: Stock markets in G7 countries (Canada, France, Germany, Italy, Japan, UK, USA)	Closure of national borders Description: Travel bans (closing international borders) Date of implementation: • Canada: 16 March 2020 • France: 17 March 2020 • Germany: 17 March 2020 • Italy: 10 March 2020 • Japan: 1 February 2020 • UK: 25 March 2020 • USA: 31 Jan 2020	Economic Outcome: Stock market returns Length of follow-up: not reported	Study period: 1 July 2019–16 April 2020 CoI: not reported Funding: not reported
Opilowska (2020)^48^	Qualitative study Description: Hermeneutic content analysis of media reports	Countries implementing the measure(s): Germany, Poland Countries restricted by the measure(s): Germany, Poland Mode of travel: not specified	Targeted population: People crossing the German-Polish border Studied population: Border region residents living close to the German-Polish border	Combination Description: Border closure and mandatory 14-day home quarantine for people crossing the German-Polish border Date of implementation: mid-March 2020	Social Outcome: Cross-border cohesion Length of follow-up: not reported	Study period: 17 March–15 June 2020 CoI: ‘No potential conflict of interest was reported by the author.’ Funding: ‘This work was supported by National Science Centre, Poland [grant number UMO/2018/29/B/HS6/00258]; German-Polish Science Foundation (DPWS), Germany [grant number 2017–09].’
Ozili (2020)^37^	Quantitative (quasi-experimental) study Description: Interrupted time series analysis of longitudinal stock market data	Countries implementing the measure(s): Four countries (Japan, South Africa, UK, US) Countries restricted by the measure(s): not reported Mode of travel: not specified	Targeted population: not reported Studied population: Stock markets of four countries (Japan, South Africa, UK, US)	Combination Description: The following restrictions on international travel (citizens exempted) were included in the analysis • Screening upon arrival (not further specified) • Quarantine of arrivals from high-risk regions (not further specified) • Total travel restrictions to high-risk regions (‘traveling to high-risk regions is banned’; not further specified) Date of implementation: not reported	Economic Outcome 1: Stock market performance (measured by the closing price, opening price, lowest price and highest price from the leading stock market indicators in the four continents—the FTSE 500 index (UK), SP 500 (US), the Nikkei 225 (Japan) and the SA Top 40 index (South Africa)) Outcome 2: Economic/Business activity (measured by the Purchasing Managers’ Index (PMI)) Length of follow-up: not reported	Study period: 23 March–23 April 2020 CoI: not reported Funding: not reported
Pham (2020) ^49^	Qualitative study Description: Qualitative interview study, based on a narrative textual analysis of the transcripts of key-informant interviews	Countries implementing the measure(s): Vietnam, US Countries restricted by the measure(s): not reported Mode of travel: Air (A); not specified (B)	Targeted population: • International travellers from and to Vietnam (A) • International travellers arriving in Vietnam (B) Studied population: 20 Vietnamese students from six different colleges in the New York City area; 8 males, 12 females; aged 19–33 years; stay in the US: 4 months–7 years	International travel restrictions Description: Limitations of international flights from and to Vietnam, registration with the Vietnamese embassy in the USA necessary to buy flight tickets to Vietnam (quota) (A) Date of implementation: not reported Quarantine or (self)isolation Description: Mandatory 14-day quarantine after arriving in Vietnam (B) Date of implementation: not reported	Quality of life, well-being and mental health Outcome 1 (A): Homesickness Length of follow-up: not reported Social Outcome 2 (B): Disruption of work and study due to quarantine Length of follow-up: not reported	Study period: not reported CoI: not reported Funding: not reported
Radic (2020)^50^	Qualitative study Description: Qualitative study, using thematic analysis (unclear, not specified) on transcript of one focus-group discussion	Countries implementing the measure(s): US Countries restricted by the measure(s): not reported Mode of travel: Sea	Targeted population: Cruise ships with the capacity to carry at least 250 passengers in waters subject to US jurisdiction Studied population: Nine cruise ship employees stuck at sea; without current contract/not being paid and without knowing their repatriation date; members of various onboard departments: 7x hotel, 1x marine & technical, 1x entertainment; aged 26–43 years; origin: 5x Asia, 3x Europe, 1x South America; gender: 5x male, 5x female	International travel restrictions Description: CDC No Sail Order that prohibits cruise line companies to use any form of commercial transportation for crew member repatriation purposes, amongst others Date of implementation: 12 April 2020	Quality of life, well-being and mental health Outcome 1: Anxiety and depression Outcome 2: Stress Outcome 3: Social cohesion with the family Length of follow-up: 12 April–22 May 2020 Social Outcome 4: Financial instability Length of follow-up: 12 April–22 May 2020	Study period: 12 April–22 May 2020 CoI: ‘The authors declare no conflict of interest’ Funding: ‘This research received no external funding’
Razak (2020)^51^	Qualitative study Description: Content analysis of newspaper articles	Countries implementing the measure(s): Malaysia Countries restricted by the measure(s): not reported Mode of travel: not specified	Targeted population: Malaysians returning from other countries Studied population: Malaysian travel and accommodation industry	Quarantine or (self)isolation Description: Quarantine of Malaysians coming back from other countries Date of implementation: not reported	Economic Outcome: Income for the accommodation industry Length of follow-up: not reported	Study period: March–May 2020 CoI: not reported Funding: not reported
Solomou (2020)^43^	Quantitative (observational) study Description: Descriptive statistics of quantitative survey data	Countries implementing the measure(s): not reported Countries restricted by the measure(s): not reported Mode of travel: not specified	Targeted population: not reported Studied population: 1642 people over 18 years from Cyprus; 16.1% reported residing abroad at the time of the participation; majority aged 18–60 years	Closure of national borders Description: Border closures resulted in people being trapped abroad Date of implementation: not reported	Quality of life, well-being and mental health Outcome 1: Anxiety Outcome 2: Depression Length of follow-up: Three weeks	Study period: 3 April–9 April 2020 CoI: ‘The authors declare no conflict of interest.’ Funding: ‘This research received no external funding’.
Zaremba (2020)^39^	Quantitative (quasi-experimental) study Description: Interrupted time series analysis of longitudinal stock market data from 67 countries	Countries implementing the measure(s): 67 countries worldwide Countries restricted by the measure(s): not reported Mode of travel: not specified	Targeted population: not reported Studied population: Stock markets of 67 countries worldwide	Combination Description: The following restrictions on international travel (citizens exempted) were included in the analysis • Screening upon arrival (not further specified) • Quarantine of arrivals from some or all regions (not further specified) • Entry restrictions in terms of banning arrivals from some regions (not further specified) • Total border closure or ban on all regions Date of implementation: not reported	Economic Outcome: Stock market volatility Length of follow-up: Three weeks	Study period: 1 January–3 April 2020 CoI: not reported Funding: ‘Adam Zaremba acknowledges the support of the National Science Centre of Poland [grant no. 2016/23/B/HS4/00731].’
Zaremba (2021a)^38^	Quantitative (quasi-experimental) study Description: Interrupted time series analysis of longitudinal daily stock data from 49 countries	Countries implementing the measure(s): 49 developed and emerging countries worldwide Countries restricted by the measure(s): not reported Mode of travel: not specified	Targeted population: not reported Studied population: Stock market of 49 developed and emerging countries worldwide	Combination Description: The following restrictions on international travel (citizens exempted) were included in the analysis • Screening upon arrival (not further specified) • Quarantine of arrivals from some or all regions (not further specified) • Entry restrictions in terms of banning arrivals from some regions (not further specified) • Total border closure or ban on all regions Date of implementation: not reported	Economic Outcome: Global stock market liquidity Length of follow-up: Between 0 and 66 days	Study period: 1 January–3 April 2020 CoI: not reported Funding: not reported
Zaremba (2021b)^40^	Quantitative (quasi-experimental) study Description: Interrupted time series analysis of longitudinal stock market data from 67 countries	Countries implementing the measure(s): 67 countries worldwide Countries restricted by the measure(s): not reported Mode of travel: not specified	Targeted population: not reported Studied population: Stock markets of 67 countries worldwide	Combination Description: The following restrictions on international travel (citizens exempted) were included in the analysis • Screening upon arrival (not further specified) • Quarantine of arrivals from some or all regions (not further specified) • Entry restrictions in terms of banning arrivals from some regions (not further specified) • Total border closure or ban on all regions Date of implementation: not reported	Economic Outcome: Country-level stock market immunity (measured by daily and weekly stock market returns) Length of follow-up: not reported	Study period: 1 January–28 April 2020 CoI: not reported Funding: not reported
Zhang (2020)^45^	Quantitative (modelling) study Description: Apriori algorithm, an unsupervised machine learning method	Countries implementing the measure(s): Almost 187 countries on all continents Countries restricted by the measure(s): not reported Mode of travel: not specified	Targeted population: not reported Studied population: 187 countries—grouped by six continents based on geographic connectivity (Asia, Europe, North America, South America, Africa and Oceania)	International travel restrictions Description: Provincial or state international travel controls Date of implementation: Various—most of them between the end of January 2020 and the end of March 2020, with a peak in mid-March 2020	Environmental Outcome: NO_2_ column density Length of follow-up: not reported	Study period: 1 January–30 April 2020 CoI: ‘The authors declare no conflict of interest.’ Funding: not reported

#### Study design

Of the included studies, nine used quasi-experimental designs, including eight interrupted time series studies^33–40^ and one difference-in-difference study.^41^ We also identified two observational studies,^42^^,^^43^ two mathematical modelling studies,^44^^,^^45^ six qualitative studies ^46–51^ and four mixed methods studies.^52–55^

#### Setting and context

The included studies reported on the implementation of international travel measures in Brazil,^47^ Canada,^36^ France,^36^ Germany,^36^^,^^48^ Italy,^36^ Japan,^36^^,^^37^ Malaysia,^51^ Nepal,^52^ Poland,^48^^,^^54^ South Africa,^37^ Turkey,^53^ Vietnam,^49^ the UK ^36^^,^^37^ and the USA.^33^^,^^36^^,^^37^^,^^46^^,^^49^^,^^50^ Eight studies analysed the effects of international travel measures implemented by multiple countries.^35^^,^^38–41^^,^^44^^,^^45^^,^^55^ Three studies did not focus on specific countries or settings.^34^^,^^42^^,^^43^

Some studies reported on the countries that were targeted by the measures, notably China,^33^^,^^44^ Germany,^48^ India,^52^ Iran,^44^ Italy,^44^ Poland,^48^^,^^54^ South Korea ^44^ and the UK.^46^ However, in the majority of studies, this was either not applicable or not specified.^33–43^^,^^45^^,^^47^^,^^49–51^^,^^53–55^ Most studies did not differentiate between specific modes of travel (i.e. air, land or sea).

#### Population

Studies examined the effects of international travel measures on various populations: stock market listed companies,^34–40^ the tourism industry,^41^^,^^51^^,^^53^ cross-border commuters and working migrants,^52^^,^^55^ border-region residents,^48^^,^^55^ international students,^46^^,^^49^ businesses,^42^^,^^54^ cargo airlines,^33^ healthcare facilities,^55^ refugees,^47^ cruise ship employees^50^ and the general population.^43–45^

#### Intervention

A variety of different travel measures were investigated, often in combination. Seven studies provided information on the closure of national borders,^33^^,^^36^^,^^43^^,^^47^^,^^53–55^ six looked at international travel restrictions,^33^^,^^41^^,^^42^^,^^45^^,^^49^^,^^50^ and three investigated quarantine of travellers crossing national borders.^49^^,^^51^^,^^52^ No studies were identified that looked at entry and/or exit screening at national borders exclusively (i.e. in isolation, rather than in combination with other interventions), but we identified six studies that considered this type of intervention in combination with other travel measures.^34^^,^^35^^,^^37–40^ Eleven included studies reported on unintended consequences of various combinations of these four categories of international travel measures.^34^^,^^35^^,^^37–40^^,^^44^^,^^46^^,^^48^^,^^54^^,^^55^

#### Outcomes

Fifteen studies looked at a variety of ‘economic consequences’,^33–42^^,^^44^^,^^51^^,^^53–55^ whereas six reported on outcomes related to ‘quality of life, well-being and mental health’,^43^^,^^46^^,^^47^^,^^49^^,^^50^^,^^52^ and five reported on ‘social consequences’.^46^^,^^48–50^^,^^55^ One study each provided information on ‘equity, equality and the fair distribution of benefits and burdens’,^46^ ‘environmental consequences’ ^45^ and ‘health system consequences’.^55^ We did not identify any studies reporting on ‘physical health, health behaviour, health risks and healthcare beyond COVID-19’. Details regarding these outcomes are provided in Table 2.

### Graphical summary and evidence gap map

**Figure 3:**
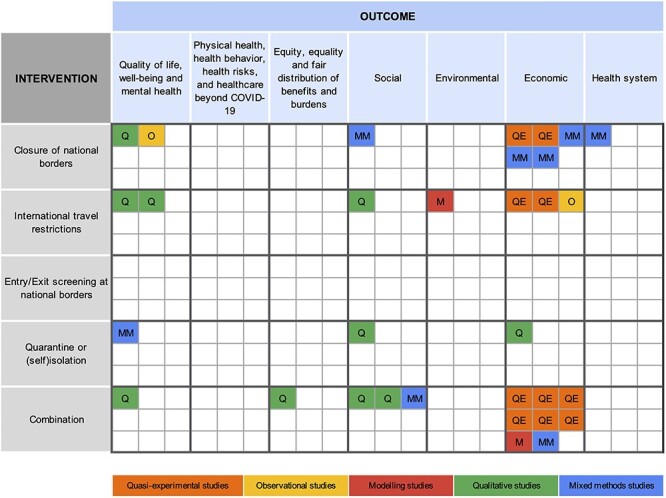
Evidence gap map of different study designs (colour) addressing various international travel measures (rows) and their respective unintended consequences (columns).

The distribution of included studies in relation to the study design, intervention and outcome categories is summarized in an evidence gap map (Figure 3). Some significant gaps are worth noting. We identified no studies that exclusively assessed the unintended consequences of entry/exit screening at national borders. Moreover, no included studies assessed consequences related to ‘physical health’*, ‘*health behaviour’*, ‘*health risks and healthcare beyond COVID-19’. The six studies analysing the effects of international travel measures on ‘quality of life, well-being and mental health’,^43^^,^^46^^,^^47^^,^^49^^,^^50^^,^^52^ as well as the five studies assessing ‘social consequences’,^46^^,^^48–50^^,^^55^ mainly relied on qualitative data. The 15 studies that examined the ‘economic consequences’ of these measures presented outcomes mainly in quantitative terms.^33–42^^,^^44^^,^^51^^,^^53–55^ In general, quasi-experimental methods were used in studies of economic outcomes and not for the assessment of other societal consequences.

‘Economic consequences’ included effects on stock markets,^34–40^ supply chains,^54^ air cargo capacities,^33^ the economic health of businesses,^42^^,^^55^ commercial economic activity,^34^^,^^37^^,^^44^^,^^55^ tourism volumes^41^^,^^53^ and tourism revenue.^53^ ‘Social consequences’ covered financial security and employment,^50^^,^^55^ economic migration,^55^ effects on study and/or work,^49^ cross-border transportation^55^ and discrimination and stigmatization.^46^^,^^55^ Consequences related to ‘quality of life, well-being and mental health’ included anxiety,^43^^,^^46^^,^^47^^,^^50^^,^^52^ depression,^43^^,^^50^^,^^52^ stress^46^^,^^50^ and reduced social cohesion with families and friends.^49^^,^^50^ ‘Health systems’ experienced strains due to the inability of cross-border health workers to reach their place of employment.^55^ A majority of consequences were harmful, but some unintended positive impacts, including e.g. a decrease in NO_2_ emissions,^45^ the promotion of cross-border social cohesion^48^ and potential increases in income for the tourism industry,^51^ were also described.

### Narrative summary of findings

In the following, we provide a narrative summary of the unintended consequences associated with the four categories of international travel measures.

#### Border closures

The closure of national borders was reported to have raised anxiety among Brazil’s refugee population due to uncertainties about the right to family reunification and the inability of relatives to enter Brazil.^47^ In Cyprus, having a first-degree relative trapped abroad was found to be associated with higher anxiety levels, and being personally trapped abroad was associated with higher depression levels.^43^

Cross-border commuters in the European Union were confronted with financial insecurity and unemployment as a result of border closures. Furthermore, they reported experiencing discrimination by some border-region residents who were afraid of getting infected. Cross-border commuters, working migrants and travellers within the European Union were faced with reductions in cross-border public transportation and accessibility leading to detours and long waiting times at the few border crossings that remained open.^55^

Intra-European border closures were found to negatively affect the health of businesses and commercial economic activity in European cross-border regions.^55^ The closure of European borders in mid-March 2020 was also associated with reduced air cargo capacities for the European commercial airline KLM Royal Dutch Airlines.^33^ The closure of the Polish border was identified as an important contributor to supply chain disruption in manufacturing, trading and service companies in Poland, as was the closure of their suppliers’ borders.^54^ Another study predicted that the closure of the Turkish border would lead to a decline in the number of foreign visitors between 10 and 53% in 2020, leading to losses in tourism revenue of between $1.5 and $15.2 billion.^53^ The closure of national borders seemed to have a beneficial effect on the stock market returns of two G7 countries (Canada and Germany).^36^

The re-implementation of border closures within the European Union was reported to impose strains on ‘health systems’ in regions that depend on cross-border health workers, caused by disruptions in their ability to reach their place of employment.^55^

#### International travel restrictions

Limitations on international flights to and from Vietnam during the pandemic, accompanied by compulsory registration with the Vietnamese embassy in order to buy flight tickets, was found to negatively affect the well-being and mental health of Vietnamese students located in the USA.^49^ Being unemployed and stuck at sea due to the US Centers for Disease Control (CDC) No Sail Order was associated with increased levels of stress, anxiety and depression among cruise ship employees, as well as reduced social cohesion with their families and friends.^50^

The CDC No Sail Order, which prevented cruise line companies from using any form of commercial transportation for the repatriation of their crew members, also impacted the financial situation of the crew. Being unemployed and isolated at sea, stranded workers were not able to seek or commence new employment.^50^

One study found that international travel controls were associated with a decrease in air pollution, notably NO_2_ column density—the concentration of NO_2_ along the vertical column of the atmosphere.^45^

International travel restrictions were found to be negatively associated with the survival of small and medium enterprises in Nigeria.^42^ The US entry ban against foreign nationals who had been to China resulted in a sharp drop in air cargo capacity for international shipping companies and full-cargo airlines in early February 2020.^33^ Regarding the tourism industry, inbound travel bans showed a stronger negative effect on AirBnB markets in 30 countries than local confinement.^41^

#### Quarantine of travellers crossing national borders

A mandatory quarantine of at least 14 days in institutional quarantine centres in western Nepal was negatively associated with the mental health of quarantined migrants, with 21% reporting to suffer from anxiety and 14% from depression.^52^

Some Vietnamese students located in the USA feared the social consequences of a mandatory 14-day quarantine for international travellers arriving in Vietnam, as it would disrupt their studies and work, e.g. due to expected poor internet connectivity in quarantine facilities.^49^

The policy of mandatory quarantine for Malaysians returning from abroad is predicted to be beneficial for the accommodation industry, as a result of using Malaysian hotels as government quarantine centers.^51^

#### Combinations of international travel measures

Chinese international students in the UK and their parents in China reported heightened anxiety due to the perceived ‘double-exclusion’ from both countries—the UK and China—experienced by the students, and their difficulty acquiring plane tickets.^46^ The impact of these challenges on families was not experienced equally. Because of changing flight schedules and quarantine restrictions, many families bought several tickets for their children to secure their trip home—a strategy that was only available to wealthier families.^46^

The aforementioned measures were also reported to have made Chinese international students returning from the UK feel stigmatized as they were considered as ‘irresponsible virus carriers’ with Chinese media blaming them for importing COVID-19.^46^ The closure of national borders within the European Union, in combination with a mandatory quarantine, was reported to lead to shifts in economic migration to circumvent quarantine requirements, affecting workers who had previously been cross-border commuters.^55^ The closure of the German-Polish border in mid-March 2020, and the implementation of a mandatory 14-day home quarantine for people crossing the border, was reported to affect cross-border social cohesion. The re-implementation of physical barriers led to a backlash from border-region residents in the form of protests and solidarity actions with the aim of underlining the importance of an united Europe.^48^

In terms of economic consequences, the combination of a mandatory 14-day quarantine for travellers and complete entry bans was not associated with consumer panic, i.e. panic buying of storable consumer goods, in 54 countries.^44^ The combination of border closures and mandatory quarantine was reported to disrupt supply chains within the production, trade and service sectors in Poland.^54^ Six studies assessed the effects of combinations of international travel measures on stock markets. Negative associations were reported between the stringency of international travel measures and the closing price and lowest price of Turkey’s most important stock index and Turkey’s Purchasing Managers’ Index,^34^ as well as the stock prices and Purchasing Managers’ indices in Japan, South Africa, the UK and the USA.^37^ No statistically significant associations were identified between the stringency of travel measures and the weekly stock returns of 1201 travel and leisure companies across 52 countries,^35^ stock market volatility and country-level stock market returns in 67 countries ^39^^,^^40^ or stock market liquidity in 49 developed and emerging countries.^38^

## Discussion

This scoping review addressed various travel measures for the prevention and control of COVID-19, namely national border closures, international travel restrictions, quarantine of travellers crossing national borders and combinations of these measures. We did not identify any studies assessing the unintended consequences of entry and/or exit-screening at national borders exclusively.

The majority of studies identified “economic consequences”, but some also reported on effects on ‘quality of life, well-being and mental health’ as well as ‘social consequences’. ‘Equity, equality and the fair distribution of benefits and burdens’, ‘environmental consequences’ and ‘health system consequences’ represented only a small focus among the studies included. Furthermore, we did not identify any studies concerned with effects on ‘physical health’*,* ‘health behaviour, health risks and healthcare beyond COVID-19’. However, some studies that were reviewed at the full text stage^56–58^ and that were eventually excluded because the effects of international travel measures could not be disentangled from other factors, imply that these measures *may* also affect outcomes in the ‘physical health’ domain. For example, Aiken *et al*.^56^ suggest that reduced access to in-clinic abortion services due to movement restrictions may lead to an increase in requests for ‘self-managed’ abortion in Northern Ireland and Malta, the residents of which normally have to travel abroad to access abortion services. The findings from Korun *et al*.^57^ imply that border closures may prevent children in highly-deprived areas from accessing paediatric cardiac surgeries and possibly other health services. Mahmassani *et al*.^58^ identified a decrease in Emergency Department visits after the declaration of a ‘public mobilization state’ that included the closure of borders.

This body of evidence provides an overview of the unintended consequences of international travel measures during the COVID-19 pandemic identified to date. We uncovered disparate findings scattered across 23 studies from several disciplines. Many of these studies were only tangentially concerned with identifying and describing unintended effects of travel measures. Indeed, one of the most important contributions of this scoping review is the bringing together of a patchwork of findings, pointing towards a need for a strategic research agenda addressing the key gaps in the evidence base.

### Strengths, limitations and methodological reflections

Specifying and implementing clear inclusion criteria for a review of the unintended consequences of an intervention from a societal perspective were not straightforward. Both the relatively small number of identified studies and some of the intervention-outcome gaps identified may, in part, be an artefact of our inclusion criteria. For example, during the screening process, we came across several studies examining the effects of broader COVID-19 control measures on the environment, particularly on air pollution. However, these studies did not disentangle the effects of international travel measures from other measures (notably local movement restrictions and lockdowns). Furthermore, many studies did not differentiate between the unintended consequences of international travel measures and the effects of reduced international traffic in general. For example, Bich-Ngoc and Teller^59^ modelled changes in water demand as a function of outbound tourist travel, Liu *et al*.^60^ investigated the effects of reduced international air traffic on CO_2_ emissions, and Sugiura *et al*.^61^ examined the impact of reductions in air travel from China on the importation into Japan of Africa Swine Fever disease.

The strengths of this scoping review include its broad multi-disciplinary search strategy, as well as backward citation tracking of relevant reviews, commentaries and discussion papers. Four of the 23 included studies are preprints, which at the time of writing had not yet undergone peer review. Six studies, all assessing economic outcomes of international travel measures, were published in journals not covered by the databases used.^34^^,^^37^^,^^39–42^ Any future effort to update this review, or to review a similar body of evidence, would therefore benefit from the use of additional databases to increase the chances of identifying all relevant studies. The additional consideration of gray literature and non-empirical publications (e.g. government reports) beyond published empirical data from academic research may have uncovered a more comprehensive evidence base, especially for outcomes difficult to investigate using empirical research designs.^24^^,^^62–64^

During data extraction and mapping, we faced difficulties classifying some outcomes according to our *a priori* defined and inductively adapted categories.^12^ Some unintended consequences may fit into several categories, whereas others did not neatly fit into any of these. For example, a ‘lack of social cohesion’ could be understood as affecting both ‘quality of life’ and ‘social consequences’*.* After some deliberation, we decided to assign negative impacts on social cohesion to the first category when family and friends were affected^50^ and to the second when whole societies were impacted.^48^

We followed a transparent review process and safeguarded rigor through (i) conducting calibration exercises and review team meetings prior to individual screening stages, (ii) developing clear guidance and procedures for team members for all key steps, (iii) addressing questions from team members on a rolling basis and (iv) hosting frequent open discussions within the team. All screening was conducted in duplicate to mitigate the effects of bias and human error. All data extractions were checked for quality and consistency by an experienced reviewer. In line with established methodological practice in scoping reviews,^25^ we did not perform quality appraisals or risk of bias assessments of the included studies.

## Conclusions

This scoping review complements a Cochrane rapid review on the effectiveness of international travel-related control measures^8^ and provides a broad overview and an initial understanding of potential unintended consequences of international travel measures. The 23 included studies examined the effects of various measures—the complete closure of national borders, international travel restrictions, quarantine of travellers crossing national borders, as well as combinations of international travel measures. The consequences observed were mostly harmful with the exception of a few unintended positive impacts. Economic consequences and, to a lesser extent, mental health and social impacts, are relatively well represented in this evidence base, whereas few or no studies examined consequences for health systems, environmental outcomes, impacts on equity and physical health. The key research gaps identified here might be filled with targeted primary research, as well as the additional consideration of gray literature and non-empirical studies, to ensure that the full range of unintended consequences of international travel measures during the COVID-19 pandemic is covered.

## Supplementary Material

20210804_UnintendedConsequencesTravelMeasures_SupplementaryData_taab123

Supplementary_Data_Legends_taab123
