# Supplementary material for: Unintended health and societal consequences of international travel measures during the COVID-19 pandemic: a scoping review
Source: J Travel Med. 2021 Aug 9;28(7):taab123. doi: 10.1093/jtm/taab123 (PMC8436381; doi:10.1093/jtm/taab123)
Supplement: 20210804_UnintendedConsequencesTravelMeasures_SupplementaryData_taab123 [file 20210804_unintendedconsequencestravelmeasures_supplementarydata_taab123.docx]

# Supplementary Data

## Appendix 1. Review protocol

Review protocol

Unintended health and societal consequences of international travel-related control measures during the COVID-19 pandemic: a scoping review (protocol)

Authors:

Carmen Klinger^1,2^, Jacob Burns^1,2^, Ani Movsisyan^1,2^, Renke Biallas^1,2^, Hannah Littlecott^1,2,3^, Lisa M Pfadenhauer^1,2^, Julia E Rabe^1,2^, Jan M Stratil^1,2^, Stephan Voss^1,2^, Katharina Wabnitz^1,2^, Eva A Rehfuess^1,2^, Ben Verboom^1,2,4^

^1^Institute for Medical Information Processing, Biometry and Epidemiology; Ludwig-Maximilians-University Munich, Germany

^2^Pettenkofer School of Public Health, Munich, Germany

^3^DECIPHer, School of Social Sciences, Cardiff University, Cardiff, United Kingdom

^4^Department of Social Policy and Intervention, University of Oxford, Oxford, United Kingdom

Background

In December 2019, the first case of a novel disease caused by the new coronavirus SARS-CoV-2, namely coronavirus disease 2019 (COVID-19), was observed in Wuhan (Hubei province of China). With more than 100 countries worldwide affected by the disease by mid-March 2020, the outbreak was declared a global pandemic by the World Health Organization (WHO) on March 11, 2020 (WHO, 2020).

Given the lack of an effective and widely-available vaccine to prevent SARS-CoV-2 infection, and limited available treatments for COVID-19, a range of non-pharmaceutical interventions have been put into place by national governments to contain and mitigate the global spread of the virus. Travel-related control measures were implemented at a very early stage of the COVID-19 pandemic. These range from relatively non-intrusive measures, such as various forms of entry and exit screening at national borders, to more severe measures, such as travel bans and the complete closure of national borders. As of 1 November 2020, 118 destinations (54% of all destinations worldwide) have completely or partially closed their borders to international tourism. 126 destinations (58%) require the presentation of a negative COVID-19 PCR test result upon arrival. Quarantine and/or self-isolation upon arrival is requested by ten destinations (5%). Twelve destinations (5%) have implemented destination-specific travel restrictions by denying entry to passengers from specific countries of origin. However, since the beginning of the pandemic, 152 destinations (70%) have eased COVID-19 related travel restrictions for international tourism (UNWTO, 2020).

A previous rapid review assessed the effectiveness of travel-related control measures in the context of SARS-CoV-2/COVID-19 (Burns et al., 2020), finding an expansive and heterogeneous evidence base with an overall low- to very low-certainty of evidence. The review suggests that some travel-related control measures implemented during the COVID-19 pandemic have a positive impact on disease-related outcomes, notably on reducing the number of (imported) cases and on delaying or reducing outbreak development. None of the included studies reported on outcomes related to the implementation of travel-related control measures, nor health-related adverse effects or broader societal and systemic implications. This is likely attributable to the fact that studies reporting primary outcomes related to disease transmission do not often look additionally at these broader outcomes, and to the fact that the review searched sources with a primary focus on health.

The implementation of travel-related control measures directly impinges on the right to freedom of movement and, as a consequence, places limitations on the enjoyment of many other human rights (UN, 2020). When deciding whether to institute, implement, or indeed relax travel-related control measures to contain the SARS-CoV-2/COVID-19 pandemic, it is important for policymakers to consider not only the likely effectiveness of such measures with regard to controlling the pandemic, but other factors as well, including the potential for harms and other unintended consequences (Stratil et al., 2020). The targeted closure of national borders – for instance, to travelers from specific countries or regions – may stoke stigma and xenophobia, leading to discrimination and harassment of people from or thought to be from the targeted countries or regions (1). The potential negative impact of quarantine on mental health is well established, with the most commonly reported psychological effects including confusion, anger, anxiety and symptoms of post-traumatic stress (Brooks et al., 2020). Virtually all travel-related control measures, either by design or as an unintended side-effect, are almost certain to contribute to reductions in cross-border travel volumes, generating a combination of economic consequences (Nicola et al., 2020) alongside potential beneficial environmental effects, including reductions in greenhouse gas and nitrogen oxide emissions (Bashir, Benjiang, & Shahzad, 2020; Lal et al., 2020). These unintended effects – just like harms associated with the broader family of COVID-19 mitigation and control policies – are likely to be unequally distributed across population groups and may disproportionately impact the most vulnerable in society, potentially exacerbating existing inequities while creating new ones (Glover et al., 2020).

In an a priori system-based logic model (Figure 1) we elaborate the overall context, describing how the implementation and/or relaxation of different travel-related control measures implemented during the SARS-CoV-2/COVID-19 pandemic, directly or indirectly affecting various populations on individual and organizational level, could influence a variety of possible outcome categories. The present review will focus on a subset of outcomes only, namely the unintended health and societal consequences and concrete adverse effects of these measures. These also depend on measures implemented outside of the human cross-border travel setting, e.g., the use of personal protective equipment.

This understanding was informed by (i) three methodological publications on the use of logic models (Kneale et al., 2015; Rohwer et al., 2017; Rehfuess et al., 2018), (ii) a framework to facilitate evidence-based decision-making during the SARS-CoV-2/COVID-19 pandemic (Stratil et al., 2020), (iii) a Cochrane rapid review assessing the effectiveness of travel-related control measures in the context of SARS-CoV-2/COVID-19 (Burns et al., 2020) and (iv) discussions within the research team. This a priori logic model will be updated with regard to the outcomes based on our findings and new insights gained.


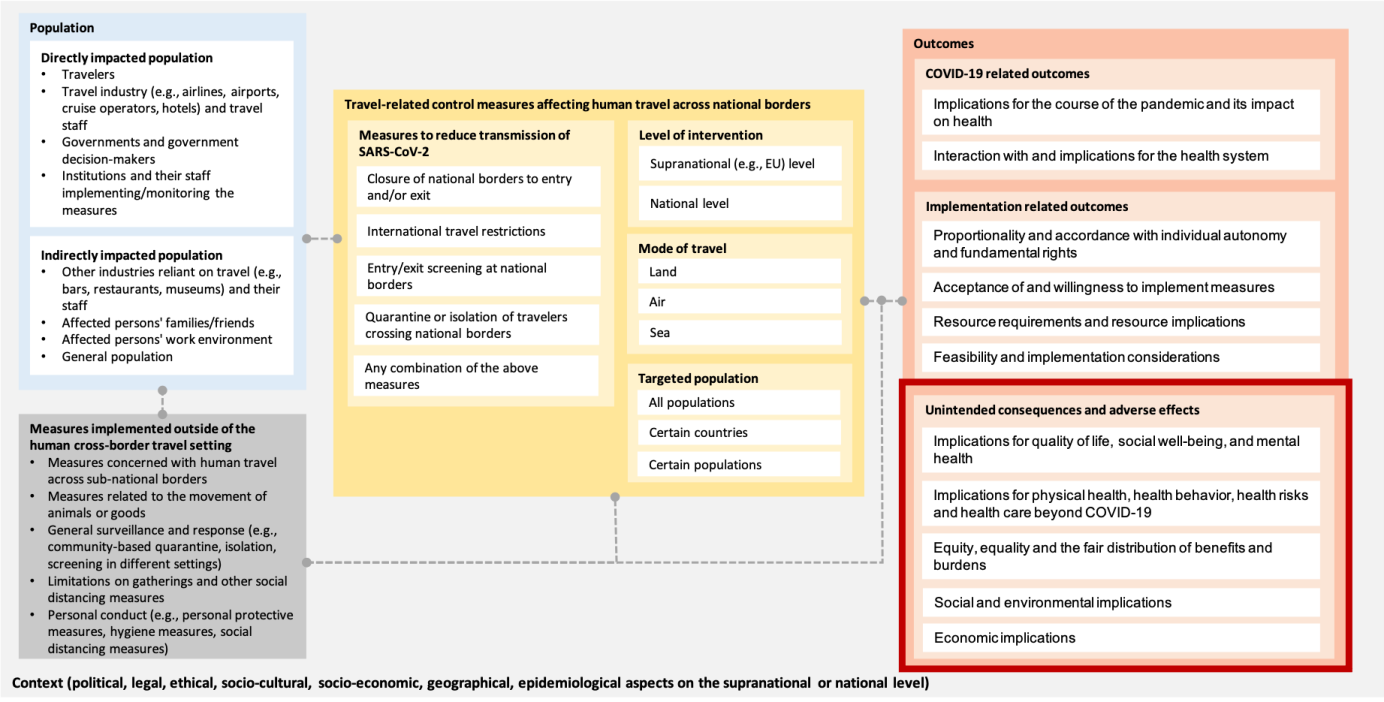


Figure 1: A priori system-based logic model of travel-related control measures affecting human travel across national borders following the PICO (population, intervention, comparison and outcome) scheme.

Many countries are now reporting a second wave of SARS-CoV-2 infections (Looi, 2020), and it remains crucial to understand the broader health, societal and systemic consequences of these measures to inform decisions on their further (re)implementation, adaptation, relaxation or suspension. To our knowledge, as of January 2021, no scoping review, evidence map or systematic review investigating the unintended consequences of travel-related control measures has been conducted. We will undertake a scoping review to identify and describe this literature, and as a first step toward a future mixed-methods evidence synthesis.

Review objectives

The primary objective of this scoping review is to comprehensively identify and map the empirical evidence on unintended consequences (beneficial and harmful) related to measures affecting human travel across national borders, designed to control and/or mitigate the SARS-CoV-2/COVID-19 pandemic. We aim to generate a broad descriptive overview of this evidence, both to develop a preliminary understanding of the array of unintended and adverse effects of travel measures, as well as the study designs and methodological approaches that have been used to assess them. The secondary objective is to inform the conduct of a possible future qualitative or mixed-methods evidence synthesis of (a subset of) this literature.

Methods

The review protocol will be registered with the Open Science Framework. We will follow established methods for the conduct of scoping reviews (Arksey & O’Malley, 2005). Furthermore, we will pilot the procedures for each stage, conduct regular team meetings and keep a list of rolling questions that will be updated continuously. The final review will be reported in compliance with the PRISMA extension for scoping reviews (Tricco et al., 2018).

Criteria for considering studies for this review

Study type

Identification of unintended consequences of complex interventions demands attention to a variety of evidence, both quantitative and qualitative. We will therefore include studies of any design, as long as they provide empirical evidence of some kind on unintended consequences of travel measures. Broadly speaking, we will consider quantitative studies, qualitative studies, and mixed-methods studies. In the following we elaborate on each of these categories.

Related to quantitative evidence, it has long been recognized that for complex interventions, such as those in public health, systematic reviews should consider study designs beyond randomized controlled trials, such as non-randomized studies of interventions (NRSI) (Reeves et al. 2013). Additionally, in the context of a global pandemic, even these types of studies may not be feasible, and modelling studies may play an important role in assessing the effects of such interventions. Thus, we will include any study that provides a quantitative assessment of at least one unintended consequence or adverse effect of one or several travel-related control measures related to SARS-CoV-2/Covid-19. These study designs include, but are not limited to:

- Experimental and quasi-experimental studies (e.g., randomized controlled trials (RCTs), interrupted time series studies (ITS), controlled before-after (CBA) studies, difference-in-difference (DiD) studies, instrumental variable (IV) studies, regression discontinuity (RD) studies)
- Observational studies (e.g., cohort studies, case-control studies)
- Mathematical modelling studies (e.g., Bayesian hierarchical models, economic modelling studies)

There is a growing interest in including qualitative evidence in systematic reviews for their potential contribution to aspects of decision making not captured by quantitative studies (Noyes et al., 2020). Furthermore, qualitative research might be able to identify evidence of unintended consequences that is either not reported or difficult to detect in quantitative studies (Thomas & Harden, 2008). We adopt the definition used in the most recent edition of the Cochrane Handbook’s guidance on the use of qualitative evidence in systematic reviews: “[a] qualitative study is a research study that uses a qualitative method of data collection *and* analysis.” (Noyes et al., 2020; emphasis on original). Methods of qualitative data collection include, for example, interviews, focus groups, documents and (participant) observation. Qualitative data analysis methods include, but are not limited to, various forms of thematic analysis, both highly inductive (e.g., grounded theory) and more deductive (e.g., framework analysis), phenomenological approaches, discourse analysis, hermeneutics, ethnography and narrative approaches.

We will also consider mixed-methods studies, which combine both qualitative and quantitative research components, e.g., by collecting, analyzing and interpreting both qualitative and quantitative data (Schoonenboom & Johnson, 2017). Mixed-methods studies can vary in their “(a) level of mixing (partially mixed versus fully mixed); (b) time orientation (concurrent versus sequential); and (c) emphasis of approaches (equal status versus dominant status)” (Leech & Onwuegbuzie, 2009, p. 268).

The examples listed in the categories above are meant to be indicative rather than comprehensive. This is likely not an exhaustive list of all the possible relevant studies, and – independent of the actual study design – studies use a variety of labels. We will consider all studies providing a quantitative measure or qualitative assessment of the unintended consequences of travel-related control measures, regardless of whether they are indicated by one of these labels. We will consider studies published in peer-reviewed academic journals, as well as those published on preprint servers.

We will exclude gray literature (e.g., government reports), all non-empirical studies (e.g., commentaries, editorials, literature reviews not reporting primary empirical data), and conference abstracts. Systematic reviews will also be excluded, although any identified systematic reviews with a focus that overlaps with that of this scoping review will be retained and their lists of included studies scanned for relevant studies.

Language

Where possible, we will consider studies published in all languages. Within the review team, we are able to address studies in Armenian, English, French, German, Italian, Russian and Spanish. For studies in other languages, we will, where possible, use existing networks to support us with screening and/or translation.

Population

We will be very inclusive regarding the population and will consider studies in all populations that are personally or professionally impacted by the enactment, implementation and/or relaxation of travel-related control measures at individual or organizational level. Groups that are directly impacted by these measures include, for example, travelers and travel staff (e.g., crew members), government decision makers, staff implementing and monitoring the measures, and the travel industry (e.g., airlines, airports, cruise operators, railway companies, hotels). Other populations impacted less directly and outside of the travel setting include families and friends of affected persons, the work environment of affected persons, other industries reliant on travel (e.g., bars, restaurants) and the general population.

To be eligible, studies must focus on SARS-CoV-2/COVID-19.

We will exclude studies concerned with the consequences of travel-related control measures regarding other infectious diseases with epidemic/pandemic potential (e.g., influenza, SARS-CoV-1/SARS, MERS-CoV/MERS, Ebola, viral meningitis).

Intervention

To be eligible for inclusion, studies must investigate travel-related control measures affecting human travel across national borders during the SARS-CoV-2/COVID-19 pandemic. We will consider both introduction and implementation, as well as relaxation and de-implementation of the following measures:

- Border closure (i.e., completely stopping cross-border travel)
- International travel restrictions, which aim to
  - Deny entry or exit, or both, on the basis of nationality, travel history, point of origin, health status or other characteristics
  - Partially reduce cross-border travel via any or all of land, air and sea
  - Require or refuse visa on the basis of nationality, travel history, health status or other characteristics
- Entry or exit screening at national borders, or both
  - Temperature measurement (e.g., thermography)
  - Health questionnaire or declaration forms (e.g., symptoms, travel history, contact history)
  - Physical examination
  - Testing for current or past infection
  - Passive observation
- Quarantine of travelers crossing national borders for any period of time, regardless of where this took place (e.g., quarantine centers, hotels, home) and whether or how this was enforced
- Any combination of the above measures

We will exclude the following types of interventions:

- Combinations of the above-mentioned travel-related control measures with other public health interventions where the consequences of travel-related control measures cannot be disentangled from the consequences of those other measures.
- All interventions not directly related to travel, including a range of containment and mitigation measures (e.g., community-based quarantine, symptom screening in retail stores).
- All interventions related to the movement of animals or goods.
- All interventions concerned with human travel within national borders.
- Travel warnings or travel advice issued by the World Health Organization or national governments.

Outcome

To be eligible for inclusion, studies must report on at least one unintended consequence of travel-related control measures (Figure 1). In the frame of this review, we define unintended consequences as either positive or negative effects that are not part of how the intervention is expected to achieve its desired outcomes. This definition therefore excludes both the desired outcome of the intervention, as well as any intermediate outcomes in its hypothesized causal chain. Unintended outcomes might be predictable (desirable or undesirable) consequences that were contemplated when designing and/or implementing the intervention, but also less predictable, more surprising consequences. Whether a consequence is “unintended” will be judged by the review authors – it does not need to be explicitly identified in this way by the primary study authors. Eligible outcomes include, but are not limited to, the following categories, adapted from a framework to facilitate evidence-based decision-making during the SARS-CoV-2/COVID-19 pandemic (Stratil et al., 2020).

- Implications for quality of life, social well-being, and mental health (e.g., experience of loneliness, cohesion of families or non-family communities, depression, anxiety)
- Implications for physical health, health behavior, health risks, and healthcare beyond COVID-19 (e.g., willingness to seek health care for other reasons than COVID-19, domestic violence)
- Equity, equality and the fair distribution of benefits and burdens (e.g., stigmatization, discrimination)
- Social and environmental implications (e.g., educational effects, household income, greenhouse gas emissions, noise level)
- Economic implications (e.g., tourism industry losses, visitor accommodation and repatriation costs)

This review will therefore exclude studies that look exclusively at the targeted/intended outcomes of travel-related control measures, namely, outcomes related to SARS-CoV-2 transmission, and those related to COVID-19 epidemic progression and/or severity.

Identification of relevant studies

Given the broad nature of both the assessed measures as well as the potential unintended consequences and adverse effects, we will search a range of databases with the aim to capture at least one database across each of the disciplines medicine, psychology, sociology, economy and ecology:

- Ovid MEDLINE(R) (1946 to present)
- Ovid Embase (1996 to present)
- Business Source Complete
- GreenFILE
- APA PsycINFO
- Web of Science (Social Sciences Citation Index)
- Web of Science (Science Citation Index Expanded)

Our search strategy is structured around two main search concepts: (1) SARS-CoV-2/COVID-19 and (2) travel-related control measures. We adapted the search strategy of Burns et al. (2020) for our review. The initial search strategy was developed for Ovid MEDLINE(R) (Appendix 1) and will be adapted for the other databases. We will conduct the searches in English but will aim to include studies published in any language (see “Language” above). Results will be limited to publication year 2020 to present.

We will additionally search the following COVID-19-specific databases:

- Cochrane’s COVID-19 Study Register (<https://covid-19.cochrane.org/>): this includes published articles, trial registry records and preprints
- WHO COVID-19 Global literature on coronavirus disease (<https://search.bvsalud.org/global-literature-on-novel-coronavirus-2019-ncov/>): this contains primarily research (published and/or pre-publication) journal articles

We will also conduct backward citation searches of all relevant systematic reviews and included studies identified through the searches. We will conduct these searches in Web of Science and/or Microsoft Academic.

Data collection and analysis

Selection of studies

After de-duplication, titles and abstracts will be screened in duplicate, with a discussion of all unclear cases between the two reviewers or within the review team. For all studies deemed potentially relevant or unclear at the title/abstract screening stage, two reviewers will then screen the full text in duplicate. Any discrepancies will be discussed by the two screening review authors, and any unclear cases will be discussed with a third review author and/or the larger review team. At this stage, a final decision regarding inclusion will be made, and reasons for exclusion will be documented.

We will use EndNote to manage collection and de-duplication of records. For title and abstract screening, we will use the web-based application Rayyan (https://rayyan.qcri.org/welcome). For full text screening we will use Microsoft Excel.

For both the title/abstract and full text screening stages, we will develop screening guidance forms to ensure that all reviewers screen similarly and consistently. We will discuss inconsistencies and challenges encountered within the review team, after having screened approximately 50 titles/abstracts and approximately 5 full texts and will then refine the screening guidance. We will additionally collect and clarify all uncertainties in screening on a rolling basis. These will be discussed in regular online meetings to ensure consistency in screening across multiple reviewers and to address any questions and comments.

Data extraction and management

One review author will extract and chart study characteristics and data from all included studies using bespoke data extraction forms in Microsoft Excel. The forms contain different categories that comprise a priori defined aspects, as well as inductive categories that we will develop as any new concepts arise. A priori categories include: study information (e.g., publication year); study characteristics (e.g., study type); setting and context (e.g., supranational or national); population (e.g., age group); interventions (e.g., closure of national borders to entry/exit) and outcomes (e.g. outcome category). The extraction forms (Appendix 2) will be pilot-tested by the review team on at least one study per type of study before being discussed and revised as needed.

Mapping of included studies

Based on the a priori and inductively defined categories, we will collate, summarize and report the extracted data narratively, graphically, or in tabular form. Types of interventions, outcomes of interest and study designs will be presented in clusters. Findings will be summarized in a graphical evidence map, including types and combinations of interventions on the y axis and outcome categories on the x axis. The cells of the map – each representing a specific intervention-outcome category combination – will be populated by information describing the number and types of studies, if any, that have examined that intervention-outcome pair. All data presented in the tables, text and graphics will be double-checked by a second reviewer. Furthermore, we aim to revise our a priori logic model by integrating currently missing or inadequately defined categories or concepts.

Acknowledgements

We would like to thank Robin Featherstone (Information Specialist, Cochrane Editorial and Methods Department) for her valuable advice on our search strategy.

References

Arksey H & O'Malley L. Scoping studies: towards a methodological framework. *Int J Soc Res*. 2005;8(1):19-32.

Bashir MF, Benjiang M, & Shahzad L. A brief review of socio-economic and environmental impact of Covid-19. *Air Qual Atmos Health*. 2020;13:1403-1409.

Brooks SK, Webster RK, Smith LE, et al. The psychological impact of quarantine and how to reduce it: rapid review of the evidence. *Lancet*. 2020;395(10227):912-920.

Burns J, Movsisyan A, Stratil JM, et al. Travel-related control measures to contain the COVID-19 pandemic: a rapid review. *Cochrane Database of Syst Rev*. 2020;9:CD013717.

Glover RE, van Schalkwyk MC, Akl EA, et al. A framework for identifying and mitigating the equity harms of COVID-19 policy interventions. *J Clin Epidemiol*. 2020;128:35-48.

Kneale D, Thomas J, & Harris K. Developing and Optimising the Use of Logic Models in Systematic Reviews: Exploring Practice and Good Practice in the Use of Programme Theory in Reviews*. PLoS ONE*. 2015;10(11):e0142187.

Lal P, Kumar A, Kumar S, et al. The dark cloud with a silver lining: Assessing the impact of the SARS COVID-19 pandemic on the global environment. *Sci Total Environ*. 2020;732:139297.

Leech NL & Onwuegbuzie AJ. A typology of mixed methods research designs. *Qual Quant*. 2009;43:265-275.

Liew J. Spread of Anti-Asian Racism: Prevention and Critical Race Analysis in Pandemic Planning. In: Flood CM, MacDonnell V, & Philpott J (Eds.). Vulnerable: The law, policy and ethics of COVID-19 (pp. 393-406). 2020. Ottawa: University of Ottawa Press.

Looi, MK. Covid-19: Is a second wave hitting Europe? *BMJ*. 2020;371:m4113.

Nicola M, Alsafi Z, Sohrabi C, et al. The socio-economic implications of the coronavirus pandemic (COVID-19): A review. *Int J Surg*. 2020;78:185-193.

Noyes J, Booth A, Cargo M, et al. Chapter 21: Qualitative evidence. In: Higgins JPT, Thomas J, Chandler J, et al. (editors). Cochrane Handbook for Systematic Reviews of Interventions version 6.1 (updated September 2020). Cochrane, 2020. Available from www.training.cochrane.org/handbook.

Reeves BC, Higgins JPT, Ramsay C, et al. An introduction to methodological issues when including non-randomised studies in systematic reviews on the effects of interventions. *Res Syn Meth*. 2013;4:1-11.

Rehfuess EA, Booth A, Brereton L, et al. Towards a taxonomy of logic models in systematic reviews and health technology assessments: a priori, staged and iterative approaches. *Res Syn Meth*. 2018 Mar; 9(1):13-24.

Rohwer A, Pfadenhauer LM, Burns J, et al. Logic models help make sense of complexity in systematic reviews and health technology assessments*. J Clin Epidemiol*. 2017;83:37-47.

Schoonenboom J & Johnson RB. How to Construct a Mixed Methods Research Design. *Kolner Z Soz Sozpsychol.* 2017;69(Suppl 2):107-131.

Stratil JM, Voss M, & Arnold L. WICID framework Version 1.0: Criteria and considerations to guide evidence-informed decision-making on non-pharmacological interventions targeting COVID-19. *BMJ Glob Health.* 2020;5e003699.

Thomas J & Harden A. Methods for the thematic synthesis of qualitative research in systematic reviews. *BMC Med Res Methodol*. 2008;8:45.

Tricco AC, Lillie E, Zarin W, et al. PRISMA extension for scoping reviews (PRISMA-ScR): checklist and explanation. *Ann Intern Med*. 2018;169(7):467-473.

UN. COVID-19 and Human rights – we are all in this together. April 2020. URL: https://www.un.org/victimsofterrorism/sites/www.un.org.victimsofterrorism/files/un_-_human_rights_and_covid_april_2020.pdf. (Last accessed: November 14^th^, 2020)

UNWTO. COVID-19 related travel restrictions – a global review for tourism. Eighth report as of 2 December 2020. URL: https://webunwto.s3.eu-west-1.amazonaws.com/s3fs-public/2020-12/201202-Travel-Restrictions.pdf. (Last accessed: December 20^th^, 2020)

WHO. Novel Coronavirus (2019-nCoV) Situation Report – 51. 2020. World Health Organization. URL: https://apps.who.int/iris/handle/10665/331475 (Last accessed: November 14^th,^ 2020)

Appendices

Protocol Appendix 1. Search Strategy

**Database**: Ovid MEDLINE(R) and Epub Ahead of Print, In-Process & Other Non-Indexed Citations, Daily and Versions(R) 1946 to Dec 12, 2020

Strategy:

1 exp Coronavirus/ (45017)

2 Coronavirus Infections/ (44000)

3 COVID-19.rs. (39029)

4 severe acute respiratory syndrome coronavirus 2.os. (33023)

5 (2019 nCoV or 2019nCoV or 2019-novel CoV).ti,ab,kf. (805)

6 (Coronavir* or corona virus* or Severe Acute Respiratory Syndrome* or SARS*).ti,ab,kf. (37396)

7 COVID 19.mp. (39643)

8 (COVID19 or COVID 2019).ti,ab,kf. (424)

9 (nCov 2019 or nCov 19).ti,ab,kf. (52)

10 or/1-9 [Set 1: Coronaviruses] (60176)

11 Air Travel/ (400)

12 Travel/ (25539)

13 (border? adj3 (clos* or restrict* or control* or measure?)).ti,ab,kf. (1028)

14 ((isolat* or quarantin*) adj6 (exposed or suspected or travel* or airport? or border?)).ti,ab,kf. (7354)

15 ((mobility or movement*) adj2 (reduc* or restrict*)).ti,ab,kf. (8165)

16 ((questionnaire* or screen* or surveil*) adj4 (traveller? or traveler? or entr* or exit or border? or airport?)).ti,ab,kf. (1755)

17 (travel* or border?).ti. (23039)

18 (travel adj4 (measure? or intervention? or NPI?)).ti,ab,kf. (391)

19 (travel* adj3 (restrict* or reduc* or control* or limit* or lockdown? or ban*)).ti,ab,kf. (1477)

20 visa?.ti,ab,kf. (1697)

21 or/11-20 [Set 2: Travel measures] (58946)

22 and/10,21 [Sets 1 & 2] (1211)

23 limit 22 to "humans only (removes records about animals)" (1190)

24 remove duplicates from 23 (1188)

25 limit 24 to yr = “2020 -2021” (**777**)

Protocol Appendix 2. Categories in the data extraction forms

**NOTE:** not all aspects are relevant for the different study designs considered in this review. Some aspects for example will just apply to modelling studies, others are just relevant for qualitative studies.

Study information

- Study ID
- Study title
- Study source (i.e., journal, report, pre-print publication)
- Date of submission
- Date of publication

Study characteristics

- Study type (e.g., experimental, quasi-experimental, observational, modelling, qualitative, mixed-methods)
- Verbal summary of study type as reported by study author(s) (e.g., stochastic discrete event simulation model, interrupted time series study, explanatory sequential mixed study)
- Comments

Setting and context

- Level of intervention (i.e., supranational level, national level)
- Country/countries in which travel-related control measure is implemented
- Country/countries restricted by travel-related control measure
- Mode of travel (e.g., land, air, sea, any combination of these)
- Comments

Population

- Short description of the targeted population, i.e., the population affected by the travel-related control measure (e.g., international travelers arriving between 27 April and 22 June 2020
- Short description of the studied population, i.e., the population in which the outcome is assessed (e.g., families of international travelers arriving between 27 April and 22 June 2020)
- Age group
- Comments

Intervention

- Broad measure category (i.e., closure of national borders to entry/exit; international travel restrictions; entry/exit screening at national borders; quarantine or isolation of travelers crossing national borders)
- Verbal summary of specific travel-related control measure(s) (e.g., all arriving passengers were provided with a symptom questionnaire)
- Date(s) of implementation of the travel-related control measure(s)
- Duration of the intervention
- Any reported exceptions to the measure (e.g., certain individuals being excluded or fast-tracked in airport screening because of nationality, occupation, country of origin)
- Representation of the intervention in the model (e.g., 100% travel reduction)
- Non-travel-related co-interventions (assessed in the study)
- Comments

Outcomes (repeated for each outcome):

- Outcome category
- Description of outcome
- Length of follow-up
- Comments

Conflicts of interest statements and funding sources

## Appendix 2. Preferred Reporting Items for Systematic reviews and Meta-Analyses extension for Scoping Reviews (PRISMA-ScR) Checklist

| **SECTION** | **ITEM** | **PRISMA-ScR CHECKLIST ITEM** | **REPORTED ON PAGE #** |
| --- | --- | --- | --- |
| **TITLE** | | | |
| Title | 1 | Identify the report as a scoping review. | 1 |
| **ABSTRACT** | | | |
| Structured summary | 2 | Provide a structured summary that includes (as applicable): background, objectives, eligibility criteria, sources of evidence, charting methods, results, and conclusions that relate to the review questions and objectives. | 2-3 |
| **INTRODUCTION** | | | |
| Rationale | 3 | Describe the rationale for the review in the context of what is already known. Explain why the review questions/objectives lend themselves to a scoping review approach. | 4-5 |
| Objectives | 4 | Provide an explicit statement of the questions and objectives being addressed with reference to their key elements (e.g., population or participants, concepts, and context) or other relevant key elements used to conceptualize the review questions and/or objectives. | 6 |
| **METHODS** | | | |
| Protocol and registration | 5 | Indicate whether a review protocol exists; state if and where it can be accessed (e.g., a Web address); and if available, provide registration information, including the registration number. | 6 |
| Eligibility criteria | 6 | Specify characteristics of the sources of evidence used as eligibility criteria (e.g., years considered, language, and publication status), and provide a rationale. | 7, Tab. 1, App. 1 |
| Information sources* | 7 | Describe all information sources in the search (e.g., databases with dates of coverage and contact with authors to identify additional sources), as well as the date the most recent search was executed. | 7 |
| Search | 8 | Present the full electronic search strategy for at least 1 database, including any limits used, such that it could be repeated. | App. 3 |
| Selection of sources of evidence† | 9 | State the process for selecting sources of evidence (i.e., screening and eligibility) included in the scoping review. | 8 |
| Data charting process‡ | 10 | Describe the methods of charting data from the included sources of evidence (e.g., calibrated forms or forms that have been tested by the team before their use, and whether data charting was done independently or in duplicate) and any processes for obtaining and confirming data from investigators. | 8, App. 5 |
| Data items | 11 | List and define all variables for which data were sought and any assumptions and simplifications made. | App. 1, App. 5 |
| Critical appraisal of individual sources of evidence§ | 12 | If done, provide a rationale for conducting a critical appraisal of included sources of evidence; describe the methods used and how this information was used in any data synthesis (if appropriate). | NA |
| Synthesis of results | 13 | Describe the methods of handling and summarizing the data that were charted. | 8-9 |
| **RESULTS** | | | |
| Selection of sources of evidence | 14 | Give numbers of sources of evidence screened, assessed for eligibility, and included in the review, with reasons for exclusions at each stage, ideally using a flow diagram. | 9, Fig. 2, App. 4, App. 6 |
| Characteristics of sources of evidence | 15 | For each source of evidence, present characteristics for which data were charted and provide the citations. | Tab. 2 |
| Critical appraisal within sources of evidence | 16 | If done, present data on critical appraisal of included sources of evidence (see item 12). | NA |
| Results of individual sources of evidence | 17 | For each included source of evidence, present the relevant data that were charted that relate to the review questions and objectives. | Tab. 2, 12-16 |
| Synthesis of results | 18 | Summarize and/or present the charting results as they relate to the review questions and objectives. | 9-12, Fig. 3 |
| **DISCUSSION** | | | |
| Summary of evidence | 19 | Summarize the main results (including an overview of concepts, themes, and types of evidence available), link to the review questions and objectives, and consider the relevance to key groups. | 16-17 |
| Limitations | 20 | Discuss the limitations of the scoping review process. | 17-19 |
| Conclusions | 21 | Provide a general interpretation of the results with respect to the review questions and objectives, as well as potential implications and/or next steps. | 19 |
| **FUNDING** | | | |
| Funding | 22 | Describe sources of funding for the included sources of evidence, as well as sources of funding for the scoping review. Describe the role of the funders of the scoping review. | 20, Tab. 2 |

## Appendix 3. Search strategies and results

**Database**: Ovid MEDLINE(R) ALL 1946 to Present

**Date search conducted**: 12 December 2020

**Strategy**:

1 exp Coronavirus/ (45017)

2 Coronavirus Infections/ (44000)

3 COVID-19.rs. (39029)

4 severe acute respiratory syndrome coronavirus 2.os. (33023)

5 (2019 nCoV or 2019nCoV or 2019-novel CoV).ti,ab,kf. (805)

6 (Coronavir* or corona virus* or Severe Acute Respiratory Syndrome* or SARS*).ti,ab,kf. (37396)

7 COVID 19.mp. (39643)

8 (COVID19 or COVID 2019).ti,ab,kf. (424)

9 (nCov 2019 or nCov 19).ti,ab,kf. (52)

10 or/1-9 [Set 1: Coronaviruses] (60176)

11 Air Travel/ (400)

12 Travel/ (25539)

13 (border? adj3 (clos* or restrict* or control* or measure?)).ti,ab,kf. (1028)

14 ((isolat* or quarantin*) adj6 (exposed or suspected or travel* or airport? or border?)).ti,ab,kf. (7354)

15 ((mobility or movement*) adj2 (reduc* or restrict*)).ti,ab,kf. (8165)

16 ((questionnaire* or screen* or surveil*) adj4 (traveller? or traveler? or entr* or exit or border? or airport?)).ti,ab,kf. (1755)

17 (travel* or border?).ti. (23039)

18 (travel adj4 (measure? or intervention? or NPI?)).ti,ab,kf. (391)

19 (travel* adj3 (restrict* or reduc* or control* or limit* or lockdown? or ban*)).ti,ab,kf. (1477)

20 visa?.ti,ab,kf. (1697)

21 or/11-20 [Set 2: Travel measures] (58946)

22 and/10,21 [Sets 1 & 2] (1211)

23 limit 22 to "humans only (removes records about animals)" (1190)

24 remove duplicates from 23 (1188)

25 limit 24 to yr = “2020 -2021” (**777**)

**Database**: Ovid Embase 1988 to Present

**Date search conducted**: 12 December 2020

**Strategy**:

1 exp coronaviridae/ (23098)

2 exp coronavirus infection/ (24168)

3 (2019 nCoV or 2019nCoV or 2019-novel CoV).ti,ab,kw. (1347)

4 (Coronavir* or corona virus* or Severe Acute Respiratory Syndrome* or SARS*).ti,ab,kw. (61360)

5 COVID 19.af. (69877)

6 (COVID19 or COVID 2019).ti,ab,kw. (1128)

7 (nCov 2019 or nCov 19).ti,ab,kw. (68)
8 or/1-7 [Set 1: Coronaviruses] (105080)
9 air transportation/ (108)
10 aviation/ (6171)
11 travel/ (46411)
12 (border? adj3 (clos* or restrict* or control* or measure?)).ti,ab,kw. (1296)
13 ((isolat* or quarantin*) adj6 (exposed or suspected or travel* or airport? or border?)).ti,ab,kw. (8744)
14 ((mobility or movement*) adj2 (reduc* or restrict*)).ti,ab,kw. (12668)
15 ((questionnaire* or screen* or surveil*) adj4 (traveller? or traveler? or entr* or exit or border? or airport?)).ti,ab,kw. (2715)

16 (travel* or border?).ti. (26008)
17 (travel adj4 (measure? or intervention? or NPI?)).ti,ab,kw. (573)
18 (travel* adj3 (restrict* or reduc* or control* or limit* or lockdown? or ban*)).ti,ab,kw. (2361)
19 visa?.ti,ab,kw. (2471)
20 or/ 9-19 [Set 2: Travel measures] (94398)

21 and/8,20 [Sets 1 & 2] (2511)

22 (animal experiment/ or exp animal/) not exp human/ (3973874)

23 21 not 22 (2485)

24 conference abstract.pt. (3915310)

25 23 not 24 (2340)

26 remove duplicates from 25 (2305)

27 limit 26 to yr="2020 – 2021" (**1650**)

**Database**: Business Source Complete 2002 to Present

**Date search conducted**: 12 December 2020

**Strategy**:

S1 DE "COVID-19" (34861)

S2 DE "COVID-19 pandemic" (52166)

S3 DE "SARS-CoV-2" (2726)

S4 TI (2019-ncov OR 2019ncov OR 2019 novel coronavirus) OR AB (2019-ncov OR 2019ncov OR 2019 novel coronavirus) OR KW (2019-ncov OR 2019ncov OR 2019 novel coronavirus) (499)

S5 TI (coronavir* OR corona virus* OR severe acute respiratory syndrome* OR SARS*) OR AB (coronavir* OR corona virus* OR severe acute respiratory syndrome* OR SARS*) OR KW (coronavir* OR corona virus* OR severe acute respiratory syndrome* OR SARS*) (73986)

S6 TI (covid 19 OR covid19 OR covid 2019) OR AB (covid 19 OR covid19 OR covid 2019) OR KW (covid 19 OR covid19 OR covid 2019) (685)

S7 TI (ncov-19 OR ncov-2019) OR AB (ncov-19 OR ncov-2019) OR KW (ncov-19 OR ncov-2019) (9)

S8 S1 OR S2 OR S3 OR S4 OR S5 OR S6 OR S7 (128034)

S9 DE "travel" (42078)

S10 TI (border#? N3 (clos* OR restrict* OR control* OR measure#?)) OR AB (border#? N3 (clos* OR restrict* OR control* OR measure#?)) OR KW (border#? N3 (clos* OR restrict* OR control* OR measure#?)) (661)

S11 TI ((isolat* OR quarantin*) N6 (exposed OR suspected OR travel* OR airport#? OR border#?)) OR AB ((isolat* OR quarantin*) N6 (exposed OR suspected OR travel* OR airport#? OR border#?)) OR KW (isolat* OR quarantin*) N6 (exposed OR suspected OR travel* OR airport#? OR border#?)) (495)

S12 TI ((mobility OR movement*) N2 (reduc* OR restrict*)) OR AB ((mobility OR movement*) N2 (reduc* OR restrict*)) OR KW ((mobility OR movement*) N2 (reduc* OR restrict*)) (1792)

S13 TI ((questionnaire* OR screen* OR surveil*) N4 (traveller#? OR traveler#? OR entr* OR exit OR border#? OR airport#?)) OR AB ((questionnaire* OR screen* OR surveil*) N4 (traveller#? OR traveler#? OR entr* OR exit OR border#? OR airport#?)) OR KW ((questionnaire* OR screen* OR surveil*) N4 (traveller#? OR traveler#? OR entr* OR exit OR border#? OR airport#?)) (1115)

S14 TI (travel* OR border#?) (55958)

S15 TI (travel N4 (measure#? OR intervention#? or NPI#?)) OR AB (travel N4 (measure#? OR intervention#? or NPI#?)) OR KW (travel N4 (measure#? OR intervention#? or NPI#?)) (528)

S16 TI (travel* N3 (restrict* OR reduc* OR control* OR limit* OR lockdown#? or ban*)) OR AB (travel* N3 (restrict* OR reduc* OR control* OR limit* OR lockdown#? or ban*)) OR KW (travel* N3 (restrict* OR reduc* OR control* OR limit* OR lockdown#? or ban*)) (8239)

S17 TI visa#? OR AB visa#? OR KW visa#? (9730)

S18 S9 OR S10 OR S11 OR S12 OR S13 OR S14 OR S15 OR S16 OR S17 (108303)

S19 S8 AND S18 (3205)

S20 S8 AND S18 - Publication Date: 20200101 – 20211231 (**2960**)

**Database**: GreenFILE 1999 to Present

**Date search conducted**: 12 December 2020

**Strategy**:

S1 TI (2019-ncov OR 2019ncov OR 2019 novel coronavirus) OR AB (2019-ncov OR 2019ncov OR 2019 novel coronavirus) OR KW (2019-ncov OR 2019ncov OR 2019 novel coronavirus) (11)

S2 TI (coronavir* OR corona virus* OR severe acute respiratory syndrome* OR SARS*) OR AB (coronavir* OR corona virus* OR severe acute respiratory syndrome* OR SARS*) OR KW (coronavir* OR corona virus* OR severe acute respiratory syndrome* OR SARS*) (722)

S3 TI (covid 19 OR covid19 OR covid 2019) OR AB (covid 19 OR covid19 OR covid 2019) OR KW (covid 19 OR covid19 OR covid 2019) (24)

S4 TI (ncov-19 OR ncov-2019) OR AB (ncov-19 OR ncov-2019) OR KW (ncov-19 OR ncov-2019) (0)

S5 S1 OR S2 OR S3 OR S4 (737)

S6 DE "AIR travel & the environment" (30)

S7 DE "TRANSPORTATION & the environment" (380)

S8 DE "MOTOR vehicles & the environment" (162)

S9 DE "VEHICLES & the environment" (231)

S10 TI (border#? N3 (clos* OR restrict* OR control* OR measure#?)) OR AB (border#? N3 (clos* OR restrict* OR control* OR measure#?)) OR KW (border#? N3 (clos* OR restrict* OR control* OR measure#?)) (27)

S11 TI ((isolat* OR quarantin*) N6 (exposed OR suspected OR travel* OR airport#? OR border#?)) OR AB ((isolat* OR quarantin*) N6 (exposed OR suspected OR travel* OR airport#? OR border#?)) OR KW ((isolat* OR quarantin*) N6 (exposed OR suspected OR travel* OR airport#? OR border#?)) (138)

S12 TI ((mobility OR movement*) N2 (reduc* OR restrict*)) OR AB ((mobility OR movement*) N2 (reduc* OR restrict*)) OR KW ((mobility OR movement*) N2 (reduc* OR restrict*)) (656)

S13 TI ((questionnaire* OR screen* OR surveil*) N4 (traveller#? OR traveler#? OR entr* OR exit OR border#? OR airport#?)) OR AB ((questionnaire* OR screen* OR surveil*) N4 (traveller#? OR traveler#? OR entr* OR exit OR border#? OR airport#?)) OR KW ((questionnaire* OR screen* OR surveil*) N4 (traveller#? OR traveler#? OR entr* OR exit OR border#? OR airport#?)) (24)

S14 TI (travel* OR border?) (1565)

S15 TI (travel N4 (measure#? OR intervention#? or NPI#?)) OR AB (travel N4 (measure#? OR intervention#? or NPI#?)) OR KW (travel N4 (measure#? OR intervention#? or NPI#?)) (30)

S16 TI (travel* N3 (restrict* OR reduc* OR control* OR limit* OR lockdown#? or ban*)) OR AB (travel* N3 (restrict* OR reduc* OR control* OR limit* OR lockdown#? or ban*)) OR KW (travel* N3 (restrict* OR reduc* OR control* OR limit* OR lockdown#? or ban*)) (252)

S17 TI visa#? OR AB visa#? OR KW visa#? (423)

S18 S6 OR S7 OR S8 OR S9 OR S10 OR S11 OR S12 OR S13 OR S14 OR S15 OR S16 OR S17 (3818)

S19 S5 AND S18 (19)

S20 S5 AND S18 - Publication Date: 20200101 – 20211231 (**19**)

**Database**: APA PsycINFO 1992 to Present

**Date search conducted**: 12 December 2020

**Strategy**:

S1 DE "Coronavirus" (1485)

S2 DE "Severe Acute Respiratory Syndrome" (269)

S3 TI (2019-ncov OR 2019ncov OR 2019 novel coronavirus) OR AB (2019-ncov OR 2019ncov OR 2019 novel coronavirus) OR KW (2019-ncov OR 2019ncov OR 2019 novel coronavirus) (93)

S4 TI (coronavir* OR corona virus* OR severe acute respiratory syndrome* OR SARS*) OR AB (coronavir* OR corona virus* OR severe acute respiratory syndrome* OR SARS*) OR KW (coronavir* OR corona virus* OR severe acute respiratory syndrome* OR SARS*) (1850)

S5 TI (covid 19 OR covid19 OR covid 2019) OR AB (covid 19 OR covid19 OR covid 2019) OR KW (covid 19 OR covid19 OR covid 2019) (146)

S6 TI (ncov-19 OR ncov-2019) OR AB (ncov-19 OR ncov-2019) OR KW (ncov-19 OR ncov-2019) (0)

S7 S1 OR S2 OR S3 OR S4 OR S5 OR S6 (2651)

S8 DE "Traveling" (3009)

S9 DE "Commuting (Travel)" (587)

S10 TI (border#? N3 (clos* OR restrict* OR control* OR measure#?)) OR AB (border#? N3 (clos* OR restrict* OR control* OR measure#?)) OR KW (border#? N3 (clos* OR restrict* OR control* OR measure#?)) (81)

S11 TI ((isolat* OR quarantin*) N6 (exposed OR suspected OR travel* OR airport#? OR border#?)) OR AB ((isolat* OR quarantin*) N6 (exposed OR suspected OR travel* OR airport#? OR border#?)) OR KW ((isolat* OR quarantin*) N6 (exposed OR suspected OR travel* OR airport#? OR border#?)) (426)

S12 TI ((mobility OR movement*) N2 (reduc* OR restrict*)) OR AB ((mobility OR movement*) N2 (reduc* OR restrict*)) OR KW ((mobility OR movement*) N2 (reduc* OR restrict*)) (2218)

S13 TI ((questionnaire* OR screen* OR surveil*) N4 (traveller#? OR traveler#? OR entr* OR exit OR border#? OR airport#?)) OR AB ((questionnaire* OR screen* OR surveil*) N4 (traveller#? OR traveler#? OR entr* OR exit OR border#? OR airport#?)) OR KW ((questionnaire* OR screen* OR surveil*) N4 (traveller#? OR traveler#? OR entr* OR exit OR border#? OR airport#?)) (692)

S14 TI (travel* OR border#?) (3902)

S15 TI (travel N4 (measure#? OR intervention#? or NPI#?)) OR AB (travel N4 (measure#? OR intervention#? or NPI#?)) OR KW (travel N4 (measure#? OR intervention#? or NPI#?)) (157)

S16 TI (travel* N3 (restrict* OR reduc* OR control* OR limit* OR lockdown#? or ban*)) OR AB (travel* N3 (restrict* OR reduc* OR control* OR limit* OR lockdown#? or ban*)) OR KW (travel* N3 (restrict* OR reduc* OR control* OR limit* OR lockdown#? or ban*)) (587)

S17 TI visa#? OR AB visa#? OR KW visa#? (899)

S18 S8 OR S9 OR S10 OR S11 OR S12 OR S13 OR S14 OR S15 OR S16 OR S17 (10857)

S19 S7 AND S18 (65)

S20 S7 AND S18 - Publication Year: 2020 – 2021 (**54**)

**Database**: Web of Science (Social Sciences Citation Index) 1900 to Present

**Date search conducted**: 12 December 2020

**Strategy**:

# 1 TS=(coronavir*  OR coronovir*) (4001)

# 2 TS=((corona*  OR corono*) NEAR/1 (virus*  OR viral*  OR virinae*)) (151)

# 3 TS=(2019 nCoV OR 2019nCoV OR 2019-novel CoV OR Coronavir* OR corona virus* OR "Severe Acute Respiratory Syndrome" OR SARS* OR COVID 19 OR COVID19 OR COVID 2019 OR nCov 2019 OR nCov 19) (13301)

# 4 #3  OR #2  OR #1 (13302)

# 5 TS=(border$ NEAR/3 (clos*  OR restrict*  OR control*  OR measure$)) (1408)

# 6 TS=((isolat* OR quarantin*) NEAR/6 (exposed  OR suspected  OR travel*  OR airport$  OR border$)) (357)

# 7 TS=((mobility or movement*) NEAR/2 (reduc* or restrict*)) (2535)

# 8 TS=((questionnaire* OR screen*  OR surveil*) NEAR/4 (traveller$  OR traveler$  OR entr*  OR exit  OR border$  OR airport$)) (1021)

# 9 TI=(travel*  OR border$) (23218)

# 10 TS=(travel NEAR/4 (measure$  OR intervention$  OR NPI$)) (1006)

# 11 TS=(travel* NEAR/3 (restrict*  OR reduc*  OR control*  OR limit*  OR lockdown$  OR ban*)) (2200)

# 12 TS=(visa$) (1200)

# 13 #12 OR #11 OR #10 OR #9 OR #8 OR #7 OR #6 OR #5 (30745)

# 14 #13 AND #4 (338)

# 15 #13 AND #4 - Custom year range: 2020 – 2020 (**302**)

**Database**: Web of Science (Science Citation Index Expanded) 1900 to Present

**Date search conducted**: 12 December 2020

**Strategy**:

# 1 TS=(coronavir*  OR coronovir*) (35329)

# 2 TS=((corona*  OR corono*) NEAR/1 (virus*  OR viral*  OR virinae*)) (1516)

# 3 TS=(2019 nCoV OR 2019nCoV OR 2019-novel CoV OR Coronavir* OR corona virus* OR "Severe Acute Respiratory Syndrome" OR SARS* OR COVID 19 OR COVID19 OR COVID 2019 OR nCov 2019 OR nCov 19) (73082)

# 4 #3  OR #2  OR #1 (73109)

# 5 TS=(border$ NEAR/3 (clos*  OR restrict*  OR control*  OR measure$)) (2423)

# 6 TS=((isolat* OR quarantin*) NEAR/6 (exposed  OR suspected  OR travel*  OR airport$  OR border$)) (8641)

# 7 TS=((mobility or movement*) NEAR/2 (reduc* or restrict*)) (17763)

# 8 TS=((questionnaire* OR screen*  OR surveil*) NEAR/4 (traveller$  OR traveler$  OR entr*  OR exit  OR border$  OR airport$)) (2569)

# 9 TI=(travel*  OR border$) (54149)

# 10 TS=(travel NEAR/4 (measure$  OR intervention$  OR NPI$)) (2110)

# 11 TS=(travel* NEAR/3 (restrict*  OR reduc*  OR control*  OR limit*  OR lockdown$  OR ban*)) (5555)

# 12 TS=(visa$) (2374)

# 13 #12 OR #11 OR #10 OR #9 OR #8 OR #7 OR #6 OR #5 (91157)

# 14 #13 AND #4 (877)

# 15 #13 AND #4 - Custom year range: 2020 – 2020 (**662**)

**Database**: Cochrane COVID-19 Study Register

**URL**: covid-19.cochrane.org

**Date search conducted**: 12 December 2020

**Strategy**:

#1 border OR borders OR travel* OR traveling OR travelling OR traveler* OR traveller* OR airport* OR visa* (983)

#2 (questionnaire* OR screen* OR surveil*) AND (entr* OR exit) (151)

#3 1 OR 2 (**1285**)

Contents note: The Cochrane COVID-19 Study Register contains study references from PubMed, Embase.com, Cochrane Central Register of Controlled Trials (CENTRAL), ClinicalTrials.gov, WHO International Clinical Trials Registry Platform (ICTRP), medRxiv and Retraction Watch.

**Database**: WHO COVID-19 Global literature on coronavirus disease

**URL**: search.bvsalud.org/global-literature-on-novel-coronavirus-2019-ncov

**Date search conducted**: 12 December 2020

**Strategy**:

(ti:(border OR borders OR travel*)) OR (tw:(border* AND (clos* OR restrict* OR control* OR measure*))) OR (tw:((isolat* OR quarantin*) AND (travel* OR airport* OR border*))) OR (tw:((mobility OR movement*) AND (reduc* OR restrict*) AND travel*)) OR (tw:((questionnaire* OR screen* OR surveil*) AND (traveller* OR traveler* OR entry OR entries OR entrance OR exit OR border* OR airport*))) OR (tw:(travel AND (measure OR measures OR intervention OR interventions OR NPI OR NPIs))) OR (tw:(travel* AND (restrict* OR reduc* OR control* OR limit* OR lockdown* OR ban*))) OR (tw:(visa OR visas)) (**3089**)

Contents note: The WHO COVID-19 Global literature on coronavirus disease database contains primarily research (published AND/OR pre-publication) journal articles from [bibliographic databases](https://worldhealthorg-my.sharepoint.com/:w:/r/personal/garnicacarrenoj_who_int/_layouts/15/Doc.aspx?sourcedoc=%7BA34F73DC-202F-4AF4-8D84-5DA8275A352A%7D&file=WHO%20COVID-19_Sources_SearchStrategy_20210105.docx&action=default&mobileredirect=true), hand searching, and the addition of other expert-referred scientific articles.

## Appendix 4. List of reviews, commentaries and discussion papers considered for backward citation tracking

1. Abdullah WJ, Kim S. Singapore’s Responses to the COVID-19 Outbreak: A Critical Assessment. *The American Review of Public Administration*. 2020;50(6-7):770-776. [DOI: [10.1177/0275074020942454](https://doi.org/10.1177/0275074020942454)]

2. Abed Alah M, Abdeen S, & Kehyayan V. The first few cases and fatalities of Corona Virus Disease 2019 (COVID-19) in the Eastern Mediterranean Region of the World Health Organization: A rapid review. *Journal of infection and public health*, 2020;13(10): 1367–1372. [DOI: 10.1016/j.jiph.2020.06.009]

3. Acuña-Zegarra MA, Comas-García A, Hernández-Vargas E, et al. The SARS-CoV-2 epidemic outbreak: a review of plausible scenarios of containment and mitigation for Mexico. *medRxiv [Preprint]*. 2020. [DOI: 10.1101/2020.03.28.20046276]

4. Al-Qahtani AA. Severe Acute Respiratory Syndrome Coronavirus 2 (SARS-CoV-2): Emergence, history, basic and clinical aspects. *Saudi J Biol Sci*. 2020;27(10):2531-2538. [DOI: 10.1016/j.sjbs.2020.04.033]

5. Alam MS, Alam MZ, Nazir K, et al. The emergence of novel coronavirus disease (COVID-19) in Bangladesh: Present status, challenges, and future management. *Journal of advanced veterinary and animal research*. 2020;7(2):198–208. [DOI: 10.5455/javar.2020.g410]

6. Banerjee S, Kushwaha AS, Akkilagunta S, et al. Strategies of India against coronavirus disease-2019: A strengths, weaknesses, opportunities, and threats analysis. *Med J DY Patil Vidyapeeth*. 2020;13:588-94. [DOI: 10.4103/mjdrdypu.mjdrdypu_509_20]

7. Baniya J, Bhattarai S, Pradhan V, et al. Visibility of Invisible: Covid-19 and Nepal-India Migration. *Tribhuvan University Journal*. 2020;34:101-114. [DOI: 10.3126/tuj.v34i0.31542]

8. Baral S, Rao A, Twahirwa Rwema JO, et al. Competing Health Risks Associated with the COVID-19 Pandemic and Response: A Scoping Review. *medRxiv [Preprint].* 2021. [DOI: 10.1101/2021.01.07.21249419]

9. Bashir MF, Benjiang MA, Shahzad L. A brief review of socio-economic and environmental impact of Covid-19. *Air Qual Atmos Health*. 2020;13:1403–1409. [DOI: 10.1007/s11869-020-00894-8]

10. Basnet S, Koirala S, Pandey B, et al. COVID-19 Containment Efforts of a Low-Resource Nation: The First Four Months in Nepal. *Cureus*. 2020;12(7):e8946. [DOI: 10.7759/cureus.8946]

11. Béné C. Resilience of local food systems and links to food security - A review of some important concepts in the context of COVID-19 and other shocks. *Food Secur*. 2020;1-18. [DOI: 10.1007/s12571-020-01076-1]

12. Bhutto SA, Shaikh S, Ashraf M, et al. The epidemic COVID-19 outbreak and economic slowdown in developing Asia. A review. *International Transaction Journal of Engineering, Management, & Applied Sciences & Technologies.* 2020;11(9).

13. Bielecki M, Patel D, Hinkelbein J, et al. Reprint of: Air travel and COVID-19 prevention in the pandemic and peri-pandemic period: A narrative review. *Travel Med Infect Dis*. 2020;38:101939. [DOI: 10.1016/j.tmaid.2020.101939]

14. Brooks SK, Webster RK, Smith LE, et al. (2020). The psychological impact of quarantine and how to reduce it: rapid review of the evidence. *The Lancet*. 2020;395(10227):912-920. [DOI: 10.1016/S0140-6736(20)30460-8]

15. Brown RCH, Savulescu J, Williams B, et al. Passport to freedom? Immunity passports for COVID-19. *J Med Ethics*. 2020;46(10):652-659. [DOI: 10.1136/medethics-2020-106365]

16. Bruinen de Bruin Y, Lequarre AS, McCourt J, et al. Initial impacts of global risk mitigation measures taken during the combatting of the COVID-19 pandemic. *Saf Sci*. 2020;128:104773. [DOI: 10.1016/j.ssci.2020.104773]

17. Burns J, Movsisyan A, Stratil JM, et al. Travel-related control measures to contain the COVID-19 pandemic: a rapid review. *Cochrane Database of Syst Rev*. 2020;10:CD013717 [DOI: 10.1002/14651858.CD013717]

18. Cappellano F, Kurowska-Pysz J. The Mission-Oriented Approach for (Cross-Border) Regional Development. *Sustainability*. 2020;12:5181. [DOI: 10.3390/su12125181]

19. Cardwell K, Jordan K, Byrne P, et al. The effectiveness of non‐contact thermal screening as a means of identifying cases of Covid‐19: a rapid review of the evidence. *Rev Med Virol*. 2020;e2192. [DOI: 10.1002/rmv.2192]

20. Cawthorn DM, Kennaugh A, Ferreira SM. The future of sustainability in the context of COVID-19. *Ambio*. 2020. [DOI: 10.1007/s13280-020-01430-9]

21. Ceravolo MG, de Sire A, Andrenelli E, et al. Systematic rapid "living" review on rehabilitation needs due to COVID-19: update to March 31st, 2020. *Eur J Phys Rehabil Med*. 2020;56(3):347-353. [DOI: 10.23736/S1973-9087.20.06329-7]

22. Çetin C, Kara A. Global surveillance, travel, and trade during a pandemic. *Turk J Med Sci*. 2020;50(SI-1):527-533. [DOI: 10.3906/sag-2004-175]

23. Chang MC, Baek JH, Park D. Lessons from South Korea Regarding the Early Stage of the COVID-19 Outbreak. *Healthcare*. 2020; 8(3):229. [DOI: 10.3390/healthcare8030229]

24. Chapman A, Tsuji T. Impacts of COVID-19 on a Transitioning Energy System, Society, and International Cooperation. *Sustainability*. 2020;12(19):8232. [DOI: 10.3390/su12198232]

25. Chatterjee P, Nagi N, Agarwal A, et al. The 2019 novel coronavirus disease (COVID-19) pandemic: A review of the current evidence. *Indian J Med Res*. 2020;151(2 & 3):147-159. [DOI: 10.4103/ijmr.IJMR_519_20]

26. Chen KZ & Mao R. Fire lines as fault lines: Increased trade barriers during the COVID-19 pandemic further shatter the global food system. *Food Security*. 2020;12:735–738. [DOI: 10.1007/s12571-020-01075-2]

27. Chen SL. Reviewing and Reflecting on Nursing During the COVID-19 Pandemic [in Chinese]. *Hu Li Za Zhi*. 2020;67(6):4-5. [DOI: 10.6224/JN.202012_67(6).01]

28. Chetty T, Daniels BB, Ngandu NK, et al. A rapid review of the effectiveness of screening practices at airports, land borders and ports to reduce the transmission of respiratory infectious diseases such as COVID-19. *S Afr Med J*. 2020;110(11):1105-1109. [DOI: 10.7196/samj.2020.v110i11.14959]

29. Chu IY, Alam P, Larson HJ, et al. Social consequences of mass quarantine during epidemics: a systematic review with implications for the COVID-19 response. *J Travel Med*. 2020;27(7):taaa192. [DOI: 10.1093/jtm/taaa192]

30. Chung SY, Chou FH. Lessons learned from SARS to COVID-19 in the Taiwanese population. *Asian J Psychiatr*. 2020;54:102299. [DOI: 10.1016/j.ajp.2020.102299]

31. D'cruz M, Banerjee D. 'An invisible human rights crisis': The marginalization of older adults during the COVID-19 pandemic - An advocacy review. *Psychiatry Res*. 2020;292:113369. [DOI: 10.1016/j.psychres.2020.113369]

32. Danielli S, Patria R, Donnelly P, et al. Economic interventions to ameliorate the impact of COVID-19 on the economy and health: an international comparison. *Journal of Public Health*.  2020;fdaa104. [DOI: 10.1093/pubmed/fdaa104]

33. Davahli MR, Karwowski W, Sonmez S, et al. The Hospitality Industry in the Face of the COVID-19 Pandemic: Current Topics and Research Methods. *Int J Environ Res Public Health*. 2020;17(20):7366. [DOI: 10.3390/ijerph17207366]

34. De Bruin YB, Lequarre AS, McCourt J, et al. Initial impacts of global risk mitigation measures taken during the combatting of the COVID-19 pandemic. *Safety Science*. 2020;128:104773. [DOI: 10.1016/j.ssci.2020.104773]

35. Dempster H, Zimmer C. Migrant Workers in the Tourism Industry: How has COVID-19 Affected Them, and What Does the Future Hold? *CGD Policy Paper 173*. Washington DC: Center for Global Development, 2020.

36. Doumbia-Henry C. Shipping and COVID-19: protecting seafarers as frontline workers. *WMU J Marit Affairs*. 2020;19:279–293. [DOI: 10.1007/s13437-020-00217-9]

37. Errett NA, Sauer LM, Rutkow L. An integrative review of the limited evidence on international travel bans as an emerging infectious disease disaster control measure. *J Emerg Manag*. 2020;18(1):7-14. [DOI: 10.5055/jem.2020.0446]

38. Flew T, Kirkwood K. The impact of COVID-19 on cultural tourism: art, culture and communication in four regional sites of Queensland, Australia. *Media International Australia.* 2021;178(1):16-20. [DOI: 10.1177/1329878X20952529]

39. Ghosh S, Ghosh S. Air quality during COVID-19 lockdown: Blessing in disguise. *IJBB*. 2020;57(4):420-430.

40. Girum T, Lentiro K, Geremew M, et al. Global strategies and effectiveness for COVID-19 prevention through contact tracing, screening, quarantine, and isolation: a systematic review. *Trop Med Health.* 2020;48:91. [DOI: 10.1186/s41182-020-00285-w]

41. Goniewicz K, Khorram-Manesh A, Hertelendy AJ, et al. Current Response and Management Decisions of the European Union to the COVID-19 Outbreak: A Review. *Sustainability*. 2020; 12(9):3838. [DOI: 10.3390/su12093838]

42. Gopal JP, Papalois VE. COVID-19 pandemic: Building organisational flexibility to scale transplant programs. *World J Transplant*. 2020;10(10):277-282. [DOI: 10.5500/wjt.v10.i10.277]

43. Grech V & Grech P. COVID-19: Combined supply-side and demand-side shocks, so lift restrictions (carefully) lest GPD declines ultimately kill more than COVID-19. *Early human development*. 2020: 105209. [DOI: 10.1016/j.earlhumdev.2020.105209]

44. Grépin KA, Ho TL, Liu Z, et al. Evidence of the effectiveness of travel-related measures during the early phase of the COVID- 19 pandemic: a rapid systematic review. *medRxiv [Preprint]*. 2020. [DOI: 10.1101/2020.11.23.20236703]

45. Guntupalli Y, Priya AJ, Mohanraj KG, et al. Economic declination due to COVID-19 lockdown – A review. *European Journal of Molecular & Clinical Medicine*. 2020;7(1):2289-2295.

46. Ha BTT, Ngoc Quang L, Mirzoev T, et al. Combating the COVID-19 Epidemic: Experiences from Vietnam. *Int J Environ Res Public Health*. 2020;17(9):3125. [DOI: 10.3390/ijerph17093125]

47. Hamzelou J. The countries getting it right. *New Scientist*. 2020;245(3274):8-9. [DOI: 10.1016/S0262-4079(20)30570-4]

48. Han E, Tan MMJ, Turk E, et al. Lessons learnt from easing COVID-19 restrictions: an analysis of countries and regions in Asia Pacific and Europe. *Lancet*. 2020;396(10261):1525-1534. [DOI: 10.1016/S0140-6736(20)32007-9]

49. Harshal V, Simran M. Impact of COVID-19 pandemic situation on consumer buying behaviour in Indian market - a review. *International Journal for Research in Applied Science and Engineering Technology*. 2020;8(5):2584-2589. [DOI: 10.22214/ijraset.2020.5429]

50. Hossain MM, Sultana A, Purohit N. Mental health outcomes of quarantine and isolation for infection prevention: a systematic umbrella review of the global evidence. *Epidemiol Health*. 2020;42:e2020038. [DOI: 10.4178/epih.e2020038]

51. Hussain A, Yadav S, Hadda V, et al. Covid-19: a comprehensive review of a formidable foe and the road ahead. *Expert Rev Respir Med*. 2020;14(9):869-879. [DOI: 10.1080/17476348.2020.1782198]

52. Imai N, Gaythorpe KAM, Abbott S, et al. Adoption and impact of non-pharmaceutical interventions for COVID-19. *Wellcome Open Res*. 2020;5:59. [DOI: 10.12688/wellcomeopenres.15808.1]

53. Jomo KS, Chowdhury A. COVID-19 Pandemic Recession and Recovery. *Development (Rome).* 2020:1-12. [DOI: 10.1057/s41301-020-00262-0]

54. Jumba FR, Tibasiima T, Byaruhanga E, et al. COVID 19: Lets act now: the urgent need for upscaling agroecology in Uganda (2020). *International Journal of Agricultural Sustainability*. 2020;18(6):449-455. [DOI: 10.1080/14735903.2020.1794428]

55. Kang S, Moon J, Kang H, et al. The Evolving Policy Debate on Border Closure in Korea. *J Prev Med Public Health*. 2020;53(5):302-306. [DOI: 10.3961/jpmph.20.213]

56. Khan G, Sheek-Hussein M, Al Suwaidi AR, et al. Novel coronavirus pandemic: A global health threat. *Turk J Emerg Med*. 2020;20(2):55-62. [DOI: 10.4103/2452-2473.285016]

57. Khan I, Shah D, Shah SS. COVID-19 pandemic and its positive impacts on environment: an updated review. *Int J Environ Sci Technol*. 2021;18:521–530. [DOI: 10.1007/s13762-020-03021-3]

58. Khatib AN, Carvalho AM, Primavesi R, To K, Poirier V. Navigating the risks of flying during COVID-19: a review for safe air travel. *J Travel Med*. 2020;27(8):taaa212. [DOI: 10.1093/jtm/taaa212]

59. Kizielewicz J. COVID-19 Consequences and Travel Insurance Policy in Leading Cruise Shipping Corporations. *European Research Studies Journal*. 2020;0(4):600-611.

60. Kumar R, Dhiman B. Impact of COVID-19 and its containment measures on stock market: A review at nascent stage. *Wesleyan Journal of Research*. 2020;22(3).

61. Lal P, Kumar A, Kumar S, et al. The dark cloud with a silver lining: Assessing the impact of the SARS COVID-19 pandemic on the global environment. *Sci Total Environ*. 2020;732:139297. [DOI: 10.1016/j.scitotenv.2020.139297]

62. Laws K & Ammigan R. International Students in the Trump Era: A Narrative View. *Journal of International Students*. 2020;10(3):xviii-xxii. [DOI: 10.32674/jis.v10i3.2001]

63. Liang H, Zheng L, Xia H, Tang J. SARS-CoV-2 infection in China—Before the pandemic. *PLOS Neglected Tropical Diseases*. 2020;14(8):e0008472. [DOI: 10.1371/journal.pntd.0008472]

64. Liu NN, Tan JC, Li J, et al. COVID-19 Pandemic: Experiences in China and Implications for its Prevention and Treatment Worldwide*. Curr Cancer Drug Targets.* 2020;20(6):410-416. [DOI: 10.2174/1568009620666200414151419]

65. Matiza T. Post-COVID-19 crisis travel behaviour: towards mitigating the effects of perceived risk. *Journal of Tourism Futures*. 2020. [DOI: 10.1108/JTF-04-2020-0063]

66. Mouchtouri VA, Bogogiannidou Z, Dirksen-Fischer M, et al. Detection of imported COVID-19 cases worldwide: early assessment of airport entry screening, 24 January until 17 February 2020. *Trop Med Health 48*. 2020. [DOI: 10.1186/s41182-020-00260-5]

67. Muhammad A, Ali J, Manan A, et al. Strategies to control and prevent novel coronavirus 2019: A quick overview. *J Liaquat Univ Med Health Sci*. 2020;1(19):1-5.

68. Muhammad A, Muhammad O, Ali N, et al. COVID-19 pandemic and precautionary measures in Pakistan. *Anaesthesia, Pain and Intensive Care.* 2020,24(1):94-100. [DOI: 10.35975/apic.v24i1.1231]

69. Mukhtar S. Psychological health during the coronavirus disease 2019 pandemic outbreak. *Int J Soc Psychiatry*. 2020;66(5):512-516. [DOI: 10.1177/0020764020925835]

70. Nicola M, Alsafi Z, Sohrabi C, et al. The socio-economic implications of the coronavirus pandemic (COVID-19): A review. *Int J Surg*. 2020;78:185-193. [DOI: 10.1016/j.ijsu.2020.04.018]

71. Nussbaumer-Streit B, Mayr V, Dobrescu AI, et al. Quarantine alone or in combination with other public health measures to control COVID-19: a rapid review. *Cochrane Database Syst Rev*. 2020;4(4):CD013574. [DOI: 10.1002/14651858.CD013574]

72. Ogunleye OO, Basu D, Mueller D, et al. Response to the Novel Corona Virus (COVID-19) Pandemic Across Africa: Successes, Challenges, and Implications for the Future. *Front Pharmacol*. 2020;11:1205. [DOI: 10.3389/fphar.2020.01205]

73. Oti VB, Ioannou M. Traveler`s Infections: Understanding SARS-CoV-2 as a Potential Agent. *Jurnal Kesehatan Masyarakat Nasional*. 2020;SpecialIssue1:64-69. [DOI: 10.21109/kesmas.v15i2.3974]

74. Pandey S, Yadav B, Pandey A, et al. Lessons from SARS-CoV-2 Pandemic: Evolution, Disease Dynamics and Future. *Biology (Basel)*. 2020;9(6):141. [DOI: 10.3390/biology9060141]

75. Patiño-Lugo DF, Vélez M, Velásquez Salazar P, et al. Non-pharmaceutical interventions for containment, mitigation and suppression of COVID-19 infection. *Colomb Med (Cali)*. 2020;51(2):e4266. [DOI: 10.25100/cm.v51i2.4266]

76. Poudel PB, Poudel MR, Gautam A, et al. COVID-19 and its Global Impact on Food and Agriculture. *J Biol Today´s World*. 2020;9(5):221.

77. Ratten V. Coronavirus and international business: An entrepreneurial ecosystem perspective. Thunderbird International Business Review. 2020;62(5):629-634. [DOI: 10.1002/tie.22161]

78. Regmi K, Lwin CM. Impact of non-pharmaceutical interventions for reducing transmission of COVID-19: a systematic review and meta-analysis protocol. *BMJ Open*. 2020;10(10):e041383. [DOI: 10.1136/bmjopen-2020-041383]

79. Rejeb A, Rejeb K, Keogh JG. COVID-19 and the Food Chain? Impacts and future research trends. *LogForum*. 2020;16(4):475-485. [DOI: 10.17270/J.LOG.2020.502]

80. Ryan J, Mazingisa A, Wiysonge CS. Cochrane corner: effectiveness of quarantine in reducing the spread of COVID-19. *Pan African Medical Journal*. 2020;35(2):18 [DOI: 10.11604/pamj.2020.35.2.23051]

81. Şencan I, Kuzi S. Global threat of COVID 19 and evacuation of the citizens of different countries. *Turk J Med Sci*. 2020;50(SI-1):534-543. [DOI: 10.3906/sag-2004-21]

82. Shah SA, Mansor J, Nurumal SR, et al. Rapid Response and Public Health Measures of COVID-19 Infection Among Asian Countries. *Gazi Medical Journal*. 2020;31(2A).

83. Tanvi G, Yash N. Using Data Analytics to Determine the Disruptions in Supply Chain Due to the Covid-19 Pandemic: A Literature Review. *IJRASET*. 2020;8(5). [DOI: 10.22214/ijraset.2020.5191]

84. Turanjanin V, Radulovic D. Coronavirus (COVID-19) and Possibilities for Criminal Law Reaction in Europe: A Review*. Iran J Public Health*. 2020;49(Suppl.1):4-11.

85. University of Michigan (USA), Chair persons: King EJ - USA, Greer S - USA, 6.A. Round table: Protecting borders and preparing for a pandemic: responses around the world to COVID19. *European Journal of Public Health*. 2020;30(Suppl 5): ckaa165.283. [10.1093/eurpub/ckaa165.283]

86. Usher K, Jackson D, Durkin J, et al. Pandemic‐related behaviours and psychological outcomes; A rapid literature review to explain COVID‐19 behaviours. *International Journal of Mental Health Nursing*. 2020;29(6):1018-1034. [DOI: 10.1111/inm.12790]

87. Vaidya R, Herten-Crabb A, Spencer J, et al. Travel restrictions and infectious disease outbreaks. *J Travel Med*. 2020;27(3):taaa050. [DOI: 10.1093/jtm/taaa050]

88. Vasishta PA, Anjali AK, Brundha MP, et al. An overview on pandemic Covid-19. *International Journal of Research in Pharmaceutical Sciences*. 2020;11(SPL1):696-700. [DOI: 10.26452/ijrps.v11iSPL1.3068]

89. Wang F, Tian C, Qin W. The impact of epidemic infectious diseases on the wellbeing of migrant workers: A systematic review. *International Journal of Wellbeing*. 2020;10(3):7-25. [DOI: 10.5502/ijw.v10i3.1301]

90. Weber M, Blackwood MA, Husted TF, et al. Global Democracy and Human Rights Impacts of COVID-19: In Brief [June 26, 2020]. Congressional Research Service. Washington DC, 2020.

91. Xu W, Wu J, Cao L. COVID-19 pandemic in China: Context, experience and lessons*. Health Policy Technol*. 2020;9(4):639-648. [DOI: 10.1016/j.hlpt.2020.08.006]

92. Yuksel B, Ozgor F. Effect of the COVID-19 pandemic on female sexual behavior. *Int J Gynaecol Obstet*. 2020 Jul;150(1):98-102. [DOI: 10.1002/ijgo.13193]

93. Zanin M, Xiao C, Liang T, et al. The public health response to the COVID-19 outbreak in mainland China: a narrative review*. J Thorac Dis*. 2020;12(8):4434-4449. [DOI: 10.21037/jtd-20-2363]

94. Zhou JQ, Dong W, Xu HL, et al. Pay attention to the exposure risk of patients with chronic wounds on the way to hospital during coronavirus disease 2019 epidemic prevention and control [in Chinese]. *Zhonghua Shao Shang Za Zhi*. 2020;36(6):469-471. [DOI: 10.3760/cma.j.cn501120-20200218-00065]

95. Zodpey S, Negandhi H, Dua A, et al. Our Fight Against the Rapidly Evolving COVID-19 Pandemic: A Review of India's Actions and Proposed Way Forward. *Indian J Community Med*. 2020;45(2):117-124. [DOI: 10.4103/ijcm.IJCM_221_20]

96. Zoumpourlis V, Goulielmaki M, Rizos E, et al. [Comment] The COVID‑19 pandemic as a scientific and social challenge in the 21st century. *Mol Med Rep*. 2020;22(4):3035-3048. [DOI: 10.3892/mmr.2020.11393]

## Appendix 5. Data extraction form

Study information

- Study ID
- Study title
- Study source (i.e., journal, report, pre-print publication)
- Date of submission
- Date of publication

Study characteristics

- Study design
  - Quantitative (modelling)
  - Quantitative (other)
  - Qualitative
  - Mixed-methods
- Verbal summary of study design (e.g., observational study, interrupted time series study)
- Comments

Setting and context

- Country/countries in which travel-related control measure is implemented
- Country/countries restricted by travel-related control measure
- Mode of travel
  - Air
  - Land
  - Sea
  - Any combination of the above
  - Not specified
- Comments

Population

- Description of the targeted population, i.e., the population affected by the travel-related control measure (e.g., international travelers arriving between 27 April and 22 June 2020)
- Description of the studied population, i.e., the population in which the outcome is assessed (e.g., families of international travelers arriving between 27 April and 22 June 2020)
- Comments

Intervention

- Broad intervention category
  - Closure of national borders to entry/exit
  - International travel restrictions
  - Entry/exit screening at national borders
  - Quarantine or isolation of travelers crossing national borders
  - Any combination of the above
- Verbal summary of specific travel-related control measure(s) (e.g., all arriving passengers were provided with a symptom questionnaire)
- Date(s) of implementation of the travel-related control measure(s)
- Duration of the intervention
- Any reported exceptions to the measure (e.g., certain individuals being excluded or fast-tracked in airport screening because of nationality, occupation, country of origin)
- Representation of the intervention in the model (e.g., 100% travel reduction)
- Comparisons/counterfactual scenario(s)
- Comments

Outcomes (repeated for each outcome)

- Outcome category
  - Quality of life, social well-being, and mental health
  - Physical health, health behavior, health risks, and healthcare beyond COVID-19
  - Equity, equality and the fair distribution of benefits and burdens
  - Social
  - Environmental
  - Economic
  - Other (to be specified)
- Specification of additional outcome category
- Verbal description of outcome
- Study period
- Length of follow-up
- Narrative summary of findings
- Comments

Conflicts of interest statements and funding sources

## Appendix 6. References to studies excluded from this review and reason for exclusion

| **Study ID** | **Reference** | **Reason for exclusion** |
| --- | --- | --- |
| Aborode_2020 | Aborode AT, Ogunsola SO, Adeyemo AO. A Crisis within a Crisis: COVID-19 and Hunger in African Children. *Am J Trop Med Hyg*. 2020;00(0):1-1. [DOI: 10.4269/ajtmh.20-1213] | Study type |
| Aburumman_2020 | Aburumman AA. COVID-19 impact and survival strategy in business tourism market: the example of the UAE MICE industry. *Humanit Soc Sci Commun*. 2020;7:141. [DOI: 10.1057/s41599-020-00630-8] | Intervention |
| Acharya_2020 | Acharya BK, Khanal L, Mahyoub ASM, et al. Execution of intervention matters more than strategy: A lesson from the spatiotemporal assessment of COVID-19 clusters in Nepal. *medRxiv [Preprint].*2020. [DOI: 10.1101/2020.11.07.20227520] | Outcome |
| Adnan_2020 | Adnan N, Nordin SMd. How COVID 19 efect Malaysian paddy industry? Adoption of green fertilizer a potential resolution. *Environ Dev Sustain*. 2020. [DOI: 10.1007/s10668-020-00978-6] | Study type |
| Aiken_2020 | Aiken ARA, Starling JE, Gomperts R, et al. Demand for Self-Managed Online Telemedicine Abortion in Eight European Countries During the COVID-19 Pandemic: A Regression Discontinuity Analysis. *medRxiv [Preprint]*. 2020. [DOI: 10.1101/2020.09.15.20195222] | Intervention |
| Akca_2020 | Akca ÖÜM. COVID-19’UN HAVACILIK SEKTÖRÜNE ETKİSİ [in Turkish]. *Avrasya Sosyal ve Ekonomi Araştırmaları Dergisi (ASEAD).*2020;7(5):45-64. | Study type |
| Akyol_2020 | Akyol C. Opinions of undergraduate tourism students on the COVID-19 outbreak [in Turkish]. *Journal of Gastronomy, Hospitality, and Travel (JOGHAT)*. 2020;3(1). [DOI: 10.33083/joghat.2020.35] | Intervention |
| Alahadal_2020 | Alahdal A, Aldhali FIA, Bahari A. Coronavirus (Covid-19) and Mental Health Concerns of University Students in KSA: An Empirical Study at Qassim University*. The Asian EFL Journal*. 2020;27(4.4). | Intervention |
| Alhassan_2020 | Alhassan SI, Akoto JD, Ackah M, et al. Assessing the impacts of COVID-19 pandemic on the environment: A correlation or causation? *Glob J Ecol.* 2020;5(1): 095-098. [DOI: 10.17352/gje.000027] | Study type |
| Ali_2020 | Ali MA, Al-Khani AM, Sidahmed LA. Migrant health in Saudi Arabia during the COVID-19 pandemic. *East Mediterr Health J*. 2020;26(8):879-880. [DOI: 10.26719/emhj.20.094] | Study type |
| Alizargar_2020 | Alizargar J. Home quarantine for Taiwanese travelers entering Taiwan. *J Formos Med Assoc*. 2020;119(7):1236. [DOI: 10.1016/j.jfma.2020.05.010] | Study type |
| Alqutob_2020 | Alqutob R, Al Nsour M, Tarawneh MR, et al. COVID-19 Crisis in Jordan: Response, Scenarios, Strategies, and Recommendations. *JMIR Public Health Surveill.*2020;6(3):e19332. [DOI: 10.2196/19332] | Intervention |
| Alsayedahmed_2020 | Alsayedahmed HH. COVID-19 Pandemic’s precautionary measures had hit the reset button of the quality of life at different aspects. *J Infect Dev Ctries*. 2020;14(8):812-816. [DOI: 10.3855/jidc.12943] | Study type |
| Alser_2020 | Alser O, AlWaheidi S, Elessi K, et al. COVID-19 in Gaza: a pandemic spreading in a place already under protracted lockdown. *East Mediterr Health J.*2020;26(7):762-763. [DOI: 10.26719/emhj.20.089] | Study type |
| Altuntas_2021 | Altuntas F, Gok MS. The effect of COVID-19 pandemic on domestic tourism: A DEMATEL method analysis on quarantine decisions.*Int J Hosp Manag.*2021;92:102719. [DOI: 10.1016/j.ijhm.2020.102719] | Intervention |

Appendix 6 (continued)

| **Study ID** | **Reference** | **Reason for exclusion** |
| --- | --- | --- |
| Alzueta_2020 | Alzueta E, Perrin P, Baker FC, et al. How the COVID‐19 pandemic has changed our lives: A study of psychological correlates across 59 countries. *J Clin Psychol*. 2020;1–15. [DOI: 10.1002/jclp.23082] | Intervention |
| Amankwah-Amoah_2020 | Amankwah-Amoah J. Note: Mayday, Mayday, Mayday! Responding to environmental shocks: Insights on global airlines’ responses to COVID-19. *Transp Res E Logist Transp Rev*. 2020;143:102098. [DOI: 10.1016/j.tre.2020.102098] | Study type |
| Andam_2020 | Andam K, Edeh H, Oboh V, et al. Impacts of COVID-19 on food systems and poverty in Nigeria. *Advances in Food Security and Sustainability*. 2020;5:145-173. [DOI: 10.1016/bs.af2s.2020.09.002] | Intervention |
| Anderson_2020 | Anderson SC, Mulberry N, Edwards AM, et al. How much leeway is there to relax COVID-19 control measures? *medRxiv [Preprint].* 2020. [DOI: 10.1101/2020.06.12.20129833] | Outcome |
| Anonymous_2020 | Brooks C, Szakonyl M, Meyer B, et al. Containing cororavirus. *The Journal of Commerce*. 2020. | Study type |
| Aravindakshan_2020 | Aravindakshan A, Boehnke J, Gholami E, et al. Preparing for a future COVID‐19 wave: insights and limitations from a data‐driven evaluation of non‐pharmaceutical interventions in Germany. *Sci Rep.*2020;10:20084. [DOI: 10.1038/s41598-020-76244-6] | Outcome |
| Arctec_2020 | Arctec. Canine COVID-19 Detection. *ClinicalTrials.gov.*2020. | Intervention |
| Ardila-Sierra_2020 | Ardila-Sierra A, Niño-Leal L, Rivera-Triana D, et al. Condiciones en la frontera sur entre Colombia y Venezuela ante la pandemia de COVID-19 [in Spanish]. *Rev Salud Pública*. 2020;22(2):1-9. [DOI: 10.15446/rsap.v22n2.86366] | Non-Covid-19 |
| Ashraf_2020 | Ashraf BN. Economic impact of government interventions during the COVID-19 pandemic: International evidence from financial markets. *Journal of Behavioral and Experimental Finance*. 2020;27:100371. [DOI: 10.1016/j.jbef.2020.100371] | Intervention |
| Ayittey_2020 | Ayittey FK, Ayittey MK, Chiwero NB, et al. Economic impacts of Wuhan 2019‐nCoV on China and the world. *J Med Virol.*2020;92(5):473-475. [DOI: 10.1002/jmv.25706] | Study type |
| Ayub_2020 | Ayub AJ. Projecting the impact of behaviour and isolation interventions and super spreader events from mass gatherings and international travel on Malaysia's COVID-19 epidemic trajectories using an augmented SEIR model. *medRxiv [Preprint].*2020. [DOI: 10.1101/2020.10.29.20222224] | Outcome |
| Bakar_2020 | Bakar NA, Rosbi S. Effect of Coronavirus disease (COVID-19) to tourism industry. *International Journal of Advanced Engineering Research and Science (IJAERS)*. 2020;7(4). [DOI: 10.22161/ijaers.74.23] | Intervention |
| Baker_2020 | Baker SR, Bloom N, Davis SJ, et al. The Unprecedented Stock Market Reaction to COVID-19. *The Review of Asset Pricing Studies*. 2020;10:742–758. [DOI: 10.1093/rapstu/raaa008] | Intervention |
| Bakhiet_2020 | Bakhiet Z, Norman K, Moore Q. Building a New Life in Uncertain Times: The Impact of Covid-19 on Refugees in the U.S. Policy Brief. *Rice University´s Baker Institute for Public Policy*. 2020. | Study type |
| Bakhturazova_2020 | Bakhturazova TW, Mayorov MK, Mayorova NV, et al. Threats to industrial policy, trade and knowledge sharing in a global emergency [in Russian]. *Вестник университета*. 2020;4. [DOI: 10.26425/1816-4277-2020-4-42-46] | Study type |

Appendix 6 (continued)

| **Study ID** | **Reference** | **Reason for exclusion** |
| --- | --- | --- |
| Baldasano_2020 | Baldasano JM. COVID-19 lockdown effects on air quality by NO2 in the cities of Barcelona and Madrid (Spain). *Sci Total Environ.*2020;741:140353. [DOI: 10.1016/j.scitotenv.2020.140353] | Intervention |
| Banerjee_2020 | Banerjee D, Nair VS. “The Untold Side of COVID-19”: Struggle and Perspectives of the Sexual Minorities. *Journal of Psychosexual Health*. 2020;2(2):113-120. [DOI: 10.1177/2631831820939017] | Study type |
| Bao_2020 | Bao R, Zhang A. Does lockdown reduce air pollution? Evidence from 44 cities in northern China. *Sci Total Environ.*2020;731:139052. [DOI: 10.1016/j.scitotenv.2020.139052] | Intervention |
| Baral_2021 | Baral S, Rao A, Twahirwa Rwema JO, et al. Competing Health Risks Associated with the COVID-19 Pandemic and Response: A Scoping Review. *medRxiv [Preprint]*. 2020. [DOI: 10.1101/2021.01.07.21249419] | Study type |
| Barbieri_2020 | Barbieri DM, Lou B, Passavanti M, et al. A survey dataset to evaluate the changes in mobility and transportation due to COVID-19 travel restrictions in Australia, Brazil, China, Ghana, India, Iran, Italy, Norway, South Africa, United States. *Data in Brief.*2020;33:106459. [DOI: 10.1016/j.dib.2020.106459] | Intervention |
| Barguil_2020 | Barguil Y, Chiaradia L, Sicard D, et al. Management of a global health crisis: first COVID-19 disease feedback from Overseas and French-speaking countries medical biologists. *Ann Biol Clin (Paris).* 2020;78(5):499-518. [DOI: 10.1684/abc.2020.1586] | Study type |
| Basit_2020 | Basit A. COVID-19: a challenge or opportunity for terrorist groups? *Journal of Policing, Intelligence and Counter Terrorism*. 2020;15(3):263-275. [DOI: 10.1080/18335330.2020.1828603] | Study type |
| Bich-Ngoc_2020 | Bich-Ngoc N, Teller J. Potential Effects of the COVID-19 Pandemic through Changes in Outbound Tourism on Water Demand: The Case of Liège (Belgium). *Water*. 2020;12(10):2820. [DOI: 10.3390/w12102820] | Intervention |
| Binny_2020 | Binny RN, Baker MG, Hendy SC, et al. Early intervention is the key to success in COVID-19 control. *medRxiv [Preprint]*. 2020. [DOI: 10.1101/2020.10.20.20216457] | Outcome |
| Biswal_2020 | Biswal A, Singh T, Singh V, et al. COVID-19 lockdown and its impact on tropospheric NO2 concentrations over India using satellite-based data. *Heliyon*. 2020;6(8):e04764. [DOI: 10.1016/j.heliyon.2020.e04764] | Intervention |
| Bixby_2020 | Bixby M, Hoover SE, McCallum R, et al. Honey Bee Queen Production: Canadian Costing Case Study and Profitability Analysis. *J Econ Entomol*. 2020;113(4):1618-1627. [DOI: 10.1093/jee/toaa102] | Non-Covid-19 |
| Bonaccorsi_2020 | Bonaccorsi G, Pierri F, Cinelli M, et al. Economic and social consequences of human mobility restrictions under COVID-19. *Proc Natl Acad Sci U S A*. 2020;117(27):15530-15535. [DOI: 10.1073/pnas.2007658117] | Intervention |
| Boretti_2020 | Boretti A. Covid19 pandemic as a further driver of water scarcity in Africa. *GeoJournal*. 2020;Aug 25:1-28. [DOI: 10.1007/s10708-020-10280-7] | Study type |
| Boros_2020 | Boros L, Dudás G, Kovalcsik T. The effects of COVID-19 on Airbnb. *Hungarian Geographical Bulletin.*2020;69(4):363–381. [DOI: 10.15201/hungeobull.69.4.3] | Intervention |
| Bouali_2020 | Bouali S, Douha S, Khadri N. To what extent is air freight affected by the Corona virus pandemic? *Journal of Sustainable Development of Transport and Logistics*. 2020;5(2):98-108. [DOI: 10.14254/jsdtl.2020.5-2.9] | Intervention |

Appendix 6 (continued)

| **Study ID** | **Reference** | **Reason for exclusion** |
| --- | --- | --- |
| Bouillon-Minois_2020 | Bouillon-Minois JB, Lesage FX, Schmidt J, et al. Coronavirus and Exceptional Health Situations: The First Disaster With Benefits on Air Pollution. *Disaster Medicine and Public Health Preparedness.*2020;14(3). [DOI: 10.1017/dmp.2020.174] | Study type |
| Brammer_2020 | Brammer S, Branicki L, Linnenluecke MK. COVID-19, Societalization, and the Future of Business in Society. *Academy of Management Perspectives.*2020;34(4):493-507. [DOI: 10.5465/amp.2019.0053] | Study type |
| Bramstedt_2020 | Bramstedt KA. Antibodies as Currency: COVID-19’s Golden Passport. *Bioethical Inquiry.*2020;17:687–689. [DOI: 10.1007/s11673-020-09996-5] | Study type |
| Branicki_2020 | Branicki LJ. COVID-19, ethics of care and feminist crisis management. *Gender Work Organ*. 2020;27:872–883. [DOI: 10.1111/gwao.12491] | Study type |
| Brimblecombe_2020 | Brimblecombe P, Lai Y. Diurnal and weekly patterns of primary pollutants in Beijing under COVID-19 restrictions. *Faraday Discuss*. 2020. [DOI: 10.1039/D0FD00082E] | Intervention |
| Bruns_2020 | Bruns DP, Kraguljac NV, Bruns TR. COVID-19: Facts, Cultural Considerations, and Risk of Stigmatization. *Journal of Transcultural Nursing.*2020;31(4):326–332. [DOI: 10.1177/1043659620917724] | Study type |
| Buckley_2020 | Buckley R. Pandemic Travel Restrictions Provide a Test of Net Ecological Effects of Ecotourism and New Research Opportunities. *Journal of Travel Research*. 2020, Aug. [DOI: 10.1177/0047287520947812] | Study type |
| Budd_2020 | Budd L, Ison S, Adrienne N. European airline response to the COVID-19 pandemic – Contraction, consolidation and future considerations for airline business and management. *Research in Transportation Business & Management*. 2020;37:100578. [DOI: 10.1016/j.rtbm.2020.100578] | Intervention |
| Buizza_2020 | Buizza R. Affrontare il cambiamento climatico è una ‘missione possibile’. *G Clin Nefrol Dial.*2020;32:154-160. [DOI: 10.33393/gcnd.2020.2206] | Non-Covid-19 |
| Burda_2020 | Burda, Z. Modelling Excess Mortality in Covid-19-like Epidemics. *Entropy*. 2020;22:1236. [DOI: 10.3390/e22111236] | Intervention |
| Caligiuri_2020 | Caligiuri P, de Cieri H, Minbaeva D, et al. International HRM insights for navigating the COVID-19 pandemic: Implications for future research and practice. *J Int Bus Stud*. 2020;51:697-713. [DOI: 10.1057/s41267-020-00335-9] | Study type |
| Callander_2020 | Callander D, Meunier E, DeVeau R, et al. Investigating the effects of COVID-19 on global male sex work populations: a longitudinal study of digital data. *Sex Transm Infec*t. 2020;0:1–6. [DOI: 10.1136/sextrans-2020-054550] | Intervention |
| Castka_2020 | Castka P, Searcy C, Fischer S. Technology-enhanced Auditing in Voluntary Sustainability Standards: The Impact of COVID-19. *Sustainability*. 2020;12:4740. [DOI: 10.3390/su12114740] | Intervention |
| Centeno_2020 | Centeno RS, Marquez JP. How much did the Tourism Industry Lost? Estimating Earning Loss of Tourism in the Philippines.*arXiv [Preprint]*. 2020;Apr. | Intervention |
| Chan_CP_2020 | Chan CP, Wong NS, Leung CC, et al. Positive impact of measures against COVID-19 on reducing influenza in the Northern Hemisphere. *J Travel Med.*2020;27(8):taaa087. [DOI: 10.1093/jtm/taaa087] | Intervention |

Appendix 6 (continued)

| **Study ID** | **Reference** | **Reason for exclusion** |
| --- | --- | --- |
| Chang_2020 | Chang K, Pan CY, Lu PL. Sentinel surveillance at airports: Experience of dengue and COVID-19 prevention in Taiwan. *Kaohsiung J Med Sci*. 2020;36(8):665-666. [DOI: 10.1002/kjm2.12265] | Outcome |
| Chaudhary_2020 | Chaudhary M, Sodani PR, Das S. Effect of COVID-19 on Economy in India: Some Reflections for Policy and Programme. *Journal of Health Management.*2020;22(2):169-180. [DOI: 10.1177/0972063420935541] | Study type |
| Chaudhry_2020 | Chaudhry R, Dranitsaris G, Mubashir T. A country level analysis measuring the impact of government actions, country preparedness and socioeconomic factors on COVID-19 mortality and related health outcomes. *EClinicalMedicine*. 2020;25:100464. [DOI: 10.1016/j.eclinm.2020.100464] | Outcome |
| Chee_2020 | Chia RCJ, Liew VKS, Rowland R. Daily New Covid-19 Cases, the Movement Control Order, and Malaysian Stock Market Returns. *International Journal of Business and Society*. 2020;21(2):553-568. | Intervention |
| Chen_CHS_2020 | Chen CHS, Cheng TJ. Reduction of Influenza and Enterovirus Infection in Taiwan during the COVID-19 Pandemic. *Aerosol and Air Quality Research*, 2020;20: 2071–2074. [DOI: 10.4209/aaqr.2020.05.0248] | Intervention |
| Chen_K_2020 | Chen KZ, Zhan Y, Zhang Y, et al. The impacts of COVID-19 on global food security and the coping strategy [in Chinese]. *Chinese Rural Economy*. 2020. | Study type |
| Chen_MH_2020 | Chen MH, Demir E, García-Gómez CD, et al. The impact of policy responses to COVID-19 on U.S. travel and leisure companies. *Annals of Tourism Research Empirical Insights*. 2020;1(1):100003. [DOI: 10.1016/j.annale.2020.100003] | Intervention |
| Chen_T_2020 | Chen T, Huang S, Li G, et al. Quantitative Effects of Entry Restrictions and Travel Quarantine on the Next Wave of COVID-19: Case Studies of China and Singapore. *SSRN [Preprint]*. 2020. | Outcome |
| Cheng_HY_2020 | Cheng HY, Chueh YN, Chen CM, et al. Taiwan’s COVID-19 response: Timely case detection and quarantine, January to June 2020. *J Formos Med Assoc*. 2020;Nov 2:S0929-6646(20)30502-7. [DOI: 10.1016/j.jfma.2020.10.023] | Study type |
| Cheng_HY_2020_2 | Cheng HY, Huang ASE. Proactive and blended approach for COVID-19 control in Taiwan. *Biochem Biophys Res Commun*. 2020;Nov 6:S0006-291X(20)32029-5. [DOI: 10.1016/j.bbrc.2020.10.100] | Study type |
| Cheng_HY_2020_3 | Cheng HY, Chueh YN, Chen CM, et al. Taiwan’s COVID-19 response: Timely case detection and quarantine, January to June 2020. *J Formos Med Assoc*. 2020;Nov 2:S0929-6646(20)30502-7. [DOI: 10.1016/j.jfma.2020.10.023] | Duplicate |
| Chevallier_2020 | Chevallier F, Zheng B, Broquet G, et al. Local Anomalies in the Column‐Averaged Dry Air Mole Fractions of Carbon Dioxide Across the Globe During the First Months of the Coronavirus Recession. recession. *Geophysical Research Letters*. 2020;47:e2020GL090244. [DOI: 10.1029/2020GL090244] | Intervention |
| Chiew_2020 | Chiew CJ, Li Z, Lee VJ. Reducing onward spread of COVID-19 from imported cases: quarantine and “stay at home” measures for travellers and returning residents to Singapore. *J Travel Med.*2020;27(3):taaa049. [DOI: 10.1093/jtm/taaa049] | Study type |

Appendix 6 (continued)

| **Study ID** | **Reference** | **Reason for exclusion** |
| --- | --- | --- |
| Chire Saire_2020 | Chire Saire JE, Oblitas Cruz JF. Study of Coronavirus Impact on Parisian Population from April to June using Twitter and Text Mining Approach. *medRxiv [Preprint]*. 2020. [DOI: 10.1101/2020.08.15.20175810] | Intervention |
| Choudhury_2020 | Choudhury P, Koo WW, Kishore N, et al. Food Security and Human Mobility During the Covid-19 Lockdown - Working Paper 20-113. *HBS Working Paper Series.*2020. | Intervention |
| Clipman_2020 | Clipman SJ, Wesolowski AP, Gibson DG, et al. Rapid real-time tracking of non-pharmaceutical interventions and their association with SARS-CoV-2 positivity: The COVID-19 Pandemic Pulse Study. *Clin Infect Dis*. 2020;Sep 2:ciaa1313. [DOI: 10.1093/cid/ciaa1313] | Intervention |
| Colavita_2020 | Colavita F, Vairo F, Meschi S, et al. COVID-19 Antigen Rapid Test as Screening Strategy at the Points-of-Entry: Experience in Lazio Region, Central Italy, August-October 2020. *medRxiv [Preprint]*. 2020. [DOI: 10.1101/2020.11.26.20232728] | Outcome |
| Comite_2020 | Comite U. Businesses and Public Health between using Lock Down as a Tool against Covid-19 pandemic in Italy: The Impact in a Global Perspective. *Advances in Management.*2020;13(2). | Study type |
| Cowling_2020 | Cowling BJ, Ali ST, Ng TWY, et al. Impact assessment of non-pharmaceutical interventions against coronavirus disease 2019 and influenza in Hong Kong: an observational study. *Lancet Public Health*. 2020;5: e279–88. [DOI: 10.1016/S2468-2667(20)30090-6] | Intervention |
| Cuesta_2020 | Cuesta J, Pico J. The Gendered Poverty Efects of the COVID‐19 Pandemic in Colombia. *Eur J Dev Res.*2020;32:1558–1591. [DOI: 10.1057/s41287-020-00328-2] | Intervention |
| Cuschieri_2020 | Cuschieri S, Pallari E, Hatziyianni A, et al. Dealing with COVID-19 in small European island states: Cyprus, Iceland and Malta. *Early Hum Dev*. 2020;Nov 12:105261. [DOI: 10.1016/j.earlhumdev.2020.105261] | Intervention |
| DallOlio_2020 | Dall’Olio R, Blacquiere T, Bouga M, et al. COLOSS survey: global impact of COVID-19 on bee research. *Journal of Apicultural Research*. 2020;59(5):731-734. [DOI: 10.1080/00218839.2020.1799646] | Intervention |
| Davalgi_2020 | Davalgi S, Undi M, Annadani R, et al. Comparison of Measures adopted to combat COVID 19 Pandemic by different countries in WHO regions. *Indian Journal of Community Health*. 2020;32(2). [DOI: 10.47203/IJCH.2020.v32i02SUPP.023] | Study type |
| Dertli_2020 | Dertli SE, Eryüzlü H. Early Warning Signals From Global Financial Markets at the Beginning of Covid -19 Pandemic. *Turkish Studies*. 2020;15(8):3507-3520. [DOI: 10.7827/TurkishStudies.45964] | Intervention |
| Doi_2020 | Doi H, Osawa T, Tsutsumida N. Global lockdown potential impact on achieving Sustainable Development Goals. *Earth ArXiv [Preprint]*. 2020. [DOI: 10.31223/X5F887] | Intervention |
| Doliwa-Klepacka_2020 | Doliwa-Klepacka A, Zdanowicz M. The European Union Current Asylum Policy: Selected Problems in the Shadow of COVID‐19. *Int J Semiot Law.* 2020. [DOI: 10.1007/s11196-020-09744-3] | Study type |
| Dollard_2020 | Dollard P, Griffin I, Berro A, et al. Risk Assessment and Management of COVID-19 Among Travelers Arriving at Designated U.S. Airports, January 17–September 13, 2020. *US Department of Health and Human Services/Centers for Disease Control and Prevention MMWR.*2020;69(45). | Outcome |

Appendix 6 (continued)

| **Study ID** | **Reference** | **Reason for exclusion** |
| --- | --- | --- |
| Doogan_2020 | Doogan C, Buntine W, Linger H, et al. Public Perceptions and Attitudes Toward COVID-19 Nonpharmaceutical Interventions Across Six Countries: A Topic Modeling Analysis of Twitter Data. *J Med Internet Res*. 2020;22(9):e21419). [DOI 10.2196/21419] | Outcome |
| Doraiswamy_2020 | Doraiswamy PM, Chilukuri M, Linares AR, et al. Are we ready for COVID-19’s Golden Passport? Insights from a Global Physician Survey. *medRxiv [Preprint]*. 2020. [DOI: 10.1101/2020.11.25.20234195] | Outcome |
| Dumka_2021 | Dumka UC, Kaskaoutis DG, Verma S, et al. Silver linings in the dark clouds of COVID-19: Improvement of air quality over India and Delhi metropolitan area from measurements and WRF-CHIMERE model simulations. *Atmospheric Pollution Research*. 2021;12(2):225-242. [DOI: 10.1016/j.apr.2020.11.005] | Intervention |
| Dvulit_2020 | Dvulit ZP, Danilyuk IA. ВПЛИВ COVID-19 НА РИНОК ВАНТАЖНИХ ЗАЛІЗНИЧНИХ ПЕРЕВЕЗЕНЬ [in Russian]. *УПРАВЛІННЯ НАЦІОНАЛЬНИМ ГОСПОДАРСТВОМ*. 2020;2(2). | Study type |
| Edwards_2020 | Edwards R, Sarhaddi-Blue L. A Closer Look at Retailer Resilience During the COVID-19 Pandemic. *The Secured Lender.*2020;Nov. | Study type |
| Ehrlichmann_2020 | Schröder J, Bhattacharjee D, Wittkamp N. Ready for check-in? How the travel industry can cope with COVID-19-triggered shifts in demand patterns. *McKinsey & Company - Travel, Logistics & Transport Infrastructure.*2020;July. | Intervention |
| EIA_2020 | U.S. Energy Information Administration (EIA). COVID-19 Mitigation Results in Lowest US Petroleum Consumption in Decades. 2020. | Intervention |
| Ejeromedoghene_2020 | Ejeromedoghene O, Tesi JN, Uyanga VA, et al. Food security and safety concerns in animal production and public health issues in Africa: A perspective of COVID-19 pandemic era. *Ethics Med Public Health*. 2020;15:100600. [DOI: 10.1016/j.jemep.2020.100600] | Study type |
| El Vally_2020 | El Vally A, Bollahi MA, Ahmedou MSO, et al. Retrospective overview of the coronavirus disease 2019 (COVID-19) outbreak in Mauritania. *New Microbes New Infect [PrePrint].*2020. [DOI: 10.1016/j.nmni.2020.100788] | Intervention |
| El Vally_2020_2 | El Vally A, Bollahi MA, Ahmedou MSO, et al. Retrospective overview of the coronavirus disease 2019 (COVID-19) outbreak in Mauritania. *New Microbes New Infect.*2020;38:100788. [DOI: 10.1016/j.nmni.2020.100788] | Duplicate |
| El Zowalaty_2020 | El Zowalaty ME, Young SG, Järhult JD. Environmental impact of the COVID-19 pandemic – a lesson for the future. *Infection Ecology & Epidemiology.*2020;10(1):1768023. [DOI: 10.1080/20008686.2020.1768023] | Study type |
| Elias_2020 | Elias B. Addressing COVID-19 Pandemic Impacts on Civil Aviation Operations. *Congressional Research Service*. 2020;Aug. | Study type |
| Erkhembayar_2020 | Erkhembayar R, Dickinson E, Badarch D, et al. Early policy actions and emergency response to the COVID-19 pandemic in Mongolia: experiences and challenges. *Lancet Glob Health*. 2020;8(9):e1234–41. [DOI: 10.1016/S2214-109X(20)30295-3 | Study type |
| Erokhin_2020 | Erokhin V, Gao T. Impacts of COVID-19 on Trade and Economic Aspects of Food Security: Evidence from 45 Developing Countries. *Int J Environ Res Public Health.*2020;17:5775. [DOI: 10.3390/ijerph17165775] | Intervention |

Appendix 6 (continued)

| **Study ID** | **Reference** | **Reason for exclusion** |
| --- | --- | --- |
| Espitia_2020 | Espitia A, Rocha N, Ruta M. Covid-19 and Food Protectionism. The Impact of the Pandemic and Export Restrictions on World Food Markets. *World Bank Group.*2020;May. | Study type |
| Falconer_2020 | Falconer R. Grown locally, harvested globally: The role of temporary foreign workers in Canadian agriculture. *The School of Public Policy Publications*. 2020;13. [DOI: 10.11575/sppp.v13i0.70510] | Study type |
| Faleiro_2020 | Faleiro S. The Trace Race. *MIT Technology Review*. 2020 | Study type |
| Falk_2020 | Falk MT, Hagsten E. The unwanted free rider: Covid-19. *Current Issues in Tourism.* 2020;May. [DOI: 10.1080/13683500.2020.1769575] | Intervention |
| Farhoudian_2020 | Farhoudian A, Radfar SR, Ardabili HM, et al. A global survey on changes in the supply, price and use of illicit drugs and alcohol, and related complications during the 2020 COVID-19 pandemic. *medRxiv [Preprint].*2020. [DOI: 10.1101/2020.07.16.20155341] | Intervention |
| Farrell_2020 | Farrell P, Thow AM, Wate JT, et al. COVID-19 and Pacific food system resilience: opportunities to build a robust response. *Food Sec.* 2020;12:783-791. [DOI: 10.1007/s12571-020-01087-y] | Study type |
| Feng_2020 | Feng HY. Crisis is Turning Point: Comprehensive Tourism Development for Domestic Tourism after COVID-19 [in Chinese]. *Journal of Tourism and Leisure Management.* 2020. [DOI: 10.6510/JTLM.202008/SP_8.0005] | Study type |
| Fiore_2020 | Fiore VG, DeFelice N, Glicksberg BS, et al. Containment of future waves of COVID-19: simulating the impact of different policies and testing capacities for contact tracing, testing, and isolation. *medRxiv [Preprint]*. 2020. [DOI: 10.1101/2020.06.05.20123372] | Intervention |
| Fouquet_2020 | Fouquet R, O’Garra T. The behavioural, welfare and environmental impacts of air travel reductions during and beyond COVID-19. *Centre for Climate Change Economics and Policy Working Paper No. 372*. 2020. ISSN 2515-5709 (Online) | Intervention |
| Gaffney_2020 | Gaffney C, Eeckels B. Covid-19 and Tourism Risk in the Americas. *Journal of Latin American Geography*. 2020;19(3):308-313. [DOI: 10.1353/lag.2020.0054] | Intervention |
| Garrett_2020 | Garrett TM. COVID-19, wall building, and the effects on Migrant Protection Protocols by the Trump administration: the spectacle of the worsening human rights disaster on the Mexico-U.S. border. *Administrative Theory & Praxis.* 2020. [DOI: 10.1080/10841806.2020.1750212] | Study type |
| Gibbs_2020 | Gibbs H, Liu Y, Pearson CAB, et al. Changing travel patterns in China during the early stages of the COVID-19 pandemic. *Nat Commun.*2020;11:5012. [DOI: 10.1038/s41467-020-18783-0] | Intervention |
| Godwin_2020 | Godwin A. The Contractual Impact of COVID-19 on Corporate and Financial Transactions. *Australian Business Law Review.*2020;48(2):116-125. | Study type |
| Goessling_2020 | Gössling S, Scott D, Hall M. Pandemics, tourism and global change: a rapid assessment of COVID-19. *Journal of Sustainable Tourism.*2021;29(1):1-20. [DOI: 10.1080/09669582.2020.1758708] | Study type |
| Goessling_2020_2 | Gössling S, Humpe A. The global scale, distribution and growth of aviation: Implications for climate change. *Global Environmental Change*. 2020;65:102194. [DOI: 10.1016/j.gloenvcha.2020.102194] | Non-Covid-19 |

Appendix 6 (continued)

| **Study ID** | **Reference** | **Reason for exclusion** |
| --- | --- | --- |
| Goodyear-Smith_2020 | Goodyear-Smith F, Kinder K, Mannie C, et al. Relationship between the perceived strength of countries’ primary care system and COVID-19 mortality: an international survey study. *BJGP Open.*2020;4(4). [DOI: 10.3399/bjgpopen20X101129] | Outcome |
| Goolsbee_2020 | Goolsbee A, Syverson C. Fear, lockdown, and diversion: Comparing drivers of pandemic economic decline 2020. *J Public Econ*. 2021;193:104311. [DOI: 10.1016/j.jpubeco.2020.104311] | Intervention |
| Gordon_2020 | Gordon DV, Grafton RQ, Steinshamn SI. Statistical Analyses of the Public Health and Economic Performance of Nordic Countries in Response to the COVID-19 Pandemic. *medRxiv [Preprint].* 2020. [DOI: 10.1101/2020.11.23.20236711] | Outcome |
| Govender_2020 | Govender K, Cowden RG, Nyamaruze P et al. Beyond the Disease: Contextualized Implications of the COVID-19 Pandemic for Children and Young People Living in Eastern and Southern Africa. *Front Public Health*. 2020;8:504. [DOI: 10.3389/fpubh.2020.00504] | Study type |
| Greenhalgh_2020 | Greenhalgh F, von Lingen AI, Cigan B, et al. Cross-border movement restrictions during Covid-19 and foreign nationals access to healthcare and medicines. *EATG Conference poster*. 2020. | Study type |
| GRID COVID-19 Study Group_2020 | GRID COVID-19 Study Group. Combating the COVID-19 pandemic in a resource-constrained setting: insights from initial response in India. *BMJ Global Health.*2020;5:e003416. [DOI: 10.1136/bmjgh-2020-003416] | Intervention |
| Gupta_2020 | Gupta A, Kunte R, Goyal N, et al. A comparative analysis of control measures on-board ship against COVID-19 and similar novel viral respiratory disease outbreak: Quarantine ship or disembark suspects? *Med J Armed Forces India*. 2020;Jun. [DOI: 10.1016/j.mjafi.2020.06.003] | Outcome |
| Habchi_2020 | Habchi K, Oulehri N, Noizet M, et al. Covid-19 : les transferts internationaux de patients de réanimation, une solution pour les départements frontaliers [in French]. *Ann Fr Med Urgence.*2020;10:298-305. [DOI: 10.3166/afmu-2020-0264] | Intervention |
| Han_2020 | Han E, Tan MMJ, Turk E, et al. Lessons learnt from easing COVID-19 restrictions: an analysis of countries and regions in Asia Pacific and Europe. *Lancet*. 2020;396:1525-1534. [DOI: 10.1016/S0140-6736(20)32007-9] | Study type |
| Haroon_2020 | Haroon O, Rizvi SAR. Flatten the Curve and Stock Market Liquidity – An Inquiry into Emerging Economies. *Emerging Markets Finance and Trade.*2020;56(10):2151-2161. [DOI: 10.1080/1540496X.2020.1784716] | Intervention |
| Hashim_2020 | Hashim BM, Al-Naseri SK, Al-Maliki A, et al. Impact of COVID-19 lockdown on NO2, O3, PM2.5 and PM10 concentrations and assessing air quality changes in Baghdad, Iraq. *Sci Total Environ*. 2021;754:141978. [DOI: 10.1016/j.scitotenv.2020.141978] | Intervention |
| Hennig_2020 | Hennig A. The spatial dimension of coronavirus crisis management and the role of subnational actors in the German–Polish border region. *European Societies*. 2020;Nov. [DOI: 10.1080/14616696.2020.1846065] | Outcome |
| Hoffmann_2020 | Hoffmann FF, Gonçalves I. Border Regimes and Pandemic Law in Time of COVID-19: A View from Brazil. *AJIL Unbound.*2020;114:327-331. [DOI: 10.1017/aju.2020.65] | Study type |

Appendix 6 (continued)

| **Study ID** | **Reference** | **Reason for exclusion** |
| --- | --- | --- |
| Hong_G_2020 | GuoHu H, Qing G, Qing M. Spread dynamics of SARS-CoV-2 epidemic in China: a phylogenetic analysis. *medRxiv [Preprint].* 2020. [DOI: 10.1101/2020.05.20.20107854] | Intervention |
| Hong_SY_2020 | Hong SY, Ashipala LSN, Bikinesi L. Rapid Adaption of HIV Treatment Programs in Response to COVID-19 - Namibia, 2020. *US Department of Health and Human Services/Centers for Disease Control and Prevention - MMWR*. 2020;69(42). | Study type |
| Hong_Y_2020 | Hong Y, Cai G, Mo Z, et al. The Impact of COVID-19 on Tourist Satisfaction with B&B in Zhejiang, China: An Importance–Performance Analysis. *Int J Environ Res Public Health*. 2020;17:3747. [DOI: 10.3390/ijerph17103747] | Intervention |
| Hoque_2020 | Hoque A, Shikha FA, Hasanat MW, et al. The Effect of Coronavirus (COVID-19) in the Tourism Industry in China. *Asian Journal of Multidisciplinary Studies.* 2020;3(1). | Study type |
| Hossain_2020 | Hossain ST. Impacts of COVID-19 on the Agri-food Sector: Food Security Policies of Asian Productivity Organization Members. *The Journal of Agricultural Sciences - Sri Lanka*. 2020;15(2):116-132. [DOI: 10.4038/jas.v15i2.8794] | Study type |
| Huang_2020 | Huang JK. Impacts of COVID-19 on agriculture and rural poverty in China. *Journal of Integrative Agriculture.*2020;19(12):2849–2853. [DOI: 10.1016/S2095-3119(20)63469-4] | Study type |
| Huang_QS_2020 | Huang QS, Wood T, Jelley L, et al. Impact of COVID-19 on nonpharmaceutical interventions on influenza and other respiratory viral infections in New Zealand. *medRxiv [Preprint]*. 2020. [DOI: 10.1101/2020.11.11.20228692] | Intervention |
| Hurford_2020 | Hurford A, Rahman P, Loredo-Osti JC. Modelling the impact of travel restrictions on COVID-19 cases in Newfoundland and Labrador. *medRxiv [Preprint].* 2020. [DOI: 10.1101/2020.09.02.20186874] | Outcome |
| Hussein_2020 | Hussein NR, Naqid IA, Saleem ZSM, et al. A sharp increase in the number of COVID-19 cases and case fatality rates after lifting the lockdown in Kurdistan region of Iraq. *Ann Med Surg.*2020;57:140-142. [DOI: 10.1016/j.amsu.2020.07.030] | Study type |
| Iacus_2020 | Iacus SM, Natale F, Santamaria C, et al. Estimating and Projecting Air Passenger Traffic during the COVID-19 Coronavirus Outbreak and its Socio-Economic Impact. *Safety Science*. 2020;129:104791. [DOI: 10.1016/j.ssci.2020.104791] | Intervention |
| Iacus_2020_2 | Iacus SM, Santamaria C, Sermi F, et al. Human mobility and COVID-19 initial dynamics. *Nonlinear Dyn*. 2020,101:1901-1919. [DOI: 10.1007/s11071-020-05854-6] | Intervention |
| Idler_2020 | Idler A, Hochmüller M. Covid-19 in Colombia's Borderlands and the Western Hemisphere: Adding Instability to a Double Crisis. *Journal of Latin American Geography*. 2020;19(3):280-288. [DOI: 10.1353/lag.2020.0050] | Study type |
| Iizuka_2020 | Iizuka N. Impact of the Coronavirus Pandemic on the Japanese Economy as Seen in the Overnight Travel Statistic Survey. *Japan Spotlight*. 2020;Sept. | Intervention |
| Ince_2020 | Ince E, Sürme M. A Qualitative Study on the Reflections of the Covid-19 Outbreak on Airline Companies [in Turkish]. *Journal of Social Sciences.*2020;Special Issue:571-584. [DOI: 10.21547/jss.788083] | Outcome |

Appendix 6 (continued)

| **Study ID** | **Reference** | **Reason for exclusion** |
| --- | --- | --- |
| Ivanov_2020 | Ivanov D. Predicting the impacts of epidemic outbreaks on global supply chains: A simulation-based analysis on the coronavirus outbreak (COVID-19/SARS-CoV-2) case. *Transportation Research Part E*. 2020;136:101922. [DOI: 10.1016/j.tre.2020.101922] | Intervention |
| Jaffe_2020 | Jaffe E, Sonkin R, Podolsky T, et al. The Role of Israel’s Emergency Medical Services During a Pandemic in the Pre-Exposure Period. *Disaster Med Public Health Prep.*2020;12:1-5. [DOI: 10.1017/dmp.2020.369] | Outcome |
| Jain_2020 | Jain S, Sharma T. Social and Travel Lockdown Impact Considering Coronavirus Disease (COVID-19) on Air Quality in Megacities of India: Present Benefits, Future Challenges and Way Forward. *Aerosol and Air Quality Research*. 2020;20:1222-1236. [DOI: 10.4209/aaqr.2020.04.0171] | Intervention |
| Janczuk_2020 | Janczuk M, Czapski G. The Impact of the Coronavirus Epidemic on Air Transport in Poland. *Postmodern Openings*. 2020;11(2):66-72. [DOI: 10.18662/po/11.2/159] | Study type |
| Janssens_2020 | Janssens W, Pradhan M, de Groot R, et al. The short-term economic effects of COVID-19 on low-income households in rural Kenya: An analysis using weekly financial household data. *World Development*. 2021;138:105280. [DOI: 10.1016/j.worlddev.2020.105280] | Study type |
| Jarratt_2020 | Jarratt D. An exploration of webcam-travel: Connecting to place and nature through webcams during the COVID-19 lockdown of 2020. *Tourism and Hospitality Research*. 2020;0(0):1–13. [DOI: 10.1177/1467358420963370] | Study type |
| Jephcote_2020 | Jephcote C, Hansell AL, Adams K, et al. Changes in air quality during COVID-19 ‘lockdown’ in the United Kingdom. *Environ Pollution*. 2020;272:116011. [DOI: 10.1016/j.envpol.2020.116011] | Study type |
| Johansson_2020 | Johansson MA, Wolford H, Paul P, et al. Reducing travel-related SARS-CoV-2 transmission with layered mitigation measures: Symptom monitoring, quarantine, and testing. *medRxiv [Preprint]*. 2020. [DOI: 10.1101/2020.11.23.20237412] | Outcome |
| Juganaru_2020 | Juganaru ID. Mass Tourism during the Coexistence with the New Coronavirus. The Predictable Evolution of the Seaside Tourism in Romania. *“Ovidius” University Annals, Economic Sciences Series*. 2020;0(1):171-179. | Study type |
| Kabir_2020 | Kabir M, Afzal MS, Khan A, et al. COVID-19 pandemic and economic cost; impact on forcibly displaced people. *Travel Med Infect Dis*. 2020;35:101661. [DOI: 10.1016/j.tmaid.2020.101661] | Study type |
| Kang_2020 | Kang M, Choi Y, Kim J, et al. COVID-19 impact on city and region: what’s next after lockdown? *International Journal of Urban Sciences*. 2020;24(3):297-315. [DOI: 10.1080/12265934.2020.1803107] | Study type |
| Karim_2020 | Karim N, Rybarczyk MM, Jacquet GA, et al. COVID-19 Pandemic Prompts a Paradigm Shift in Global Emergency Medicine: Multidirectional Education and Remote Collaboration. *AEM Education and Training.* 2020;5(1):79-90. [DOI: 10.1002/aet2.10551] | Intervention |
| Kashyap_2020 | Kashyap A, Raghuvanshi J. A preliminary study on exploring the critical success factors for developing COVID-19 preventive strategy with an economy centric approach. *Journal of the Iberoamerican Academy of Management*. 2020;18(4):357-377. [DOI: 10.1108/MRJIAM-06-2020-1046] | Study type |

Appendix 6 (continued)

| **Study ID** | **Reference** | **Reason for exclusion** |
| --- | --- | --- |
| Katirji_2020 | Katirji L, Smith L, Pelletier-Bui A, et al. Addressing Challenges in Obtaining Emergency Medicine Away Rotations and Standardized Letters of Evaluation Due to COVID-19 Pandemic. *West J Emerg Med*. 2020;21(3):538–541. [DOI: 10.5811/westjem.2020.3.47444] | Study type |
| Kendrick_2020 | Kendrick K, Isaac M. Mental health impact of COVID-19: Australian perspective. *Indian J Psychiatry*. 2020;62:S373-6. [DOI: 10.4103/psychiatry.IndianJPsychiatry_853_20] | Study type |
| Kenwick_2020 | Kenwick MR, Simmons BA. Pandemic Response as Border Politics. *International Organization.*2020;74(Supplement 2020):1-23. [DOI: 10.1017/S0020818320000363] | Outcome |
| Khanal_2020 | Khanal BP. Impact of the COVID-19 in Tourism Industry in Nepal and Policy Recommendation. *Journal of Tourism & Adventure.*2020;3(1):76-91. [DOI: 10.3126/jota.v3i1.31357] | Intervention |
| Kilic_2020 | Kilic B. The Effects of Pandemic COVID-19 on Turkish Tourism Industry [in Turkish]. *The Journal of Academic Social Science.*2020;8(105):254-262. [DOI: 10.29228/ASOS.43218] | Study type |
| King_2020 | King JA, Cabarkapa S, Leow FHP, et al. Addressing international student mental health during COVID-19: an imperative overdue. *Australas Psychiatry.*2020;28(4):469. [DOI: 10.1177/1039856220926934] | Study type |
| Kishore_2020 | Kishore N, Kahn R, Martinez PP, et al. Lockdown related travel behavior undermines the containment of SARS-CoV-2. *medRxiv [Preprint]*. 2020. [DOI: 10.1101/2020.10.22.20217752] | Intervention |
| Kluge_2020 | Kluge HHP, Jakab Z, Bartovic J, et al. Refugee and migrant health in the COVID-19 response. *The Lancet - Comment.*2020;395(10232):1237-1239. [DOI: 10.1016/S0140-6736(20)30791-1] | Study type |
| Knight_2020 | Knight DW, Xiong L. Impact of COVID-19: research note on tourism and hospitality sectors in the epicenter of Wuhan and Hubei Province, China.*International Journal of Contemporary Hospitality Management*. 2020;32(12):3705-3719. [DOI: 10.1108/IJCHM-04-2020-0278] | Intervention |
| Kortukova_2020 | Kortukova TO, Dei MO, Blahodarnyi AM, et al. COVID-19: Regulation of Migration Processes in The European Legal Area. *Cuestiones Políticas.*2020;38(Especial):321-332. [DOI: 10.46398/cuestpol.38e.29] | Study type |
| Korun_2020 | Korun O, Yurdakök O, Arslan A, et al. The impact of COVID‐19 pandemic on congenital heart surgery practice: An alarming change in demographics. *J Card Surg*. 2020;35(11):2908-2912. [DOI: 10.1111/jocs.14914] | Intervention |
| Kotnala_2020 | Kotnala G, Mandal TK, Sharma SK, et al. Emergence of Blue Sky Over Delhi Due to Coronavirus Disease (COVID‐19) Lockdown Implications. *Aerosol Sci Eng*. 2020;4:228–238. [DOI: 10.1007/s41810-020-00062-6] | Intervention |
| Krüger-Malpartida_2020 | Krüger-Malpartida H, Pedraz-Petrozzi B, Arevalo-Flores M, et al. Effects on Mental Health After the COVID-19 Lockdown Period: Results From a Population Survey Study in Lima, Peru. *Clinical Medicine Insights: Psychiatry*. 2020;11:1–9. [DOI: 10.1177/1179557320980423] | Intervention |
| Kuang_2020 | Kuang J, Ashraf S, Das U, et al. Awareness, Risk Perception, and Stress during the COVID-19 Pandemic in Communities of Tamil Nadu, India. *Int J Environ Res Public Health*. 2020;(17):7177. [DOI:10.3390/ijerph17197177] | Intervention |

Appendix 6 (continued)

| **Study ID** | **Reference** | **Reason for exclusion** |
| --- | --- | --- |
| Kuckertz_2020 | Kuckertz A, Brändle L, Gaudig A, et al. Startups in times of crisis – A rapid response to the COVID-19 pandemic. *Journal of Business Venturing Insights*. 2020;13:e00169. [DOI: 10.1016/j.jbvi.2020.e00169] | Intervention |
| Kulcsar_2020 | Kulcsar E. Comparative Analysis of Hungarian and Romanian Stock Market Indices in Context of COVID-19. *The Annals of the University of Oradea. Economic Sciences*. 2020;TOM XXIX(1). ISSN 1582-5450 (electronic format). | Intervention |
| Kumaran_2020 | Kumaran NK, Mahesh A, Sankar TK, et al. COVID-19 and international medical graduates: the frustrated and forgotten future NHS workforce. *Br J Hosp Med*. 2020. [DOI: 10.12968/hmed.2020.0397] | Study type |
| Kwok_2020 | Kwok KO. Letter to the Editor. Comparing the impact of various interventions to control the spread of COVID-19 in twelve countries. *The Journal of Hospital Infection*. 2020;106(1):214-216. [DOI: 10.1016/j.jhin.2020.06.029] | Intervention |
| Kwon_2020 | Kwon J, Grady C, Feliciano JT, et al. Defining facets of social distancing during the COVID-19 pandemic: Twitter analysis. *Journal of Biomedical Informatics.*2020;111:103601. [DOI: 10.1016/j.jbi.2020.103601] | Intervention |
| Lai_2020 | Lai IKW, Wong JWC. Comparing crisis management practices in the hotel industry between initial and pandemic stages of COVID-19. *International Journal of Contemporary Hospitality Management*. 2020;32(10):3135-3156. [DOI: 10.1108/IJCHM-04-2020-0325] | Intervention |
| Laila_2020 | Laila U, Sadiq N, Mehmood T, et al. Effects of Covid on Co2 Reduction and Climate Change. *Journal of Accounting and Finance in Emerging Economies*. 2020;6(4):1015-1020. | Study type |
| Laverty_2020 | Laverty AA, Millett C, Majeed A, et al. COVID-19 presents opportunities and threats to transport and health. *Journal of the Royal Society of Medicine.*2020;113(7):251–254. [DOI: 10.1177/0141076820938997] | Study type |
| Lazzarini_2020 | Lazzarini L, Barzon L, Foglia F, et al. First autochthonous dengue outbreak in Italy, August 2020. *Euro Surveill*. 2020;25(36):pii=20-01606. [DOI: 10.2807/1560-7917.ES.2020.25.36.2001606] | Study type |
| Le Quere_2020 | Le Quéré C, Jackson RB, Jones MW, et al. Temporary reduction in daily global CO2 emissions during the COVID-19 forced confinement. *Nature Climate Change*. 2020;10:647-653. [DOI: 10.1038/s41558-020-0797-x] | Intervention |
| Lee_2020 | Lee CC, Chen MP. The impact of COVID-19 on the travel and leisure industry returns: Some international evidence. *Tourism Economics.*2020:1-22. [DOI: 10.1177/1354816620971981] | Intervention |
| Lee_JN_2020 | Lee JN, Mahmud M, Morduch J, et al. Migration, Externalities, and the Diffusion of COVID-19 in South Asia. *Journal of Public Economics*. 2021;193:104312. [DOI: 10.1016/j.jpubeco.2020.104312] | Intervention |
| Leffler_2020 | Leffler CT, Ing E, Lykins JD, et al. Association of Country-wide Coronavirus Mortality with Demographics, Testing, Lockdowns, and Public Wearing of Masks. *Am J Trop Med Hyg.* 2020;00(0):1–12. [DOI: 10.4269/ajtmh.20-1015] | Outcome |
| Leiner_2020 | Leiner A, Sammon M, Perry H, et al. Facing COVID-19 and Refugee Camps on the U.S. Border. *J Emerg Med*. 2020;59(1):143–145. [DOI: 10.1016/j.jemermed.2020.04.041] | Study type |
| Leong_2020 | Leong WY. COVID-19’s impact on travel medicine surpasses that of all other emerging viral diseases. *J Travel Med*. 2020;27(8):taaa221. [DOI: 10.1093/jtm/taaa221] | Study type |

Appendix 6 (continued)

| **Study ID** | **Reference** | **Reason for exclusion** |
| --- | --- | --- |
| Lev_2020 | Lev D, Biber A, Lachish T, et al. Malaria in travellers in the time of corona. *J Travel Med.*2020;27(6):taaa067. [DOI: 10.1093/jtm/taaa067] | Study type |
| Li_Jiannan_2020 | Li J, Liang W, Yuan B, et al. Internalized Stigmatization, Social Support, and Individual Mental Health Problems in the Public Health Crisis. *Int J Environ Res Public Health*. 2020;17:507. [DOI: 10.3390/ijerph17124507] | Intervention |
| Li_Junxiong_2020 | Li J, Nguyen THH, Coca-Stefaniak JA. Coronavirus impacts on post-pandemic planned travel behaviours. *Annals of Tourism Research*. 2020:102964. [DOI: 10.1016/j.annals.2020.102964] | Intervention |
| Li_L_2020 | Li L, Li Q, Huang L, et al. Air quality changes during the COVID-19 lockdown over the Yangtze River Delta Region: An insight into the impact of human activity pattern changes on air pollution variation. *Sci Total Environ*. 2020;732:139282. [DOI: 10.1016/j.scitotenv.2020.139282] | Intervention |
| Liew_2020 | Liew VKS. The effect of novel coronavirus pandemic on tourism share prices. *Journal of Tourism Futures*. 2020. [DOI: 10.1108/JTF-03-2020-0045] | Intervention |
| Lim_2020 | Lim JT, Chew LZX, Choo ELW, et al. Increased dengue transmissions in Singapore attributable to SARS-CoV-2 social distancing measures. *J Infect Dis*. 2020;Sep 30:jiaa619. [DOI: 10.1093/infdis/jiaa619] | Intervention |
| Lindsey_2020 | Lindsey P, Allan J, Brehony P, et al. Conserving Africa’s wildlife and wildlands through the COVID-19 crisis and beyond. *Nat Ecol Evol*. 2020;4:1300–1310. [DOI: 10.1038/s41559-020-1275-6] | Study type |
| Liu_2020 | Liu Z, Ciais P, Deng Z, et al. Author correction: Near-real-time monitoring of global CO2 emissions reveals the effects of the COVID-19 pandemic. *Nature Communications*. 2020;11:6292. [DOI: 10.1038/s41467-020-20254-5] | Intervention |
| Lorenz_2020 | Lorenz C, Dias Bocewicz AC, Correa de Azevedo Marques C, Reis Santana LM, et al. Have measures against COVID-19 helped to reduce dengue cases in Brazil? *Travel Med Infect Dis*. 2020;37:101827. [DOI: 10.1016/j.tmaid.2020.101827] | Study type |
| Luo_2020 | Luo JM, Lam CF. Travel Anxiety, Risk Attitude and Travel Intentions towards “Travel Bubble” Destinations in Hong Kong: Effect of the Fear of COVID-19. *Int J Environ Res Public Health*. 2020;17:7859. [DOI: 10.3390/ijerph17217859] | Outcome |
| Ma_2020 | Ma S, Kim DD, Cohen JT, et al. Measuring “Fearonomic Effects” in Valuing Therapies: An Application to COVID-19 in China. *Value Health*. 2020;23(11):1405-1408. [DOI: 10.1016/j.jval.2020.06.002] | Intervention |
| Madani_2020 | Madani A, Boutebal SE, Benhamida H, et al. The Impact of Covid-19 Outbreak on the Tourism Needs of the Algerian Population. *Sustainability.*2020;12:8856. [DOI:10.3390/su12218856] | Intervention |
| Magalis_2020 | Magalis BR, Ramirez-Mata A, Zhukova A, et al. Differing impacts of global and regional responses on SARS-CoV-2 transmission cluster dynamics. *bioRxiv [Preprint]*. 2020. [DOI: 10.1101/2020.11.06.370999] | Outcome |
| Mahmassani_2020 | Mahmassani D, Tamim H, Makki M, et al. The impact of COVID-19 lockdown measures on ED visits in Lebanon. *Am J Emerg Med*. 2020. [DOI: 10.1016/j.ajem.2020.11.067] | Intervention |
| Mahmoud_2020 | Saleh FIM, Karia N. Benchmarks for INGOs’ effective responses during COVID-19 pandemic. *Benchmarking: An International Journal*. 2020. [DOI: 10.1108/BIJ-04-2020-0157] | Intervention |

Appendix 6 (continued)

| **Study ID** | **Reference** | **Reason for exclusion** |
| --- | --- | --- |
| Makurumidze_2020 | Makurumidze R. Coronavirus-19 disease (COVID-19): A case series of early suspected cases reported and the implications towards the response to the pandemic in Zimbabwe.*J Microbiol Immunol Infect.*2020;53(3):493-498. [DOI: 10.1016/j.jmii.2020.04.002] | Study type |
| Malagon-Rojas_2020 | Malagón-Rojas J, Parra B EL, Mercado M. Infection and Risk Perception of SARS-CoV-2 among Airport Workers: A Mixed Methods Study. *Int J Environ Res Public Health*. 2020;17:9002. [DOI: 10.3390/ijerph17239002] | Intervention |
| Malmberg_2020 | Malmberg H, Britton T. Inflow restrictions can prevent epidemics when contact tracing efforts are effective but have limited capacity. *J R Soc Interface*. 2020;17:20200351. [DOI: 10.1098/rsif.2020.0351] | Outcome |
| Marshall_2020 | Marshall M. Changing the way we use the roads. *Focus Feature COVID-19*. 2020. | Study type |
| McCarthy_2020 | McCarthy C, Carayannopoulos K, Walton JM. COVID-19 and changes to postgraduate medical education in Canada. *CMAJ*. 2020;192(35):e1018-1020. [DOI: 10.1503/cmaj.200882] | Study type |
| McCartney_2020 | McCartney G. The impact of the coronavirus outbreak on Macao. From tourism lockdown to tourism recovery. *Current Issues in Tourism*. 2020. [DOI: 10.1080/13683500.2020.1762549] | Study type |
| McKibbin_2020 | McKibbin W, Fernando R. The Global Macroeconomic Impacts of COVID-19: Seven Scenarios. CAMA Working Paper 19/2020. *SSRN [Preprint].*2020. | Intervention |
| McMaster_2020 | McMaster D, Veremu M, Jonas KM. Should international medical electives to resource-poor countries continue during COVID-19? *J Travel Med*. 2020;27(6):taaa071. [DOI: 10.1093/jtm/taaa071] | Study type |
| Michaelowa_2020 | Michaelowa A, Poralla M, Kessler J, et al. COVID-19 impacts on developing countries and links between reco- very policies, climate change miti- gation and international carbon markets. CIS Working Paper No.106. *Center for Comparative and International Studies (CIS)*. 2020. | Study type |
| Milman_2020 | Milman E, Lee SA, Neimeyer RA. Social isolation as a means of reducing dysfunctional coronavirus anxiety and increasing psychoneuroimmunity. *Brain, Behaviour, and Immunity.*2020;87:138-139. [DOI: 10.1016/j.bbi.2020.05.007] | Intervention |
| Milman_2020_2 | Milman E, Lee SA, Neimeyer RA. Social isolation and the mitigation of coronavirus anxiety: The mediating role of meaning. *Death Studies*. 2020. [DOI: 10.1080/07481187.2020.1775362] | Intervention |
| Miyazaki_2020 | Miyazaki K, Bowman K, Sekiya T, et al. Air Quality Response in China Linked to the 2019 Novel Coronavirus (COVID-19) Lockdown. *Geophysical Research Letters*. 2020;47:e2020GL089252. [DOI: 10.1029/2020GL089252] | Intervention |
| Miyazaki_2020_2 | Miyazaki K, Bowman K, Sekiya T, et al. Global tropospheric ozone responses to reduced NOx emissions linked to the COVID-19 world-wide lockdowns. *Earth and Space Science Open Archive [Preprint].*2020. [DOI: 10.1002/essoar.10504795.1] | Intervention |
| Modini_2020 | Modini M, Vrklevski L. A hotel room on Mars: quarantine and the psychological view from the virtual front line. *Australasian Psychiatry*. 2020;28(6):624–626. [DOI: 10.1177/1039856220956465] | Study type |
| Mohammed_2020 | Mohammed H, Oljira L, Roba KT, et al. Containment of COVID-19 in Ethiopia and implications for tuberculosis care and research. *Infectious Diseases of Poverty*. 2020;9:131. [DOI: 10.1186/s40249-020-00753-9] | Study type |

Appendix 6 (continued)

| **Study ID** | **Reference** | **Reason for exclusion** |
| --- | --- | --- |
| Monmousseau_2020 | Monmousseau P, Marzuoli A, Feron E, et al. Impact of Covid-19 on passengers and airlines from passenger measurements: Managing customer satisfaction while putting the US Air Transportation System to sleep. *Transportation Research Interdisciplinary Perspectives*. 2020;7:100179. [DOI: 10.1016/j.trip.2020.100179] | Outcome |
| Montano_2020 | Montano W, Gushiken E. Lima soundscape before confinement and during curfew. Airplane flights suppressions because of Peruvian lockdown. *The Journal of the Acoustical Society of America.*2020;148:1824. [DOI: 10.1121/10.0002112] | Intervention |
| Muhammad_2020 | Muhammad S, Long X, Salman M. COVID-19 pandemic and environmental pollution: A blessing in disguise? *Sci Total Environ.*2020;728:138820. [DOI: 10.1016/j.scitotenv.2020.138820] | Intervention |
| Mukherjee_2020 | Mukherjee D. COVID-19 and South Asian Tourism: Challenging Times Ahead. *Institute of South Asian Studies - ISAS Briefs*. 2020(786). | Study type |
| Murakami_2020 | Murakami E, Shimizutani S, Yamada E. Projection of the Effects of the COVID-19 Pandemic on the Welfare of Remittance-Dependent Households in the Philippines. *Economics of Disasters and Climate Change*. 2020. [DOI: 10.1007/s41885-020-00078-9] | Intervention |
| Mushayabasa_2020 | Mushaybasa S, Ngarakana-Gwasira ET, Mushanyu J. On the role of governmental action and individual reaction on COVID-19 dynamics in South Africa: A mathematical modelling study. *Informatics in Medicine Unlocked*. 2020;20:100387. [DOI: 10.1016/j.imu.2020.100387] | Intervention |
| Myers_2020 | Myers JF, Snyder RE, Porse CC, et al. Identification and Monitoring of International Travelers During the Initial Phase of an Outbreak of COVID-19 — California, February 3–March 17, 2020. *US Department of Health and Human Services/Centers for Disease Control and Prevention - MMWR*. 2020;69(19):599-602. | Study type |
| Neuburger_2020 | Neuburger L, Egger R. Travel risk perception and travel behaviour during the COVID-19 pandemic 2020: a case study of the DACH region. *Current Issues in Tourism*. 2020. [DOI: 10.1080/13683500.2020.1803807] | Intervention |
| Ni Ghrainne_2020 | Ní Ghráinne B. COVID-19, Border Closures, and International Law. *SSRN [Preprint]*. 2020. [DOI: 10.2139/ssrn.3662218] | Study type |
| Nicola_2020 | Nicola M, Alsafi Z, Sohrabi C, et al. The socio-economic implications of the coronavirus pandemic (COVID-19): A review. *Int J Surg.*2020;78:185-193. [DOI: 10.1016/j.ijsu.2020.04.018] | Study type |
| Niu_2020 | Niu R, Wong EWM, Chan YC, et al. Modeling the COVID-19 Pandemic Using an SEIHR Model With Human Migration. *IEEE*. 2020;8:195503-195514. [DOI: 10.1109/ACCESS.2020.3032584] | Outcome |
| Nolen_2020 | Nolen LD, Seeman S, Bruden D, et al. Impact of Social Distancing and Travel Restrictions on Non–Coronavirus Disease 2019 (Non–COVID-19) Respiratory Hospital Admissions in Young Children in Rural Alaska. *Clin Infect Dis*. 2020;Sep 5:ciaa1328. [DOI: 10.1093/cid/ciaa1328] | Intervention |
| OBrien_2020 | O’Brien M, Eger MA. Suppression, Spikes, and Stigma: How COVID-19 Will Shape International Migration and Hostilities toward It. *International Migration Review*. 2020. [DOI: 10.1177/0197918320968754] | Study type |
| Oruonye_2020 | Oruonye ED, Ahmed YM. An Appraisal of the Potential Impacts of Covid-19 on Tourism in Nigeria. *Journal of Economics and Technology Research.*2020;1(1):32-41. [DOI: 10.22158/jetr.v1n1p32] | Study type |

Appendix 6 (continued)

| **Study ID** | **Reference** | **Reason for exclusion** |
| --- | --- | --- |
| Otmani_2020 | Otmani A, Benchrif A, Tahri M, et al. Impact of Covid-19 lockdown on PM10, SO2 and NO2 concentrations in Salé City (Morocco). *Sci Total Environ.*2020;735:139541. [DOI: 10.1016/j.scitotenv.2020.139541] | Intervention |
| Ou_2020 | Ou S, He X, Ji W, et al. Machine learning model to project the impact of COVID-19 on US motor gasoline demand. *Nature Energy*. 2020;5:666-673. [DOI: 10.1038/s41560-020-0662-1] | Intervention |
| Oum_2020 | Oum TH, Wang K. Socially optimal lockdown and travel restrictions for fighting communicable virus including COVID-19. *Transport Policy*. 2020;96:94-100. [DOI: 10.1016/j.tranpol.2020.07.003] | Intervention |
| Ozdemir_2020 | Ozdemir MA. What are the Economic, Psychological and Social Consequences of the Covid-19 Crisis on Tourism Employees? *International Journal of Social, Political and Economic Research*. 2020;7(4):1137-1163. [DOI: 10.46291/IJOSPERvol7iss4pp1137-1163] | Intervention |
| Pan_2020 | Pan S. COVID-19 and the neo-liberal paradigm in higher education: changing landscape. *Asian Education and Development Studies*. 2020. [DOI: 10.1108/AEDS-06-2020-0129] | Study type |
| Pan-ngum_2020 | Pan-ngum W, Poomchaichote T, Cuman G, et al. Social, ethical and behavioural aspects of COVID-19. *Wellcome Open Res*. 2020;5:90. [DOI: 10.12688/wellcomeopenres.15813.2] | Study type |
| Papadopoulos_2020 | Papadopoulos DI, Donkov I, Charitopoulos K, et al. The impact of lockdown measures on COVID-19: a worldwide comparison. *medRxiv [Prerprint].*2020. [DOI: 10.1101/2020.05.22.20106476] | Outcome |
| Park_2020 | Park J, Rhim HC. Consequences of coronavirus disease 2019 on international medical graduates and students applying to residencies in the United States. *Korean J Med Educ*. 2020;32(2): 91-95. [DOI: 10.3946/kjme.2020.156] | Study type |
| Petetin_2020 | Petetin H, Bowdalo D, Soret A, et al. Meteorology-normalized impact of COVID-19 lockdown upon NO2 pollution in Spain. *Atmos Chem Phys*. 2020;20:11119-11141. [DOI: 10.5194/acp-20-11119-2020] | Intervention |
| Phan_2020 | Phan DHB, Narayan PK. Country Responses and the Reaction of the Stock Market to COVID-19—a Preliminary Exposition. *Emerging Markets Finance and Trade.*2020;56(10):2138-2150. [DOI: 10.1080/1540496X.2020.1784719] | Study type |
| Popa_2020 | Popa IC, Bosinceanu A. Restarting the Air Transport Industry After Covid-19 – An Economic Forecast. *“Ovidius” University Annals, Economic Sciences Series.*2020;0(1):451-456. | Intervention |
| Potapova_2020 | Potapova AA. РИСК ОГРАНИЧЕНИЯ ЗАНЯТОСТИ ИНОСТРАННЫХ МИГРАНТОВ В СЕЛЬСКОМ ХОЗЯЙСТВЕ РОССИИ В УСЛОВИЯХ ПАНДЕМИИ [in Russian]. *ЭКОНОМИЧЕСКОЕ РАЗВИТИЕ РОССИИ.*2020;17(6):44-53. | Study type |
| Poudel_2020 | Poudel K, Subedi P. Impact of COVID-19 pandemic on socioeconomic and mental health aspects in Nepal. *Int Journal Soc Psychiatry*. 2020;66(8):748–755. [DOI: 10.1177/0020764020942247] | Study type |
| PracticeNurse_2020 | Anonymous. Nursing number grow. *Practice Nurse*. 2020;50(9):8. | Study type |

Appendix 6 (continued)

| **Study ID** | **Reference** | **Reason for exclusion** |
| --- | --- | --- |
| Prinja_2020 | Prinja S, Bahuguna P, Chugh Y. A Model Based Analysis for COVID-19 Pandemic in India: Implications for Health Systems and Policy for Low- and Middle-Income Countries. *medRxiv [Preprint].* 2020. [DOI: 10.1101/2020.06.11.20128231] | Outcome |
| Provian_2020 | Provian C. Taxation of posted workers during the COVID-19 pandemic [in Romanian]. *Tax Magazine*. 2020;3:174-176. | Study type |
| Qiu_2020 | Qui RTR, Park J, Li S, et al. Social costs of tourism during the COVID-19 pandemic. *Ann Tour Res*. 2020;84:102994. [DOI: 10.1016/j.annals.2020.102994] | Intervention |
| Quarantine Management Team_2020 | Quarantine Management Team, COVID-19 National Emergency Response Center. Coronavirus Disease-19: Quarantine Framework for Travelers Entering Korea. *Osong Public Health Res Perspect.*2020;11(3):133-139. [DOI: 10.24171/j.phrp.2020.11.3.04] | Study type |
| Radu_2020 | Radu BM. Analysis of the different measures taken by the European Union and Romania to contract the effects of COVID on th labor market. *Internal Auditing & Risk Management*. 2020;2(58):9-21. [DOI: 10.5281/zenodo.3923201] | Study type |
| Rajput_2020 | Rajput H, Changotra R, Rajput P, et al. A shock like no other: coronavirus rattles commodity markets. *Environment, Development and Sustainability.* 2020. [DOI: 10.1007/s10668-020-00934-4] | Study type |
| Ram_2020 | Ram SK, Sornette D. Impact of Governmental interventions on epidemic progression and workplace activity during the COVID-19 outbreak. *Swiss Finance Institute Research Paper [Preprint]*. 2020. [DOI: 10.2139/ssrn.3619202] | Outcome |
| Ramji-Nogales_2020 | Ramji-Nogales J, Goldner Lang I. Freedom of movement, migration, and borders. *Journal of Human Rights*. 2020,19(5):593-602. [DOI: 10.1080/14754835.2020.1830045] | Study type |
| Rapaccini_2020 | Rapaccini M, Saccani N, Kowalkowski C, et al. Navigating disruptive crises through service-led growth: The impact of COVID-19 on Italian manufacturing firms. *Industrial Marketing Management*. 2020;88:225-237. [DOI: 10.1016/j.indmarman.2020.05.017] | Intervention |
| Ravaldi_2020 | Ravaldi C, Ricca V, Wilson A, et al. Previous psychopathology predicted severe COVID-19 concern, anxiety, and PTSD symptoms in pregnant women during “lockdown” in Italy. *Arch Womens Ment Health*. 2020;23:783–786. [DOI: 10.1007/s00737-020-01086-0] | Intervention |
| Reis_2020 | Reis RF, de Melo Quintela B, de Oliveira Campos J, et al. Characterization of the COVID-19 pandemic and the impact of uncertainties, mitigation strategies, and underreporting of cases in South Korea, Italy, and Brazil. *Chaos, Solitons & Fractals*. 2020;136:109888. [DOI: 10.1016/j.chaos.2020.109888] | Outcome |
| Rios-Gonzalez_2020 | Rios-González CM. Knowledge, Attitudes, and Practices towards COVID-19 in Paraguayans During the Outbreak Period: A Quick Online Survey [in Spanish]. *Rev Salud Publica Parag*. 2020;10(2):17-22. [DOI: 10.18004/rspp.2020.diciembre.17] | Intervention |
| Ritish_2020 | Ritish D, Dinakaran D, Chander R, et al. Letter to the Editor: Mental health concerns in quarantined international air passengers during COVID-19 pandemic – An experiential account. *Asian Journal of Psychiatry*. 2020;53:102364. [DOI: 10.1016/j.ajp.2020.102364] | Intervention |

Appendix 6 (continued)

| **Study ID** | **Reference** | **Reason for exclusion** |
| --- | --- | --- |
| Rivera Garcia_2020 | Rivera García J. Towards a more sustainable tourism after COVID-19? Perception of Spanish travel agencies [in Spanish]. *Gran Tour: Revista de Investigaciones Turísticas*. 2020;21:206-229. | Intervention |
| Roberto_2020 | Roberto KJ, Johnson AF, Rauhaus BM. Stigmatization and prejudice during the COVID-19 pandemic. *Administrative Theory & Praxis*. 202; 42(3): 364-378. [DOI: 10.1080/10841806.2020.1782128] | Study type |
| Rogerson_2020 | Rogerson CM, Rogerson JM. COVID-19 and Tourism Spaces of Vulnerability in South Africa. *African Journal of Hospitality, Tourism and Leisure*. 2020;9(4):382-401. [DOI: 10.46222/ajhtl.19770720-26] | Intervention |
| Ropkins_2021 | Ropkins K, Tate JE. Early observations on the impact of the COVID-19 lockdown on air quality trends across the UK. *Sci Total Environ*. 2021;754:142374. [DOI: 10.1016/j.scitotenv.2020.142374] | Intervention |
| Roy_2020 | Roy D, Tripathy S, Kar SK, et al. Study of knowledge, attitude, anxiety & perceived mental healthcare need in Indian population during COVID-19 pandemic. *Asian J Psychiatr.*2020;51:102083. [DOI: 10.1016/j.ajp.2020.102083] | Intervention |
| Rubbaniy_2020 | Rubbaniy G, Khalid AA, Umar M, et al. European Stock Markets’ Response to Covid-19, Lockdowns, Government Response Stringency and Central Banks’ Interventions. *SSRN [Preprint]*. 2020. | Intervention |
| Ruiz Estrada_2020 | Ruiz Estrada MA, Park D, Lee M. How A Massive Contagious Infectious Diseases can Affect Tourism, International Trade, Air Transportation, and Electricity Consumption? The Case of 2019 novel coronavirus (2019-nCoV) in China. *SSRN [Preprint]*. 2020. [DOI: 10.2139/ssrn.3540667] | Intervention |
| Rutayisire_2020 | Rutayisire E, Nkundimana G, Mitonga HK, et al. What works and what does not work in response to COVID-19 prevention and control in Africa. *Int J Infect Dis.* 2020;97:267–269. [DOI: 10.1016/j.ijid.2020.06.024] | Study type |
| Sabat_2020 | Sabat I, Neuman-Böhme S, Varghese NE, et al. United but divided: Policy responses and people’s perceptions in the EU during the COVID-19 outbreak. *Health Policy*. 2020;124:909-918. [DOI: 10.1016/j.healthpol.2020.06.009] | Outcome |
| Sah_2020 | Sah R, Sigdel S, Ozaki A, et al. Impact of COVID-19 on tourism in Nepal. *J Travel Med*. 2020;27(6):taaa105. [DOI: 10.1093/jtm/taaa105] | Study type |
| Sahin_2020 | Sahin ÜA. The Effects of COVID-19 Measures on Air Pollutant Concentrations at Urban and Traffic Sites in Istanbul. *Aerosol and Air Qual Res.*2020;20: 1874–1885. [DOI: 10.4209/aaqr.2020.05.0239] | Intervention |
| Sahu_2020 | Sahu A, Naqvi WM. Floating countries and corona pandemic: Impact of COVID-19 on stranded Cruise ships. Sahu, *Int J Res Pharm Sci*. 2020;11(SPL)(1):219-223. [DOI: 10.26452/ijrps.v11iSPL1.2702] | Study type |
| Santana Cibrian_2020 | Santana-Cibrian M, Acuna-Zegarra MA, Velasco-Hernandez JX. Lifting mobility restrictions and the effect of superspreading events on the short-term dynamics of COVID-19. *Math Biosci Eng*. 2020;17(5):6240-6258. [DOI: 10.3934/mbe.2020330] | Intervention |
| Santos_2020 | Santos J. Refections on the impact of “fatten the curve” on interdependent workforce sectors. *Environ Syst Decis.*2020;40:185–188. [DOI: 10.1007/s10669-020-09774-z] | Study type |
| Sarfraz_2020 | Sarfraz M, Shehzad K, Shah SGM. The impact of COVID-19 as a necessary evil on air pollution in India during the lockdown.*Environmental Pollution.*2020;266:115080. [DOI: 10.1016/j.envpol.2020.115080] | Intervention |

Appendix 6 (continued)

| **Study ID** | **Reference** | **Reason for exclusion** |
| --- | --- | --- |
| Sarrica_2020 | Sarrica F, Healy C, Serio G, et al. How COVID-19 restrictions and the economic consequences are likely to impact migrant smuggling and cross-border trafficking in persons to Europe and North America. *UNODC Research and Trend Analysis Branch*. 2020. | Study type |
| Saslavsky_2020 | Saslavsky D, Rastogi C. Facilitating Air Freight - Policies and Actions. *World Bank Group*. 2020;May. | Study type |
| Schlichtiger_2020 | Schlichtiger J, Brunner S, Steffen J, et al. Mental health impairment triggered by the COVID-19 pandemic in a sample population of German students. *J Investig Med.*2020;68:1394–1396. [DOI: 10.1136/jim-2020-001553] | Intervention |
| Schlosser_2020 | Schlosser F, Maier BF, Jack O, et al. COVID-19 lockdown induces disease-mitigating structural changes in mobility networks. *PNAS*. 2020;117(52):32883-32890. [DOI: 10.1073/pnas.2012326117] | Intervention |
| Seleiman_2020 | Seleiman MF, Selim S, Alhammad BA, et al. Will novel Coronavirus (COVID-19) pandemic impact agriculture, food security and animal sectors? *Biosc J*. 2020;36(4):1315-1326. [DOI: 10.14393/BJ-v36n4a2020-54560] | Study type |
| Serrano_2020 | Serrano F, Kazda A. The future of airport post COVID-19. *J Air Transp Manag.*2020;89:101900. [DOI: 10.1016/j.jairtraman.2020.101900] | Study type |
| Seyfi_2020 | Seyfi S, Hall CM, Shabani B. COVID-19 and international travel restrictions: the geopolitics of health and tourism. *Tourism Geographics*. 2020. [DOI: 10.1080/14616688.2020.1833972] | Study type |
| Shan_2020 | Shan Y, Ou J, Wang D, et al. Impacts of COVID-19 and fiscal stimuli on global emissions and the Paris Agreement. *Nat Clim Chang*. 2020. [DOI: 10.1038/s41558-020-00977-5] | Intervention |
| Shanaev_2020 | Shanaev S, Shuraeva A, Ghimire B. The Financial Pandemic: COVID-19 and Policy Interventions on Rational and Irrational Markets. *SSRN [Preprint].* 2020. [DOI: 10.2139/ssrn.3589557] | Intervention |
| Sharma_A_2020 | Sharma A, Nicolau JL. An open market valuation of the effects of COVID-19 on the travel and tourism industry. *Ann Tour Res*. 2020;83:102990. [DOI: 10.1016/j.annals.2020.102990\| | Intervention |
| Sharma_H_2020 | Sharma H, Verma S. Preservation of physical and mental health amid COVID-19 pandemic: Recommendations from the existing evidence of disease outbreaks. *IJAM*. 2020;6(2):76-82. [DOI: 10.4103/IJAM.IJAM_47_20] | Study type |
| Shimizu_2020 | Shimizu K, Negita M. Lessons Learned from Japan’s Response to the First Wave of COVID-19: A Content Analysis. *Healthcare*. 2020;8:426. [DOI: doi:10.3390/healthcare8040426] | Intervention |
| Shrestha_2020 | Shrestha N, Shad MY, Ulvi O, et al. The impact of COVID-19 on globalization. *One Health.*2020;11:100180. [DOI: 10.1016/j.onehlt.2020.100180] | Intervention |
| Sicard_2020 | Sicard P, de Marco A, Agathokleous E, et al. Amplified ozone pollution in cities during the COVID-19 lockdown. *Sci Total Environ.*2020;735:139532. [DOI: 10.1016/j.scitotenv.2020.139542] | Intervention |
| Siche_2020 | Siche R. What is the impact of COVID-19 disease on agriculture? *Scientia Agropecuaria*. 2020;11(1):3-6. [DOI: 10.17268/sci.agropecu.2020.01.00] | Study type |

Appendix 6 (continued)

| **Study ID** | **Reference** | **Reason for exclusion** |
| --- | --- | --- |
| Siddique_2021 | Siddique A, Shahzad A, Lawler J, et al. Unprecedented environmental and energy impacts and challenges of COVID-19 pandemic. *Environ Res*. 2021;193:110443. [DOI: 10.1016/j.envres.2020.110443] | Study type |
| Singh_MK_2020 | Singh MK, Neog Y. Contagion effect of COVID-19 outbreak: Another recipe for disaster on Indian economy. *J Public Affairs*. 2020;20:e2171. [DOI: 10.1002/pa.2171] | Study type |
| Singh_RP_2020 | Singh RP, Chauhan A. Impact of lockdown on air quality in India during COVID-19 pandemic. *Air Quality, Atmosphere & Health*. 2020;13:921–928. [DOI: 10.1007/s11869-020-00863-1] | Intervention |
| Singh_S_2020 | Singh S, Kumar R, Panchal R, et al. Impact of COVID-19 on logistics systems and disruptions in food supply chain. *International Journal of Production Research*. 2020. [DOI: 10.1080/00207543.2020.1792000] | Intervention |
| Skare_2020 | Skare M, Soriano DR, Porada-Rochon M. Impact of COVID-19 on the travel and tourism industry. *Technological Forecasting and Social Change.*2020;163:120469. [DOI: 10.1016/j.techfore.2020.120469] | Intervention |
| Sobieralski_2020 | Sobieralski JB. COVID-19 and airline employment: Insights from historical uncertainty shocks to the industry. *Transportation Research Interdisciplinary Perspectives*. 2020;5:100123. [DOI: 10.1016/j.trip.2020.100123] | Intervention |
| Sobieralski_2020_2 | Sobieralski JB, Hubbard SM. The Effect of Jet Fuel Tax Changes on Air Transport, Employment, and the Environment in the US. *Sustainability*. 2020;12:3352. [DOI: 10.3390/su12083352] | Non-Covid-19 |
| Soehardi_2020 | Soehardi S, Purnamaasih L, Rapitasari D. Dampak Pandemik Covid-19 Terhadap Kunjungan Turis Asing dan Domestik serta Tingkat Hunian Kamar Hotel Bintang di Indonesia [in Indonesian]. *Jurnal Kajian Ilmiah (JKI)*. 2020;20(3):291-308. [DOI: 10.31599/jki.v20i3.287] | Intervention |
| Song_2020 | Song KH, Choi S. A Study on the Behavioral Change of Passengers on Sustainable Air Transport after COVID-19. *Sustainability*. 2020;12:9207. [DOI: 10.3390/su12219207] | Outcome |
| Sönmez_2020 | Sönmez S, Apostolopoulos Y, Lemke MK, et al. Understanding the effects of COVID-19 on the health and safety of immigrant hospitality workers in the United States. *Tour Manag Perspec*t. 2020;35:100717. [DOI: 10.1016/j.tmp.2020.100717] | Study type |
| Sotomayor-Castillo_2020 | Sotomayor-Castillo C, Radford K, Li C, et al. Air travel in a COVID-19 world: Commercial airline passengers’ health concerns and attitudes towards infection prevention and disease control measures. *Infection, Disease & Health*. 2020. [DOI: 10.1016/j.idh.2020.11.002] | Intervention |
| Spelta_2020 | Spelta A, Flori A, Pierri F, et al. After the lockdown: simulating mobility, public health and economic recovery scenarios. *Sci Rep*. 2020;10:16950. [DOI: 10.1038/s41598-020-73949-6] | Intervention |
| Stangeland_2020 | Stangeland B. How to evaluate the success of the COVID-19 measures implemented by the Norwegian government by analyzing changes in doubling time. *medRxiv [Preprint]*. 2020. [DOI: 10.1101/2020.03.29.20045187] | Intervention |
| Steffen_2020 | Steffen R, Lautenschlager S, Fehr J. Travel restrictions and lockdown during the COVID-19 pandemic — impact on notified infectious diseases in Switzerland.  *J Travel Med*. 2020;27(8):taaa180. [DOI: 10.1093/jtm/taaa180] | Intervention |

Appendix 6 (continued)

| **Study ID** | **Reference** | **Reason for exclusion** |
| --- | --- | --- |
| Steyn_2020 | Steyn N, Binny RN, Hendy SC, et al. The effect of border controls on the risk of COVID-19 reincursion from international arrivals. *medRxiv [Preprint]*. 2020. [DOI: 10.1101/2020.07.15.20154955] | Outcome |
| Streimikiene_2020 | Streimikiene D, Korneeva E. Economic impacts of innovation in tourism marketing. *Terra Economicus*. 2020;18(3):182-193. [DOI: 10.18522/2073-6606-2020-18-3-182-193] | Study type |
| Sudipta_2020 | Sudipta A, Kaushik B. The Impact of Coronavirus into the Global Economic Prospects. *ITIHAS The Journal of Indian Management.*2020. | Study type |
| Sugiura_2020 | Sugiura K, Kure K, Kato T, et al. Change in the ASF entry risk into Japan as a result of the COVID-19 pandemic. *Transbound Emerg Dis*. 2020;00:1–4. [DOI: 10.1111/tbed.13836] | Intervention |
| Summan_2020 | Summan A, Nandi A. Timing of non-pharmaceutical interventions to mitigate COVID-19 transmission and their effects on mobility: A cross-country analysis. *medRxiv [Preprint].*2020. [DOI: 10.1101/2020.05.09.20096420] | Intervention |
| Sun_2020 | Sun X, Wandelt S, Zhang A. How did COVID-19 impact air transportation? A first peek through the lens of complex networks. *J Air Transp Manag*. 2020;89:101928. [DOI: 10.1016/j.jairtraman.2020.101928] | Intervention |
| Suresh_2020 | Rajani S, Justine J, Balraju RSj. Migrant Workers at Crossroads–The Covid-19 Pandemic and the Migrant Experience in India. *Social Work in Public Health*. 2020;35(7):633-643. [DOI: 10.1080/19371918.2020.1808552] | Study type |
| Sy_2020 | Sy C, Bernardo E, Miguel A, et al. Policy Development for Pandemic Response Using System Dynamics: a Case Study on COVID-19. *Process Integration and Optimization for Sustainability*. 2020;4:497-501. [DOI: 10.1007/s41660-020-00130-x] | Study type |
| Tanrivermis_2020 | Tanrivermis H. Possible impacts of COVID-19 outbreak on real estate sector and possible changes to adopt: A situation analysis and general assessment on Turkish perspective. *Journal of Urban Management*. 2020;9:263-269. [DOI: 10.1016/j.jum.2020.08.005] | Study type |
| Tatum_2020 | Tatum M. Will medical tourism survive covid-19? *BMJ*. 2020;370:m2677. [DOI: 10.1136/bmj.m2677] | Study type |
| Taylor_2020 | Taylor RA, McCarthy C, Patel V, et al. The risk of introducing SARS-CoV-2 to the UK via international travel in August 2020. *medRxiv [Preprint]*. 2020. [DOI: 10.1101/2020.09.09.20190454] | Outcome |
| Tohjima_2020 | Tohjima Y, Patra PK, Niwa Y, et al. Detection of fossil‐fuel CO2 plummet in China due to COVID‐19 by observation at Hateruma. *Sci Rep*. 2020;10:18688. [DOI: 10.1038/s41598-020-75763-6] | Intervention |
| Tran_2020 | Tran BL, Chen CC, Tseng WC, et al. Tourism under the Early Phase of COVID-19 in Four APEC Economies: An Estimation with Special Focus on SARS Experiences. *Int J Environ Res Public Health*. 2020;17:7543. [DOI: 10.3390/ijerph17207543] | Outcome |
| Trigo_2020 | Godol Trigo LG. Travel and tourism: from imagined scenarios to disruptive events. *Revista Brasileira de Pesquisa em Turismo.*2020;14(3):1-13. [DOI: 10.7784/rbtur.v14i3.2107] | Study type |
| Tsapenko_2020 | Tsapenko IP. The Migration Issue in D. Trump's Coronacrisis Management System [in Russian]. *USA & Canada: economics, polinics, culture*. 2020;50(9):22-43. [DOI: 10Ĵ31857ĺS268667300010963ņ9] | Study type |

Appendix 6 (continued)

| **Study ID** | **Reference** | **Reason for exclusion** |
| --- | --- | --- |
| Ueda_2020 | Ueda M, Stickley A, Sueki H, et al. Mental Health Status of the General Population in Japan during the COVID-19 Pandemic. *Psychiatry and Clinical Neurosciences*. 2020;74(9):505-506. [DOI: 10.1111/pcn.13105] | Intervention |
| Ulak_2020 | Ulak N. COVID-19 Pandemic and its Impact on Tourism Industry in Nepal. *Journal of Tourism & Adventure*. 2020;3(1):50-75. [DOI: 10.3126/jota.v3i1.31356] | Intervention |
| UNODC_2020 | Me A, Zeiler I, Yi JG, et al. COVID-19 and the drug supply chain: from production and trafficking to use. *United Nations Office on Drugs and Crime (UNODC)*. 2020. | Study type |
| VanAssche_2020 | Van Assche A, Lundan S. From the editor: COVID-19 and international business policy. J*ournal of International Business Policy.*2020;3:273–279. [DOI: 10.1057/s42214-020-00065-7] | Study type |
| Vinod_2020 | Vinod B. The COVID‐19 pandemic and airline cash flow. *J Revenue Pricing Manag.*2020;19:228–229. [DOI: 10.1057/s41272-020-00251-5] | Study type |
| Walach_2020 | Walach H, Hockertz S. What association do political interventions, environmental and health variables have with the number of Covid-19 cases and deaths?. A linear modeling approach. *medRxiv [Preprint]*. 2020. [DOI: 10.1101/2020.06.18.20135012] | Outcome |
| Wang_J_2020 | Wang J, Xu X, Wang S, et al. Heterogeneous effects of COVID-19 lockdown measures on air quality in Northern China. *Applied Energy*. 2021;282:116179. [DOI: 10.1016/j.apenergy.2020.116179] | Intervention |
| Wang_L_2020 | Wang L, Wells P. Automobilities after SARS-CoV-2: A Socio-Technical Perspective. *Sustainability*. 2020;12:59785. [DOI: 10.3390/su12155978] | Study type |
| Wang_P_2020 | Wang P, Chen K, Zhu S. Severe air pollution events not avoided by reduced anthropogenic activities during COVID-19 outbreak. *Resources, Conservation and Recycling.*2020;158:104814. [DOI: 10.1016/j.resconrec.2020.104814] | Intervention |
| Wang_Q_2020 | Wang Q, Su M. A preliminary assessment of the impact of COVID-19 on environment – A case study of China. *Sci Total Environ*. 2020;728:138915. [DOI: 10.1016/j.scitotenv.2020.138915] | Study type |
| Weir_2020 | Weir B, Crisp D, O’Dell CW, et al. Regional Impacts of COVID-19 on Carbon Dioxide Detected Worldwide from Space. *arXiv [Preprint]*. 2020. | Intervention |
| Weissgerber_2020 | Weissgerber T, Bediako Y, de Winde CM, et al. Mitigating the impact of conference and travel cancellations on researchers’ futures. *eLife*. 2020;9:e57032. [DOI: 10.7554/eLife.57032] | Study type |
| Wen_2020 | Wen J, Kozak M, Yang S, et al. COVID-19: potential effects on Chinese citizens’ lifestyle and travel. *Tourism Review*. 2020. [DOI: 10.1108/TR-03-2020-0110] | Intervention |
| WHO_2020 | World Health Organization (WHO). Calibrating long-term non-pharmaceutical interventions for COVID-19. *World Health Organization - Western Pacific Region*. 2020. | Study type |
| Wibbens_2020 | Wibbens PD, Koo WWY, McGahan AM. Which COVID policies are most effective? A Bayesian analysis of COVID-19 by jurisdiction. *PLoS ONE*. 2020;15(12): e0244177. [DOI: 10.1371/journal.pone.0244177] | Outcome |
| Wijngaards_2020 | Wijngaards I, Sisouw de Zilwa SCM, Burger MJ. Extraversion Moderates the Relationship Between the Stringency of COVID-19 Protective Measures and Depressive Symptoms. *Front Psychol*. 2020;11:568907. [DOI: 10.3389/fpsyg.2020.568907] | Intervention |

Appendix 6 (continued)

| **Study ID** | **Reference** | **Reason for exclusion** |
| --- | --- | --- |
| Williams_2020 | Williams CC. Impacts of the coronavirus pandemic on Europe's tourism industry: Addressing tourism enterprises and workers in the undeclared economy.*Int J Tourism Res*. 2020;1-10. [DOI: 10.1002/jtr.2395] | Non-Covid-19 |
| Wing_2020 | Wing Shin RT, Stoller C, Woon Yew DL. Issues on the logistics challenges in the pandemic period. *Journal of Critical Reviews.*2020;7(8):776-780. [DOI: 10.31838/jcr.07.08.166] | Study type |
| Wojcieszak-Zbierska_2020 | Wojcieszak-Zbierska MM, Jeczmyk A, Zawadka J, et al. Agritourism in the Era of the Coronavirus (COVID-19): A Rapid Assessment from Poland. *Agriculture*. 2020;10:397. [DOI: 10.3390/agriculture10090397] | Intervention |
| Wong_2020 | Wong B, El-Jack S, Armstrong G. Pandemic control: getting to the heart of unintended consequences. *N Z Med J*. 2020;133(1520):153-156. | Intervention |
| Yang_2020 | Yang Y, Zhang H, Chen X. Coronavirus pandemic and tourism: Dynamic stochastic general equilibrium modeling of infectious disease outbreak. *Ann Tour Res.*2020;83:102913. [DOI: 10.1016/j.annals.2020.102913] | Intervention |
| Yau_2020 | Koh Boon Yau E, Pang Tze Ping N, Shoesmith WD, et al. The Behaviour Changes in Response to COVID-19 Pandemic within Malaysia. *Malays J Med Sci.*2020;27(2):45-50. [DOI: 10.21315/mjms2020.27.2.5] | Study type |
| Yokota_2020 | Yokota I, Shane PY, Okada K, et al. Mass screening of asymptomatic persons for SARS-CoV-2 using saliva. *Clin Infect Dis*. 2020;Sep:ciaa1388. [DOI: 10.1093/cid/ciaa1388] | Outcome |
| Zeng_2021 | Zeng J, Bao R. The impacts of human migration and city lockdowns on specific air pollutants during the COVID-19 outbreak: A spatial perspective. *J Environ Manag*. 2020;282:111907. [DOI: 10.1016/j.jenvman.2020.111907] | Intervention |
| Zerefos_2020 | Zerefos CS, Solomos S, Kapsomenakis J, et al. Lessons learned and questions raised during and post‐COVID‐19 anthropopause period in relation to the environment and climate. *Environ Dev Sustain*. 2020:1-23. [DOI: 10.1007/s10668-020-01075-4] | Intervention |
| Zhai_2020 | Zhai Y, Du X. Mental health for international Chinese students affected by the COVID-19 outbreak. *The Lancet Psychiatry*. 2020;7(4). [DOI: 10.1016/S2215-0366(20)30089-4] | Study type |
| Zhang_X_2020 | Zhang X. Chinese livestock farms struggle under COVID-19 restrictions. Eds. Johan Swinnen and John McDermott. Part Five: Supply chains, Chapter 19:84-85. Washington, DC: *International Food Policy Research Institute (IFPRI)*. 2020. [DOI: 10.2499/p15738coll2.133762_19] | Study type |
| Zheng_2020 | Zheng D, Luo Q, Ritchie BW. Afraid to travel after COVID-19? Self-protection, coping and resilience against pandemic ‘travel fear’. *Tourism Management.*2020;83:104261. [DOI: 0.1016/j.tourman.2020.104261] | Intervention |
| 홍민정_2020 | 홍민정, 오문향. A Study on the Emotional Response of Korean Potential Tourists to the Spread of COVID-19: An application of Semantic Network Analysis. *International Journal of Tourism Management and Sciences*. 2020;35(3):47-65. | Article could not be retrieved |
