# Supplementary material for: Unintended health and societal consequences of international travel measures during the COVID-19 pandemic: a scoping review
Source: J Travel Med. 2021 Aug 9;28(7):taab123. doi: 10.1093/jtm/taab123 (PMC8436381; doi:10.1093/jtm/taab123)
Supplement: Supplementary_Data_Legends_taab123 [file supplementary_data_legends_taab123.docx]

# Supplementary Data

Appendix 1: Review protocol

Appendix 2: Preferred Reporting Items for Systematic reviews and Meta-Analyses extension for Scoping Reviews (PRISMA-ScR) Checklist

Appendix 3: Search strategies and results

Appendix 4: List of reviews, commentaries and discussion papers considered for backward citation tracking

Appendix 5: Data extraction form

Appendix 6: References to studies excluded from this review and reason for exclusion
